# Habitat-Forming Bryozoans in New Zealand: Their Known and Predicted Distribution in Relation to Broad-Scale Environmental Variables and Fishing Effort

**DOI:** 10.1371/journal.pone.0075160

**Published:** 2013-09-23

**Authors:** Anna C. L. Wood, Ashley A. Rowden, Tanya J. Compton, Dennis P. Gordon, P. Keith Probert

**Affiliations:** 1 Department of Marine Science, University of Otago, Dunedin, New Zealand; 2 National Institute of Water and Atmospheric Research, Wellington, New Zealand; 3 Department of Marine Ecology, Royal Netherlands Institute for Sea Research, Den Burg, The Netherlands; Bangor University, United Kingdom

## Abstract

Frame-building bryozoans occasionally occur in sufficient densities in New Zealand waters to generate habitat for other macrofauna. The environmental conditions necessary for bryozoans to generate such habitat, and the distributions of these species, are poorly known. Bryozoan-generated habitats are vulnerable to bottom fishing, so knowledge of species’ distributions is essential for management purposes. To better understand these distributions, presence records were collated and mapped, and habitat suitability models were generated (Maxent, 1 km^2^ grid) for the 11 most common habitat-forming bryozoan species: *Arachnopusia* *unicornis* , *Cellaria* *immersa* , *Cellaria* *tenuirostris* , *Celleporariaagglutinans* , *Celleporinagrandis* , *Cinctipora* *elegans* , *Diaperoecia* *purpurascens* , *Galeopsis* *porcellanicus* , *Hippomenella* *vellicata* , *Hornerafoliacea* , and *Smittoideamaunganuiensis* . The models confirmed known areas of habitat, and indicated other areas as potentially suitable. Water depth, vertical water mixing, tidal currents, and water temperature were useful for describing the distribution of the bryozoan species at broad scales. Areas predicted as suitable for multiple species were identified, and these ‘hotspots’ were compared to fishing effort data. This showed a potential conflict between fishing and the conservation of bryozoan-generated habitat. Fishing impacts are known from some sites, but damage to large areas of habitat-forming bryozoans is likely to have occurred throughout the study area. In the present study, spatial error associated with the use of historic records and the coarse native resolution of the environmental variables limited both the resolution at which the models could be interpreted and our understanding of the ecological requirements of the study species. However, these models show species distribution modelling has potential to further our understanding of habitat-forming bryozoan ecology and distribution. Importantly, comparisons between hotspots of suitable habitat and the distribution of bottom fishing in the study area highlight the need for management measures designed to mitigate the impact of seafloor disturbance on bryozoan-generated habitat in New Zealand waters.

## Introduction

Large, heavily calcified bryozoans >5 cm in 3-dimensions (frame-building bryozoans) are ‘habitat-forming’ when they dominate the seafloor, often as patch reefs, at scales from square metres to hundreds of square kilometres, as single or multiple species [1,2,3]. New Zealand has the most diverse bryozoan-generated habitats, which are among the most expansive in the world’s oceans [4]. These structurally complex habitats support diverse assemblages, particularly of invertebrates (e.g. [2,5]). As relatively large, slow-growing, suspension-feeding organisms, habitat-forming bryozoans are vulnerable to anthropogenic impacts occurring at shelf depths, including fishing [6].

**Figure 1:**
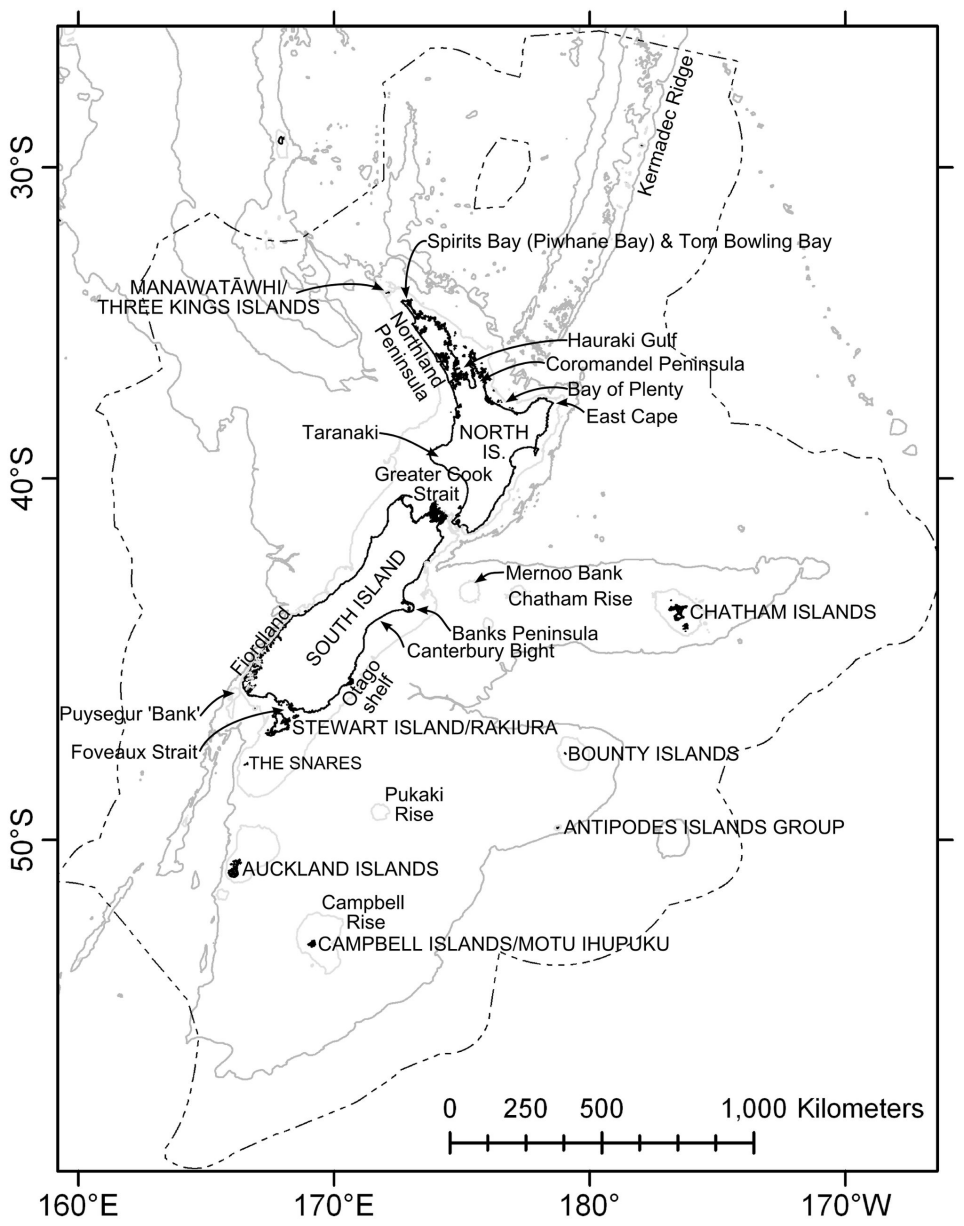
Study site. The New Zealand Extended Continental Shelf (dotted line) showing the 250 m and 2000 m isobaths (solid lines) and location names used in the text. Projection Mercator (WGS84 Datum) at 41° S true latitude and 100° E central meridian [99].

Bryozoans require stable substrata on which to attach, an adequate supply of phytoplankton for food, and low levels of sedimentation and physical disturbance [7]. Specific habitat requirements of habitat-forming bryozoans are poorly understood, but are thought to include high levels of water movement such as those found locally on small (centimetre-to-metre) scale topographic high points [8,9], or on a larger scale, in channels [10,11] and around headlands and peninsulas [12,13]. These conditions reduce the negative effects of sedimentation (e.g. clogging of, or damage to, feeding apparatus) and can result in better access to water-borne food. There may be an association between habitat-forming bryozoans and areas of high productivity; high bryozoan diversity is associated with upwelling and frontal features [14], and some of these high diversity areas are also locations where bryozoans generate habitat. Such habitat is known from the continental shelf throughout New Zealand’s Extended Continental Shelf area (ECS, Figure 1) (e.g. [13,15,16]), and although shelf depths have been well-studied, sampling has not been exhaustive and bryozoan-generated habitats may exist, or have existed, elsewhere in the ECS. Knowing where such habitat occurs is essential to its successful management.

Commercially important and bycatch species (*sensu* [17]) use bryozoans and their associated assemblages as habitat, nursery grounds and for feeding [18,19], and known areas of habitat-forming bryozoans in New Zealand have been associated with commercial fishing activities for scallops ( *Zygochlamys* *delicatula* , *Pecten novaezelandiae*), snapper ( *Sparus* *aurata* ), tarakihi ( *Nemadactylus* *macropterus* ), and oysters ( *Ostrea* *chilensis* ), amongst others [12,16,20,21,22]. Trawling and dredging damage benthic organisms [23,24], reduce or alter the composition of local biodiversity, and may change ecosystem function [25]. Commercial fishing has destroyed large areas of habitat-forming bryozoans [16], and is implicated in their loss/reduction elsewhere in the New Zealand ECS [12,20,22].

The management of bryozoan-generated habitat by the New Zealand Ministry for Primary Industries has varied from establishing protected areas at the request of fishers [26], to agreeing to the existence of (but not monitoring or listing) voluntary protected areas, to favouring continued fishing over direct conservation measures such as spatial closures (e.g. oyster dredging in Foveaux Strait, http://fs.fish.govt.nz/Page.aspx?pk=113&dk=22242). To achieve management goals and adhere to guiding principles (see the New Zealand Fisheries Act 1996, and [27,28]), it is essential to identify areas where there is likely to be a conflict between the conservation of a significant habitat type, and the continuation of commercial fishing interests.

Presence-only machine-learning modelling methods such as Maxent [29] can be used to generate full-coverage prediction maps of potentially suitable habitat for a species based on recorded observations of that species (point data), and information about the environment in which the records occur. Such methods have the advantage of not requiring absence records (see [30]), meaning that presence records held by museums and research institutions can be used to guide conservation efforts where no other information is available [29]. Maxent is one of the best-performing predictive modelling methods for presence-only data, particularly where data are few [30,31,32], and has been used to predict suitable habitat for vulnerable taxa such as cold-water corals [33,34,35], thereby providing information to guide future sampling, allow the evaluation of spatial closures designed to protect such taxa, and guide management aimed to mitigate impacts (fishing, mining, climate change, ocean acidification) upon seafloor assemblages [33].

The present study aimed to: (1) describe the known distribution of bryozoan species that can generate habitat in the New Zealand ECS; (2) use these distribution records, together with pre-existing information describing aspects of the environment, to model the potential distribution of suitable habitat for these species; (3) identify the broad-scale environmental conditions underlying the predicted distributions of these species; and (4) identify ‘hotspots’ of suitable habitat for bryozoans (areas where they have the potential to be ‘habitat-forming’), and compare these with the distribution of fishing effort, to highlight areas where there may be a conflict between conserving this significant habitat type, and the continuation of fishing interests in the New Zealand ECS.

## Methods

### Species data

The study area comprised the New Zealand ECS (5.8 million km^2^, Figure 1). Twenty-seven species of bryozoans are known to generate habitat in this region [4]. Records of these species, all identified by a bryozoologist (D. Gordon), were collated from a variety of sources to form the primary dataset. These data represent sampling over the years 1901–2011 using a variety of collection methods, predominantly dredges, grabs, SCUBA, corers, but also trawls, shore collections, and epibenthic sleds (Table S1). Species with a minimum of 20 records were selected, because too few records can result in models that predict too widely [36], but requiring a greater number of records would have severely limited the number of species for which habitat suitability could be predicted. The known distributions were mapped in ArcGIS 10.0 (www.esri.com), and then Maxent software for species habitat modelling (version 3.3.3k [29]) was used to predict the potential distribution of suitable habitat for the study species at a spatial resolution of 1 km^2^. The primary data set also comprised target-group background (TGB) data. These were records of bryozoans of all growth forms, from throughout the ECS, collated from the same surveys as those from which the presence records were obtained, and which were also identified by D. Gordon; these TGB data were used to address sampling bias [37,38]. Records of the study species which had not been verified by D. Gordon were collated from the published literature in a secondary data set, and used to visually assess the predictive models, after model training and testing using the primary data set.

Inspection showed there were a few presence records (>1 per species) in water depths of >1000 m (up to ~5000 m). In published literature, however, habitat-forming bryozoan species have not been recorded in water depths >950 m [4,39]. The validity of these data could not be assessed, so with the aim of producing accurate yet conservative predictions, both presence and TGB data from sites >2000 m water depth were removed from the data set, and a ‘mask’ was created to prevent Maxent from predicting to areas >2000 m. This depth was chosen as a trade-off between the depths recorded in the primary data set, and those previously recorded in the literature, to allow Maxent to predict habitat in potentially under-sampled areas, whilst maintaining realistic boundaries on the study area.

Eleven bryozoan species known to generate habitat had ≥20 records, after duplicate records in each 1 km^2^ cell of the study area were removed. These species were: *Arachnopusia* *unicornis* , *Cellaria* *immersa* , *Cellaria* *tenuirostris* , *Celleporariaagglutinans* , *Celleporinagrandis* , *Cinctipora* *elegans* , *Diaperoecia* *purpurascens* , *Galeopsis* *porcellanicus* , *Hippomenella* *vellicata* , *Hornerafoliacea* , and *Smittoideamaunganuiensis* . Data for each presence and TGB record (latitude, longitude and depth, see Table S1) were collated, and where these data were not available, a nominal co-ordinate was allocated using Google Maps (www.maps.google.com/).

### Environmental data

Environmental data describing the known and probable broad ecological requirements of habitat-forming bryozoans were available in the form of 16 gridded, geo-referenced layers constructed from interpolations and models. The default options in Maxent were ‘tuned’ using 11–13 such layers [37,40], so it was necessary to reduce the number of layers used in the present study. Values from the layer describing depth sometimes differed significantly from those recorded at the time of sampling (Table S1), particularly for sites of steep topography. This disparity meant using variables that included the depth layer in their calculation carried a risk of introducing systematic inaccuracies, and initial models showed some of these variables (e.g. slope) also made little or no contribution to the models. With the aim of reducing potential error and producing parsimonious models, layers which used depth in their calculation (slope, bottom water temperature, mean peak bed orbital velocity, particulate organic carbon flux) were removed. Further, layers which described a similar variable as another (sea surface temperature annual amplitude and coloured dissolved organic matter) were also removed, again with the aim of achieving parsimonious models. The 10 remaining environmental layers were used in all models (see Table 1 and Figure S1 for layer source and development).

**Table 1:** Description and source of the environmental variable layers used to model the distribution of habitat-forming bryozoans, showing the source of the original version, although the most up-to-date versions were used.

**Variable name**	**Units**	**Native data resolution**	**Description**	**Source**
Depth (Depth)		1 km^2^	Depth at the seafloor based on available bathymetric coverage	[41,42]
Sea surface temperature in winter (SSTWinter)	°C	9 km^2^	Sea surface temperature when it is typically lowest (day 250, early September)	[41]
Annual average wave height (WaveHeight)	m	2°	Annual average wave height derived from hindcast (1979-1998) of swell wave conditions in the New Zealand Exclusive Economic Zone and a hindcast model covering the southwestern Pacific and Southern Oceans	[47]
Depth averaged maximum tidal current (TidalCurrent)	m s^-1^	2-50 km^2^ (depth dependent)	Maximum depth-averaged tidal current velocity estimated by interpolating outputs from the New Zealand region tide model	[41,48]
Annual mean mixed layer depth (MLD)	m	0.5°	Mixed layer depth of the water column estimated by interpolating measures of mixed layer depth calculated from CTD profiles using difference criteria for temperature and salinity	http://www.marine.csiro.au/~dunn/carS2009/
Sea surface temperature gradient (SSTGradient)	°C km^-1^	9 km^2^	Smoothed annual mean SST spatial gradient estimated from remotely sensed SeaWiFS data	[41,53]
Surface water primary productivity (Productivity)	mg C m^-2^ d^-1^	~9 km^2^	Vertically generalised production model based on net primary productivity estimated as a function of remotely sensed chlorophyll, irradiance, and photosynthetic efficiency estimated from remotely sensed sea surface temperatures	[51]
Total suspended particulate matter concentration (SPM)	g m^-3^	~4 km^2^	Based on SeaWiFS ocean colour remote sensing data; modified Case 2 atmospheric correction; modified Case 2 inherent optical property algorithm	[55,56]
Dominant sediment class (SedType)	category	3-15 km^2^	1: deep ocean clays, 2: calcareous gravel, 3: volcanic, 4: calcareous mud, 5: gravel, 6: mud, 7: sand, 8: calcareous sand, 9: clay, collated from available sediment charts	[57]
Median grain size (MGS)	mm	3-15 km^2^	Median sediment grain size collated from available sediment charts	[57]

Depth itself was included as a layer [41,42], because the disparities between recorded and layer depth could be addressed by replacing layer depth values with recorded depth values for both presence and TGB data. Recorded depths were used for >90% of presence records, and >87% of TGB records (see Table S1). Where no measured depth value was available, the layer value for depth was used. Depth represents a synthesis of variables likely to affect bryozoan distribution, including wave disturbance [43,44], water temperature [45], sediment inputs from land [46], and food availability [14,44], all of which decrease with depth. As such, depth is a useful variable to include in predictive models (e.g. [35]), even though the precise nature of the relationship between any one of the correlated variables and the distribution of a species can only be revealed by including layers for each individual variable. Annual average wave height [47] and depth averaged maximum tidal current [41,48] describe wave and tide induced water motion, which are likely to be important to bryozoans in terms of disturbance [43,44] and food transport [13,14,41]. Water motion at the seafloor induced by surface waves is unlikely to occur beyond the shelf break, but water movement resulting from tides can occur in deeper water (see TidalCurrent in Figure S1). Temperature affects species distribution because of its (species-specific) influence on, for example, feeding rate [49], so sea surface temperature (SST) in winter [41] (i.e. minimum temperature) was included as a layer. Surface temperatures (as opposed to temperatures at depth) are again appropriate here, given the shelf-depth distribution of the majority of presence records, and the well-mixed nature of shelf-depth waters in New Zealand [50]. Food availability is important in determining habitat suitability for benthic organisms (e.g. [14]), and three environmental layers were used to describe aspects of this variable: surface water primary productivity [51] was derived from a vertically generalised productivity model based on remotely sensed estimates of chlorophyll *a* concentration [51]; mixed layer depth (www.marine.csiro.au/~dunn/carS2009/) describes broad-scale nutrient availability and productivity, and the availability of labile nutrients to the benthic community [52]; and SST gradient [41,53] describes oceanic frontal zones [41] which are often associated with increased surface productivity, and enhanced biogenic flux to the benthos (e.g. [54]). Bryozoans can be susceptible to the negative effects of suspended sediments [46], thus, total suspended particulate matter concentration [55,56] was used as a measure of the influence of this variable (particularly with respect to river discharges). The distribution of benthic organisms is greatly influenced by substratum type and frame-building bryozoans require a suitable substratum on which to attach [7]. Two layers were used to describe the nature of the seafloor: median grain size and dominant (>50%) sediment type [57]. These layers were developed from sediment charts [57], and vary spatially in the level of detail available, being most detailed at shelf depths and in specific areas (e.g. Banks Peninsula, Foveaux Strait), and less informative in very shallow water (<10 m). Despite these limitations, the sediment layers used provide the best available representation of a potentially important variable, which preliminary models showed improved model accuracy for some species. Environmental data layer values for presence and TGB sample sites were assessed for multicollinearity (Pearson correlations) to aid model interpretation.

All variables were gridded at 1 km^2^ resolution in ArcGIS, and mapped in a World Mercator Projection (central meridian 100° E, standard parallel 41° S). Values for each cell in which a presence or TGB record occurred were extracted (worksheets Presence and TGB in Table S2) from the gridded environmental layers using the ‘intersect point tool’ from Hawth’s tools (www.spatialecology.com/htools) in ArcGIS. The environmental layers had the same outer extent (the ECS), but layer extents in coastal areas varied so that some presence records lay ‘outside’ some layers. Where a presence record fell outside all layers, the record was moved to the environmental raster point of closest alignment so that a value was obtained for one or more layers, otherwise a ‘NoData’ value was used. Box-and-whisker plots were generated from these data for each species, to illustrate the environmental range occupied (Figure S2).

### Model settings and outputs

The algorithms and processes underlying Maxent have been well described [29,31,58]. Briefly, Maxent uses presence records and features (linear, quadratic, or product functions derived from the environmental data and shown as fitted response curves), to predict the distribution of suitable habitat for the species across the study area by finding the distribution of maximum entropy (the distribution closest to uniform), subject to the constraint that the expected value of each feature under this estimated distribution matches its empirical average [31].

Models in the present study were generated using default settings, including logistic output and regularisation (to ensure parsimonious models [29,40,59]), with additional options selected. These caused Maxent to: use samples with some missing data; use cross-validation (replicates = 10) to make the best use of small data sets; undertake jackknifes to determine variable importance; and generate response curves. Using samples with some missing data caused Maxent to use all of the available data in the construction of features, but when generating predictions, to show ‘NoData’ for any cell in which a value was missing (S. Phillips personal communication 07/05/2008). For cross-validation, presence data were split randomly into 10 ‘folds’, of which 9 were used to train the model (develop parameters) and 1 was used for testing using the parameters found during training. This process was repeated until all folds had been used for both training and testing. Mean models were presented, and validation statistics were calculated. Validation statistics were model omission, test gain and area under the receiver-operator curve (AUC). Model omission is the misclassification of a cell containing a presence record as one that is unsuitable habitat for that species. The omission rate of test records was assessed using ‘Fixed cumulative value 10 test omission’, which should ideally lead to omission levels of about 10% [60]. Gain is closely related to deviance, a measure of goodness of fit used in generalised additive and generalised linear models, and within Maxent is defined as the average log probability of the presence samples, minus a constant (regularisation) that makes the uniform distribution have zero gain. Gain indicates how closely the model is concentrated around the presence samples; for example, if the gain is 2, the average likelihood of the presence samples is exp (2) ^≈^ 7.4 times higher than that of a random background cell (Maxent tutorial at http://www.cs.princeton.edu/~schapire/maxent). The AUC value indicates how well the model fits the data by measuring the probability that a presence record will be ranked higher than a background record [61]. A test AUC <0.5 meant the model was no better than random and models with AUC >0.7 were considered useful [62,63]. AUC is widely used in habitat suitability modelling, but has also been criticised as an inappropriate use of the statistic [64], hence model omission and test gain were also considered.

Jackknifes provide alternative, visual estimates of variable importance, by showing the relative effect of each variable on regularised training gain [29], test gain, and AUC. Jackknifes were obtained by excluding each variable in turn and creating a model with the remaining variables, then by creating a model using each variable in isolation and comparing these against the full model. Response curves showed how predicted habitat suitability changed at different values of the variable, both at average values of other variables (marginal) and for each variable on its own (individual). Based on these response curves Maxent predicted the relative suitability of each cell in the study area. These predictive maps have a minimum habitat suitability threshold of the 10^th^ percentile presence value, a conservative threshold which can account for potential errors in collection data [34] by excluding 10% of ‘outliers’. The importance of each environmental variable to the final model is shown as permutation importance, which was determined by randomly permuting the values of that variable among the training points (both presence and background) and measuring the resulting decrease in training AUC (Maxent tutorial at www.cs.princeton.edu/~schapire/maxent).

To identify cells in which multiple species were likely to find suitable habitat (‘hotspots’), binary versions of the habitat suitability maps were generated for each species (not shown), again using the 10^th^ percentile training presence threshold. Only species for which cross-validation had produced AUC values >0.7 were included, and default settings were used, again with the option selected to use samples with some missing data. Thresholded predictions were imported as rasters to ArcGIS, and the value of each cell was summed across the rasters, producing one map to summarise the distribution of suitable habitat.

## Results

**Figure 2:**
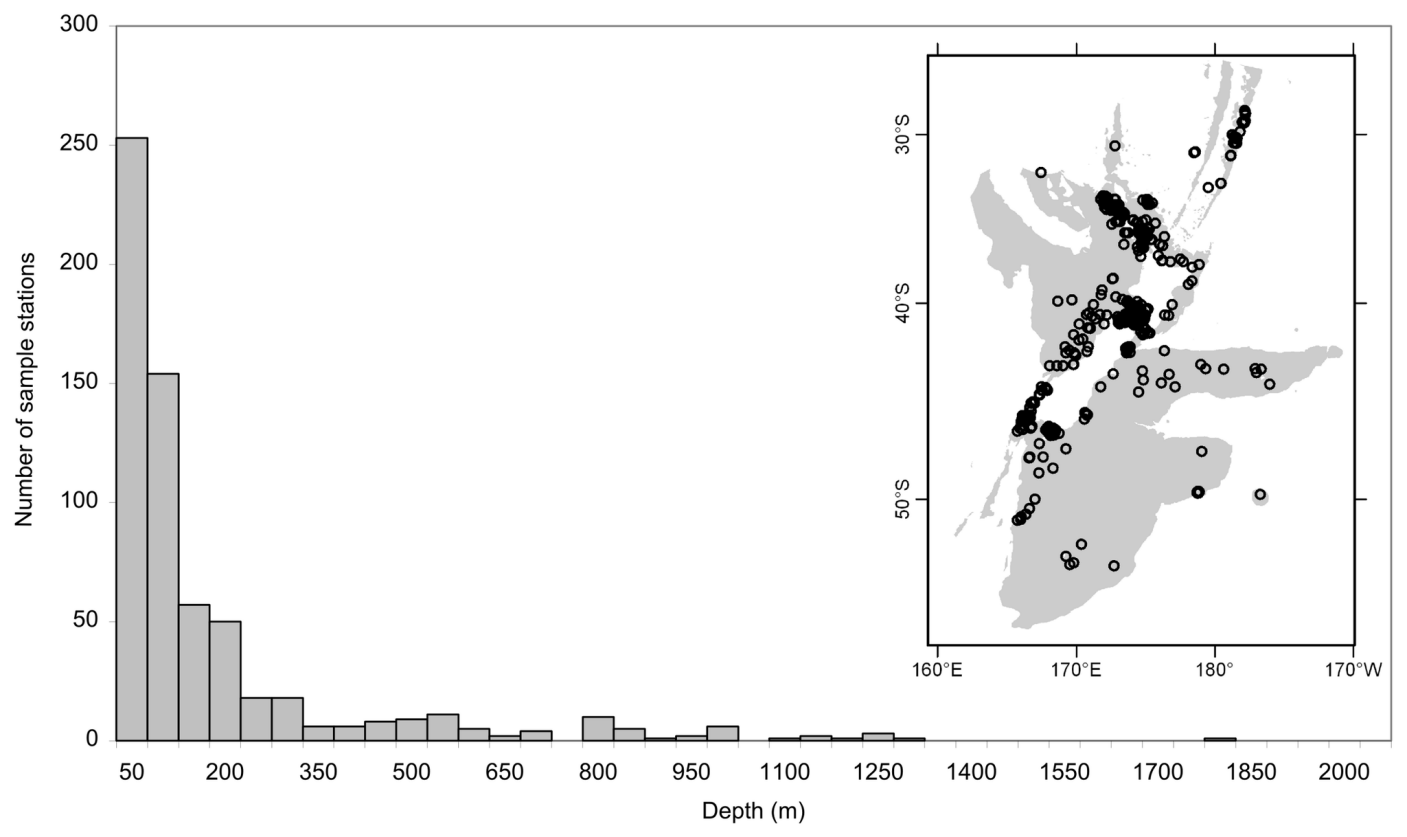
Summary distribution of collated bryozoan records. Frequency of target-group background sampling (including presence points) in 50 m depth classes. The insert shows the geographic distribution of these sites, with gray shading showing water depths <2000 m, beyond which samples were excluded.

**Figure 3:**
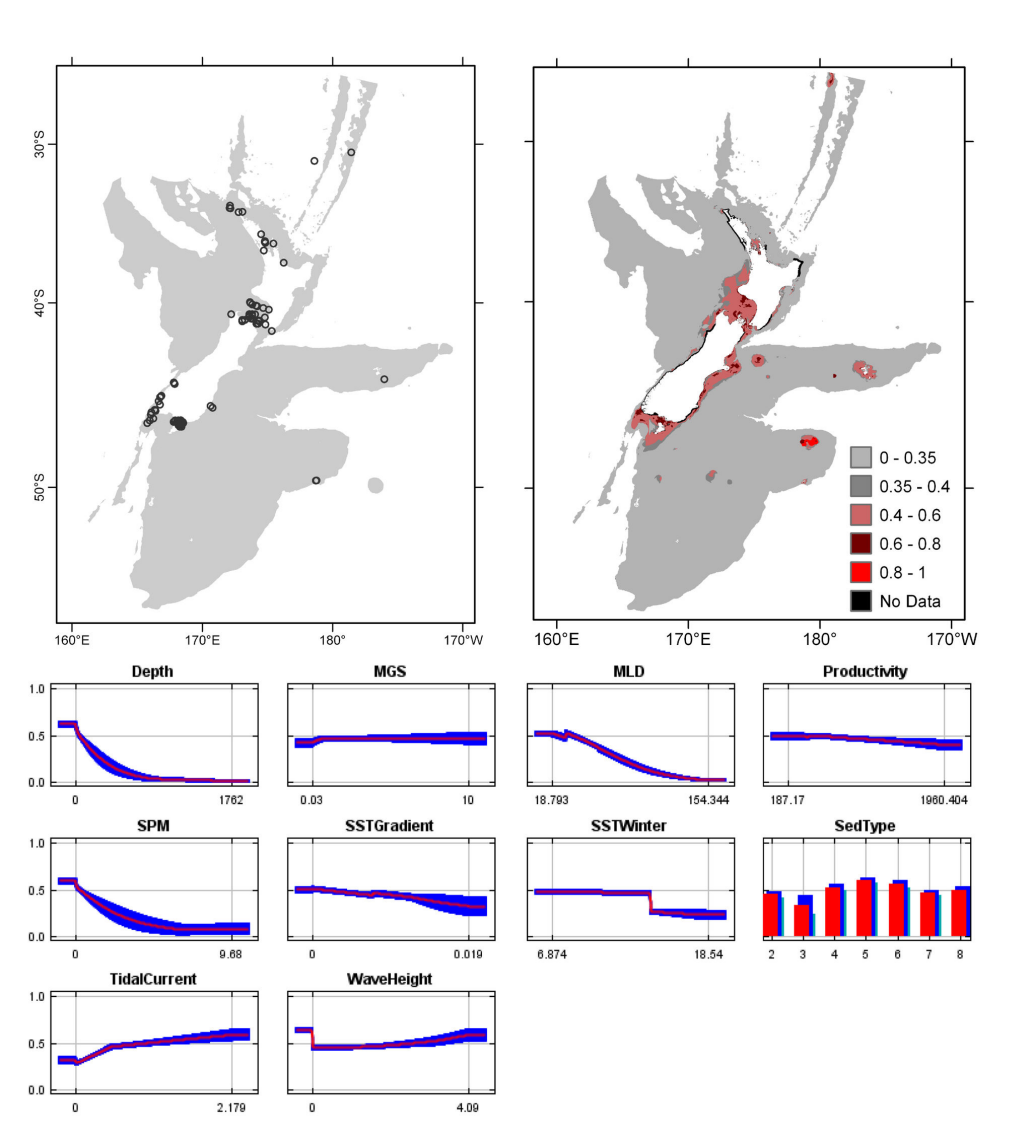
*Arachnopusia* *unicornis* known distribution (left), predicted suitable habitat (right), and fitted responses curves (marginal). For the predicted distribution, logistic probabilities less than the 10^th^ percentile presence value indicated cells were unsuitable habitat. Probabilities of 0.4–0.6 indicated habitat suitability typical of the presence records, values of 0.6–0.8 and 0.8–1 indicated favourable and highly suitable habitat, respectively. Black cells had missing data in one or more environmental layer. Independent records are layed over the predictions. Marginal response curves show how the prediction changes for different values of each variable when all other variables were at their average sample value. Individual response curves are shown in Figure S3. For the categorical variable Sediment type: 1 = deep ocean clays; 2 = calcareous gravel; 3 = volcanic; 4 = calcareous mud; 5 = gravel; 6 = mud; 7 = sand; 8 = calcareous sand.

**Figure 4:**
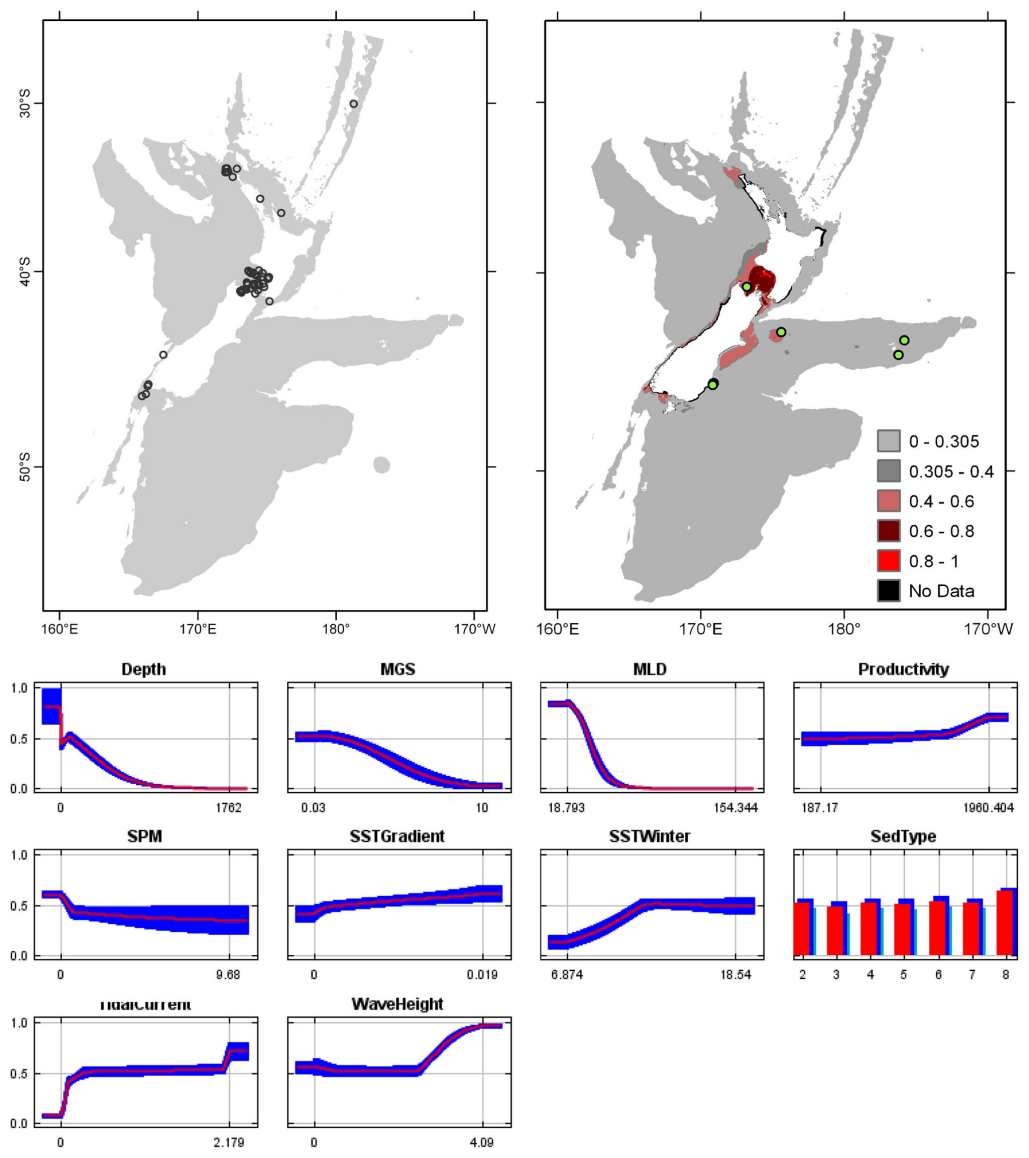
*Cellaria* *immersa* known distribution (left), predicted suitable habitat (right), and fitted responses curves (marginal). For the predicted distribution, logistic probabilities less than the 10^th^ percentile presence value indicated cells were unsuitable habitat. Probabilities of 0.4–0.6 indicated habitat suitability typical of the presence records, values of 0.6–0.8 and 0.8–1 indicated favourable and highly suitable habitat, respectively. Black cells had missing data in one or more environmental layer. Independent records are layed over the predictions. Marginal response curves show how the prediction changes for different values of each variable when all other variables were at their average sample value. Individual response curves are shown in Figure S3. For the categorical variable Sediment type: 1 = deep ocean clays; 2 = calcareous gravel; 3 = volcanic; 4 = calcareous mud; 5 = gravel; 6 = mud; 7 = sand; 8 = calcareous sand.

**Figure 5:**
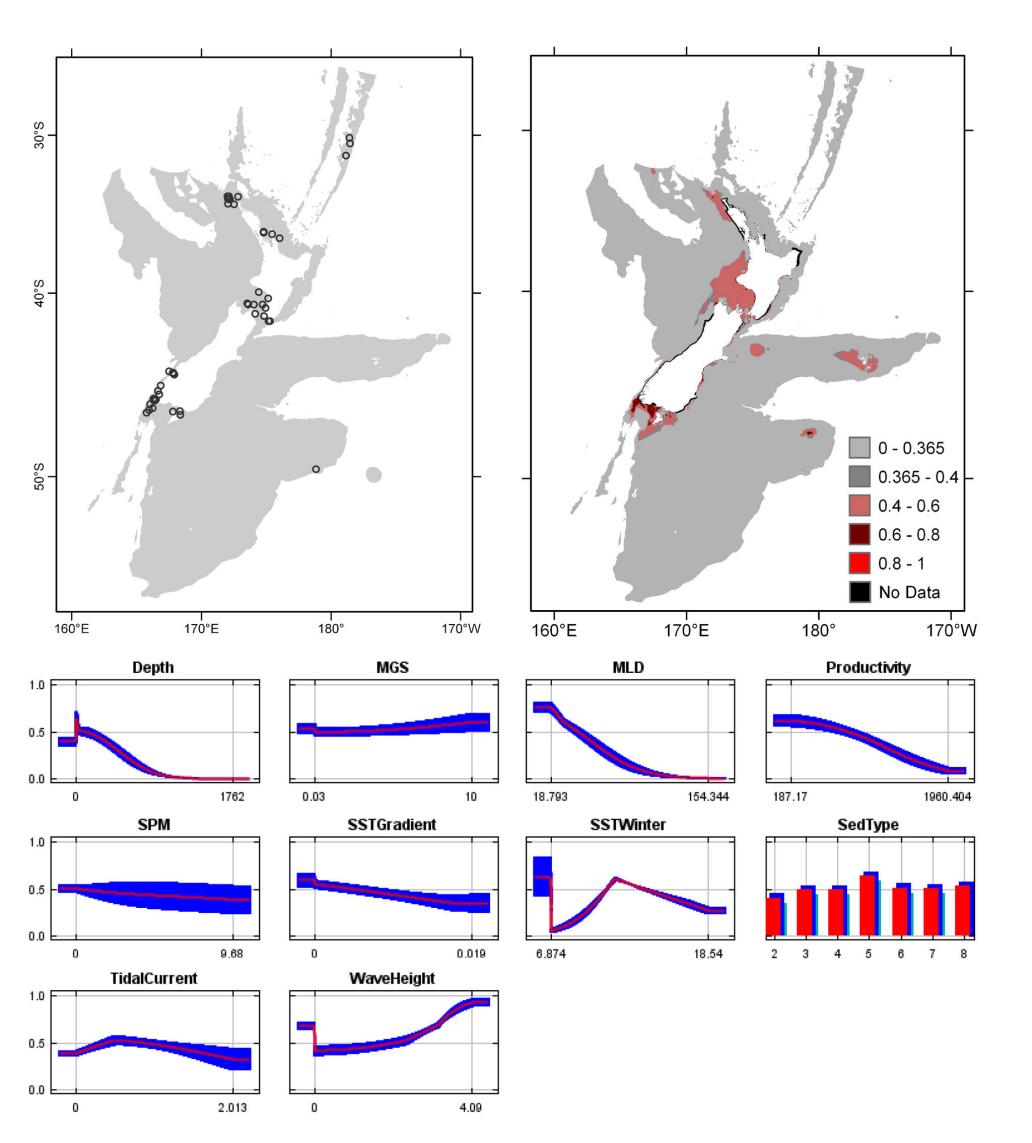
*Cellaria* *tenuirostris* known distribution (left), predicted suitable habitat (right), and fitted responses curves (marginal). For the predicted distribution, logistic probabilities less than the 10^th^ percentile presence value indicated cells were unsuitable habitat. Probabilities of 0.4–0.6 indicated habitat suitability typical of the presence records, values of 0.6–0.8 and 0.8–1 indicated favourable and highly suitable habitat, respectively. Black cells had missing data in one or more environmental layer. Independent records are layed over the predictions. Marginal response curves show how the prediction changes for different values of each variable when all other variables were at their average sample value. Individual response curves are shown in Figure S3. For the categorical variable Sediment type: 1 = deep ocean clays; 2 = calcareous gravel; 3 = volcanic; 4 = calcareous mud; 5 = gravel; 6 = mud; 7 = sand; 8 = calcareous sand.

**Figure 6:**
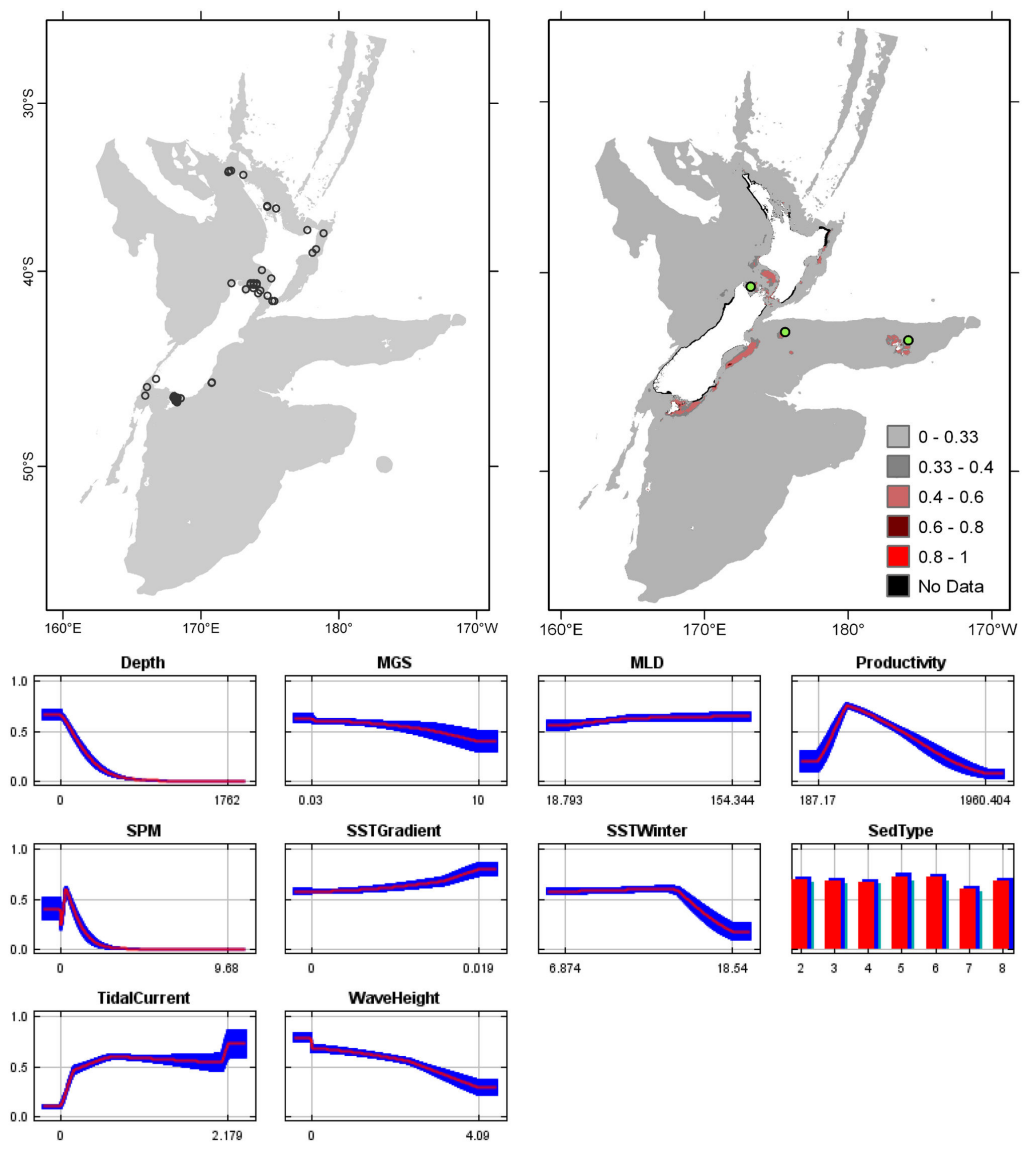
*Celleporariaagglutinans* known distribution (left), predicted suitable habitat (right), and fitted responses curves (marginal). For the predicted distribution, logistic probabilities less than the 10^th^ percentile presence value indicated cells were unsuitable habitat. Probabilities of 0.4–0.6 indicated habitat suitability typical of the presence records, values of 0.6–0.8 and 0.8–1 indicated favourable and highly suitable habitat, respectively. Black cells had missing data in one or more environmental layer. Independent records are layed over the predictions. Marginal response curves show how the prediction changes for different values of each variable when all other variables were at their average sample value. Individual response curves are shown in Figure S3. For the categorical variable Sediment type: 1 = deep ocean clays; 2 = calcareous gravel; 3 = volcanic; 4 = calcareous mud; 5 = gravel; 6 = mud; 7 = sand; 8 = calcareous sand.

**Figure 7:**
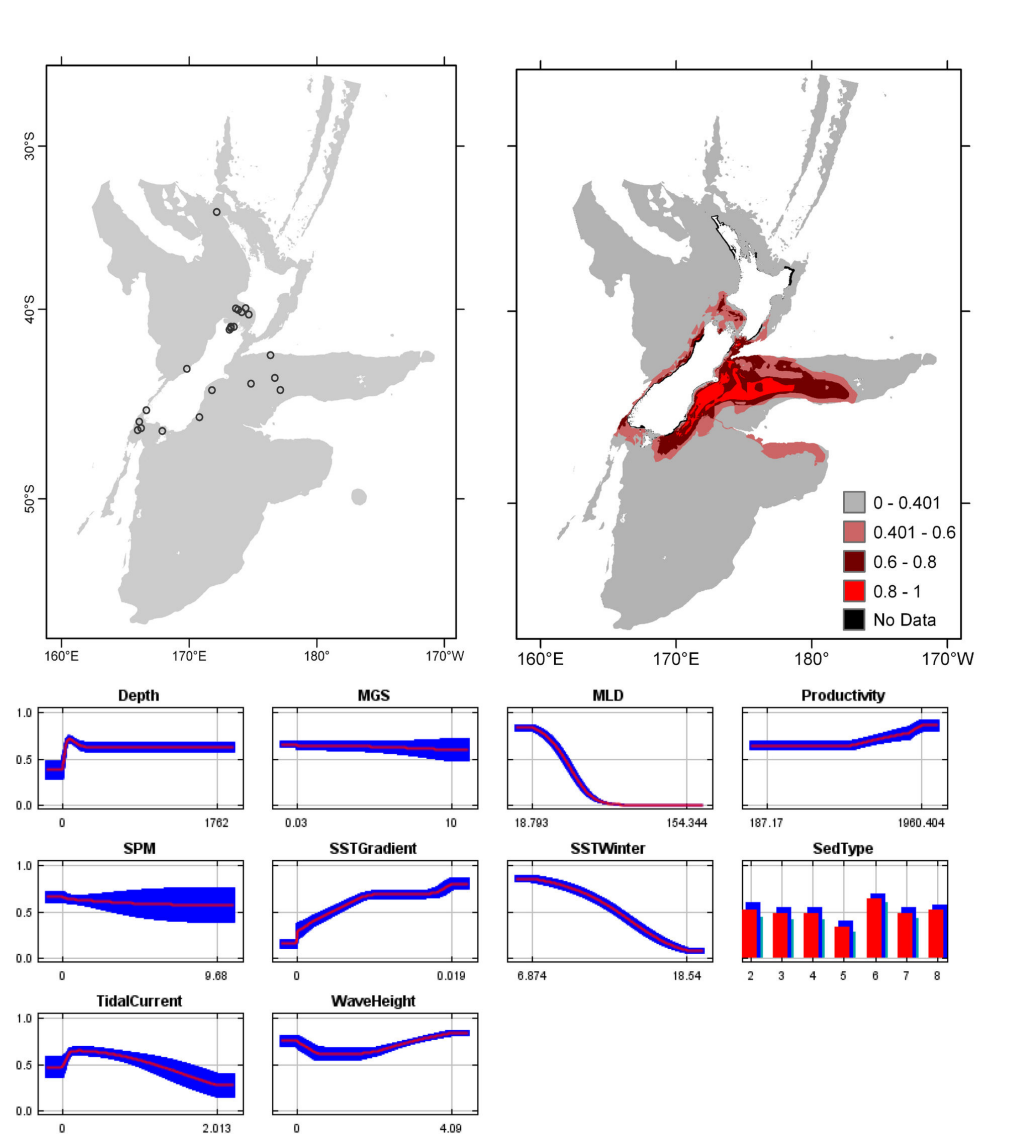
*Celleporinagrandis* known distribution (left), predicted suitable habitat (right), and fitted responses curves (marginal). For the predicted distribution, logistic probabilities less than the 10^th^ percentile presence value indicated cells were unsuitable habitat. Probabilities of 0.4–0.6 indicated habitat suitability typical of the presence records, values of 0.6–0.8 and 0.8–1 indicated favourable and highly suitable habitat, respectively. Black cells had missing data in one or more environmental layer. Independent records are layed over the predictions. Marginal response curves show how the prediction changes for different values of each variable when all other variables were at their average sample value. Individual response curves are shown in Figure S3. For the categorical variable Sediment type: 1 = deep ocean clays; 2 = calcareous gravel; 3 = volcanic; 4 = calcareous mud; 5 = gravel; 6 = mud; 7 = sand; 8 = calcareous sand.

**Figure 8:**
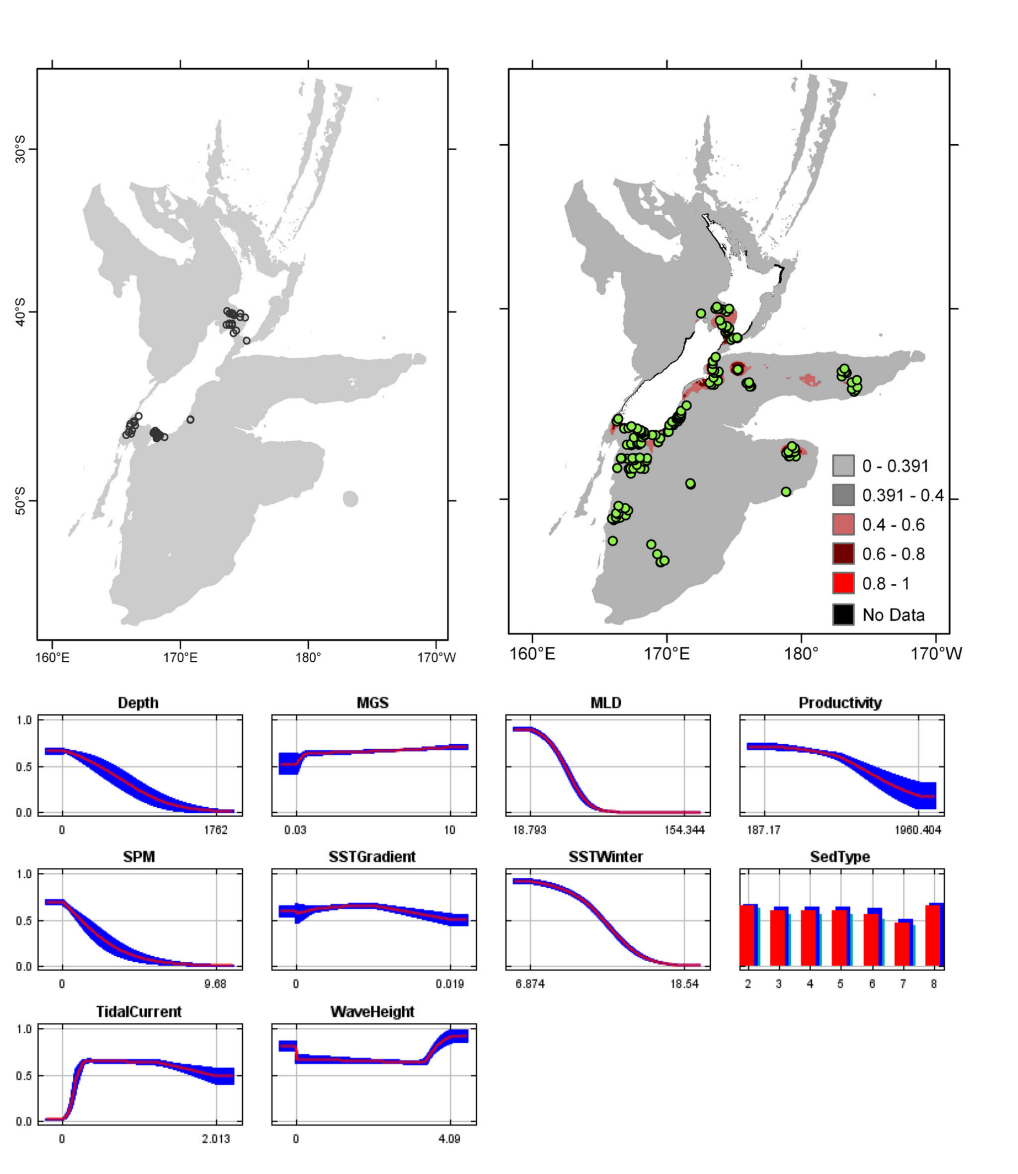
*Cinctipora* *elegans* known distribution (left), predicted suitable habitat (right), and fitted responses curves (marginal). For the predicted distribution, logistic probabilities less than the 10^th^ percentile presence value indicated cells were unsuitable habitat. Probabilities of 0.4–0.6 indicated habitat suitability typical of the presence records, values of 0.6–0.8 and 0.8–1 indicated favourable and highly suitable habitat, respectively. Black cells had missing data in one or more environmental layer. Independent records are layed over the predictions. Marginal response curves show how the prediction changes for different values of each variable when all other variables were at their average sample value. Individual response curves are shown in Figure S3. For the categorical variable Sediment type: 1 = deep ocean clays; 2 = calcareous gravel; 3 = volcanic; 4 = calcareous mud; 5 = gravel; 6 = mud; 7 = sand; 8 = calcareous sand.

**Figure 9:**
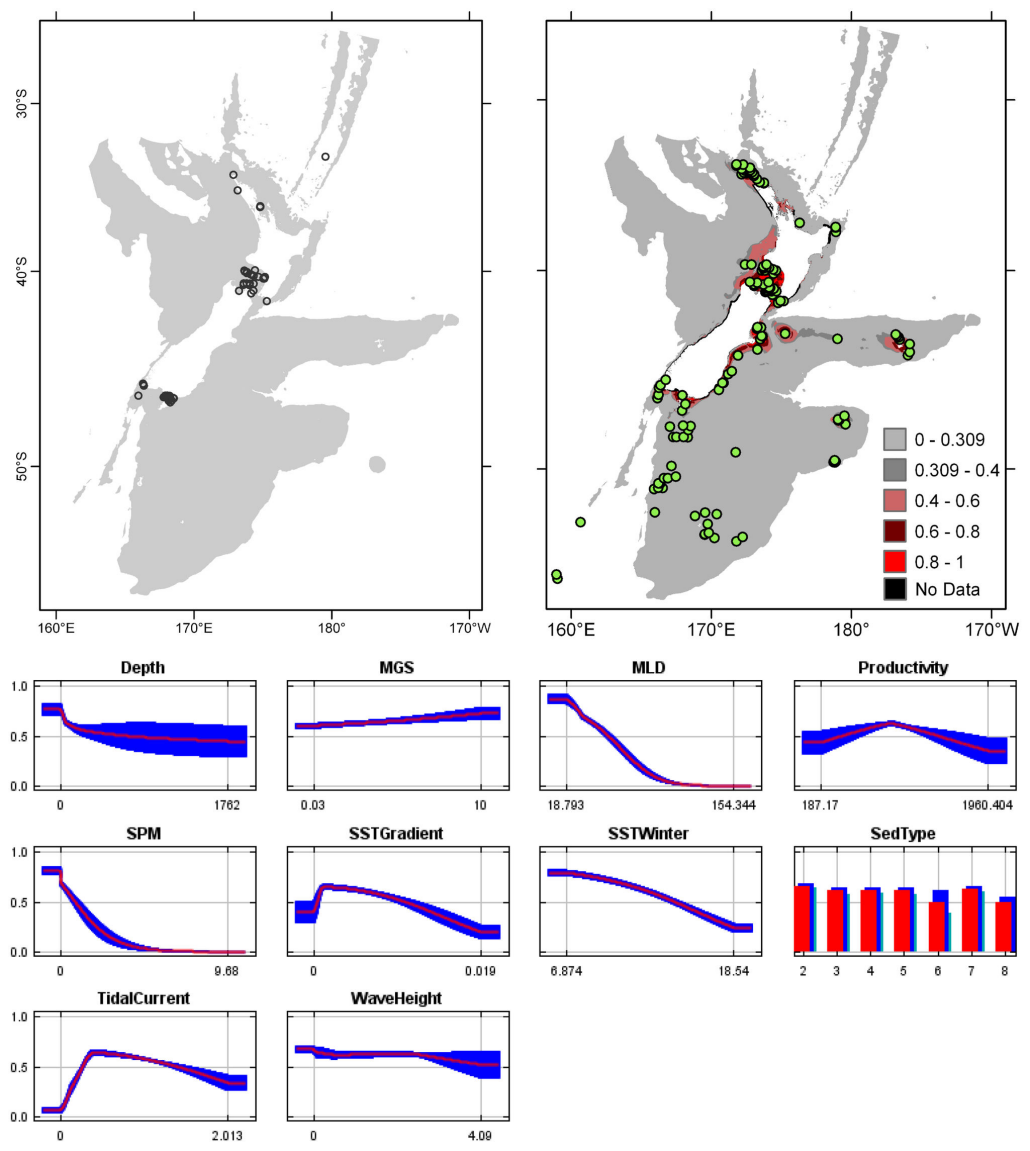
*Diaperoecia* *purpurascens* known distribution (left), predicted suitable habitat (right), and fitted responses curves (marginal). For the predicted distribution, logistic probabilities less than the 10^th^ percentile presence value indicated cells were unsuitable habitat. Probabilities of 0.4–0.6 indicated habitat suitability typical of the presence records, values of 0.6–0.8 and 0.8–1 indicated favourable and highly suitable habitat, respectively. Black cells had missing data in one or more environmental layer. Independent records are layed over the predictions. Marginal response curves show how the prediction changes for different values of each variable when all other variables were at their average sample value. Individual response curves are shown in Figure S3. For the categorical variable Sediment type: 1 = deep ocean clays; 2 = calcareous gravel; 3 = volcanic; 4 = calcareous mud; 5 = gravel; 6 = mud; 7 = sand; 8 = calcareous sand.

**Figure 10:**
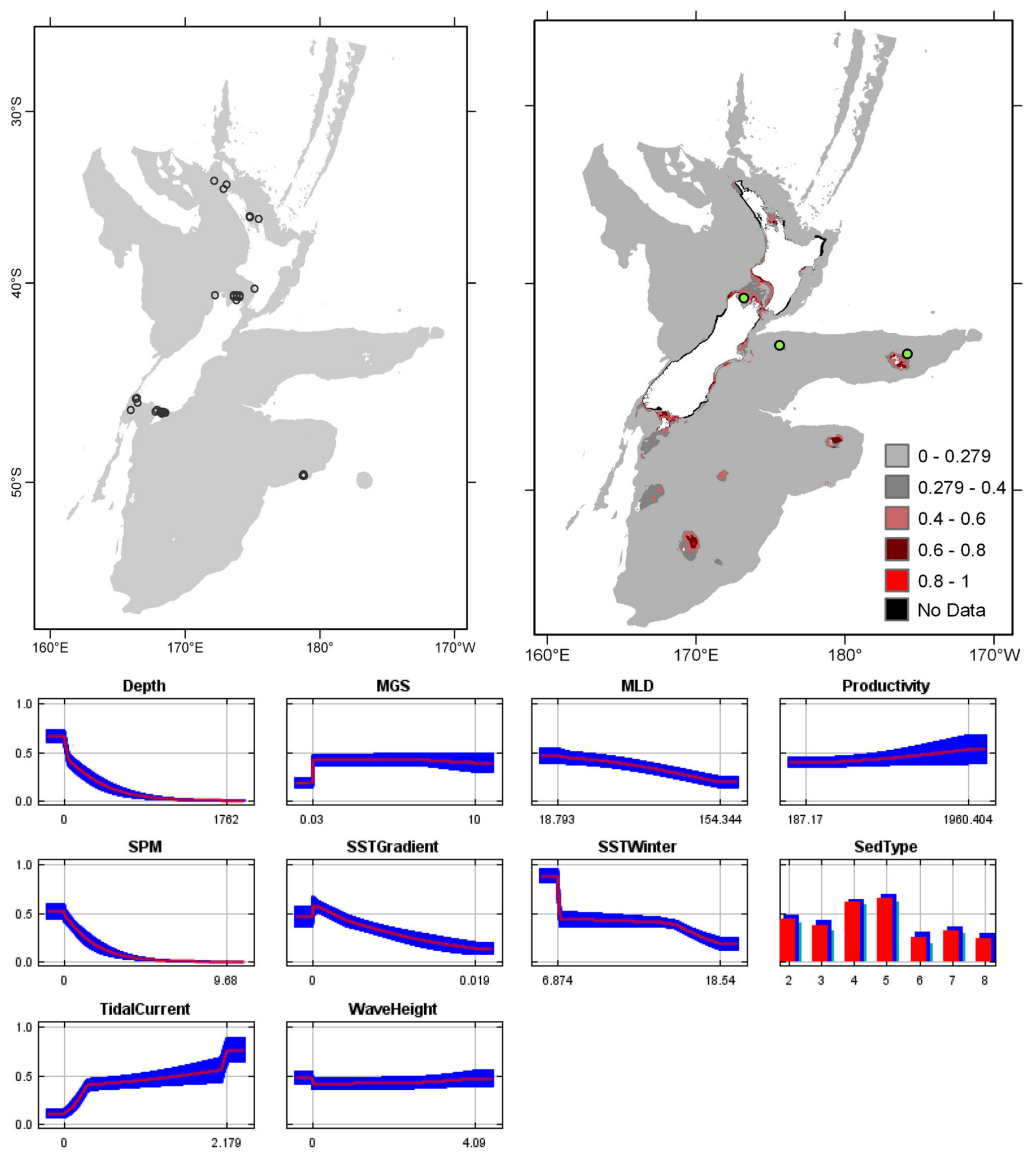
*Galeopsis* *porcellanicus* known distribution (left), predicted suitable habitat (right), and fitted responses curves (marginal). For the predicted distribution, logistic probabilities less than the 10^th^ percentile presence value indicated cells were unsuitable habitat. Probabilities of 0.4–0.6 indicated habitat suitability typical of the presence records, values of 0.6–0.8 and 0.8–1 indicated favourable and highly suitable habitat, respectively. Black cells had missing data in one or more environmental layer. Independent records are layed over the predictions. Marginal response curves show how the prediction changes for different values of each variable when all other variables were at their average sample value. Individual response curves are shown in Figure S3. For the categorical variable Sediment type: 1 = deep ocean clays; 2 = calcareous gravel; 3 = volcanic; 4 = calcareous mud; 5 = gravel; 6 = mud; 7 = sand; 8 = calcareous sand.

**Figure 11:**
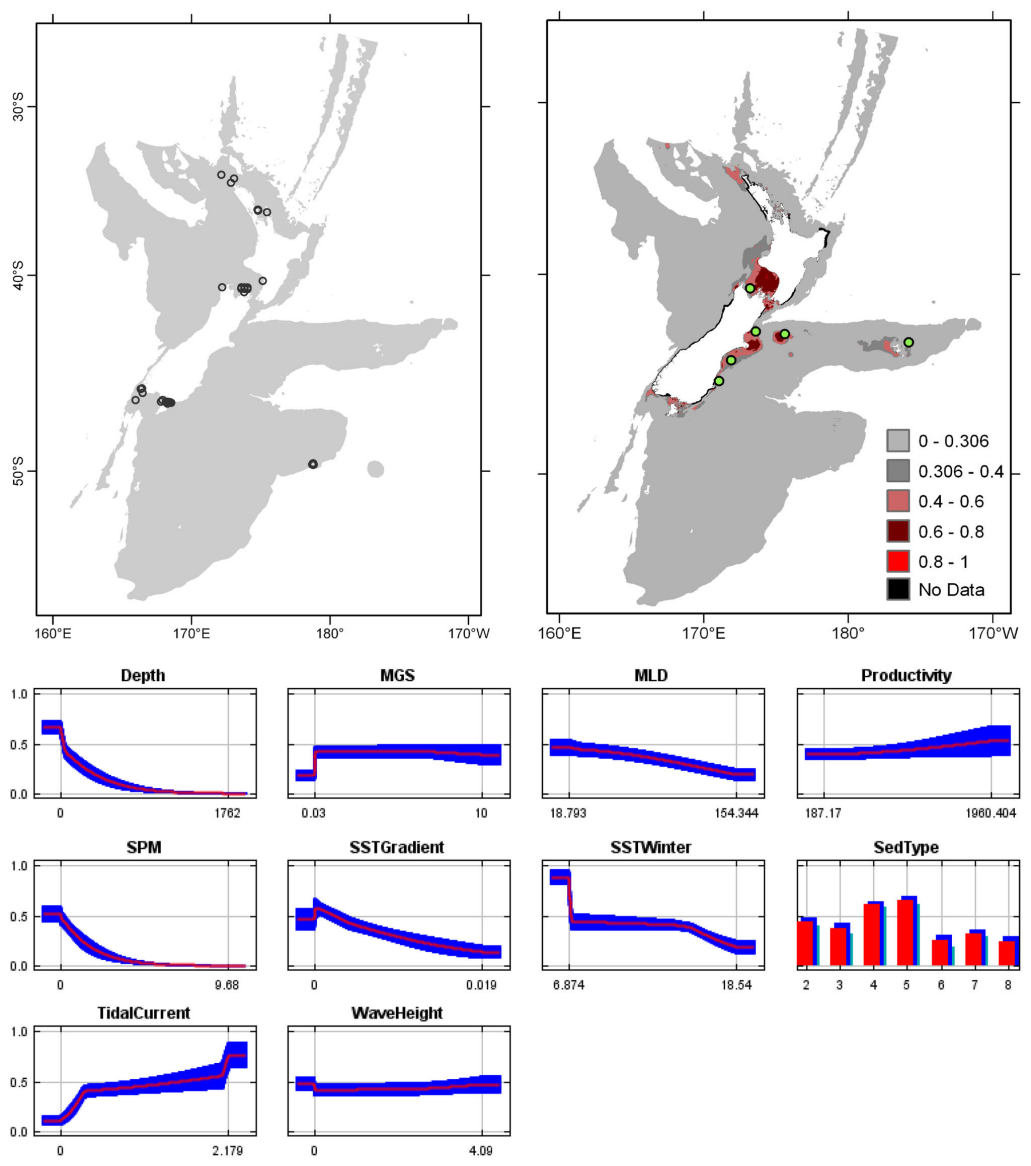
*Hippomenella* *vellicata* known distribution (left), predicted suitable habitat (right), and fitted responses curves (marginal). For the predicted distribution, logistic probabilities less than the 10^th^ percentile presence value indicated cells were unsuitable habitat. Probabilities of 0.4–0.6 indicated habitat suitability typical of the presence records, values of 0.6–0.8 and 0.8–1 indicated favourable and highly suitable habitat, respectively. Black cells had missing data in one or more environmental layer. Independent records are layed over the predictions. Marginal response curves show how the prediction changes for different values of each variable when all other variables were at their average sample value. Individual response curves are shown in Figure S3. For the categorical variable Sediment type: 1 = deep ocean clays; 2 = calcareous gravel; 3 = volcanic; 4 = calcareous mud; 5 = gravel; 6 = mud; 7 = sand; 8 = calcareous sand.

**Figure 12:**
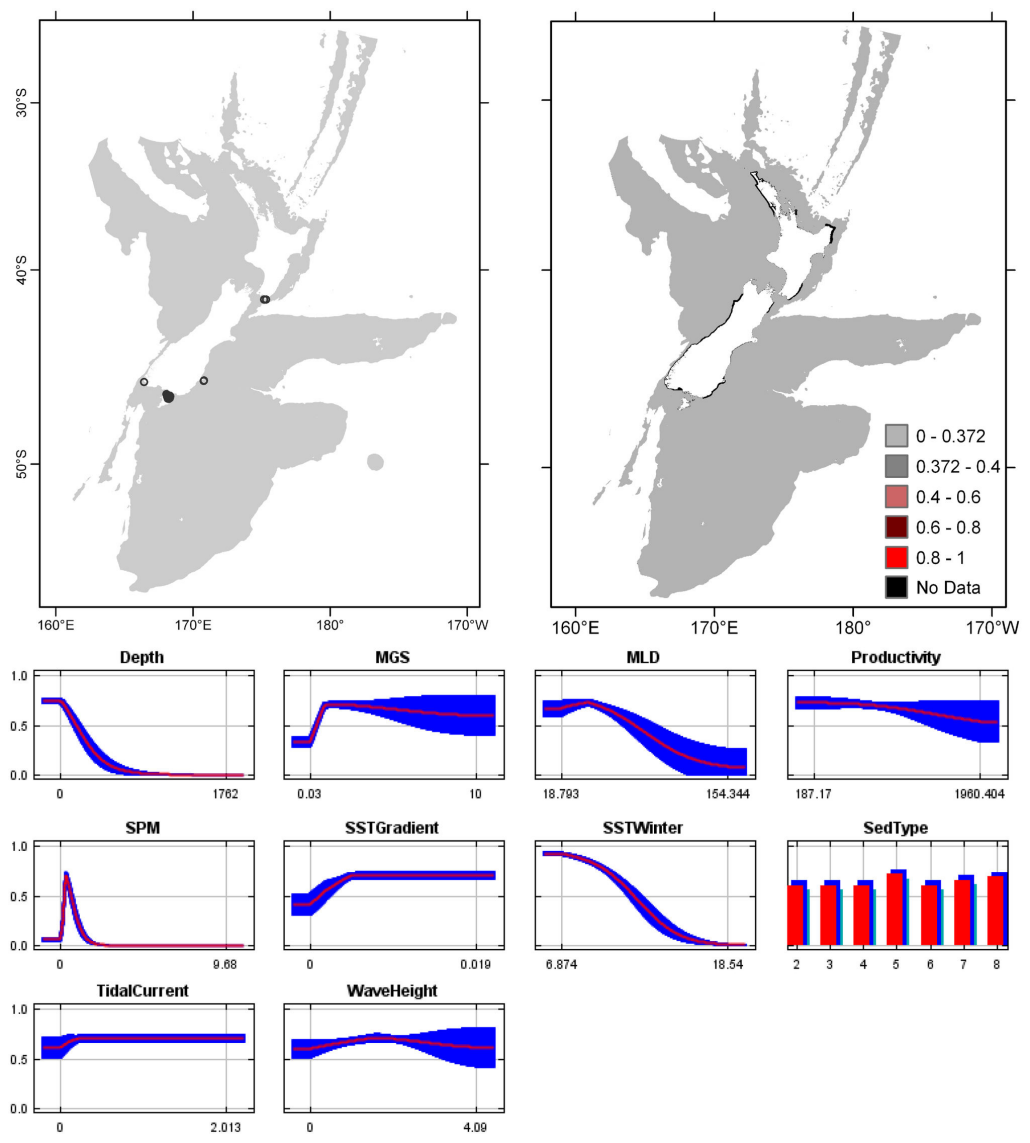
*Hornerafoliacea* known distribution (left), predicted suitable habitat (right), and fitted responses curves (marginal). For the predicted distribution, logistic probabilities less than the 10^th^ percentile presence value indicated cells were unsuitable habitat. Probabilities of 0.4–0.6 indicated habitat suitability typical of the presence records, values of 0.6–0.8 and 0.8–1 indicated favourable and highly suitable habitat, respectively. Black cells had missing data in one or more environmental layer. Independent records are layed over the predictions. Marginal response curves show how the prediction changes for different values of each variable when all other variables were at their average sample value. Individual response curves are shown in Figure S3. For the categorical variable Sediment type: 1 = deep ocean clays; 2 = calcareous gravel; 3 = volcanic; 4 = calcareous mud; 5 = gravel; 6 = mud; 7 = sand; 8 = calcareous sand.

**Figure 13:**
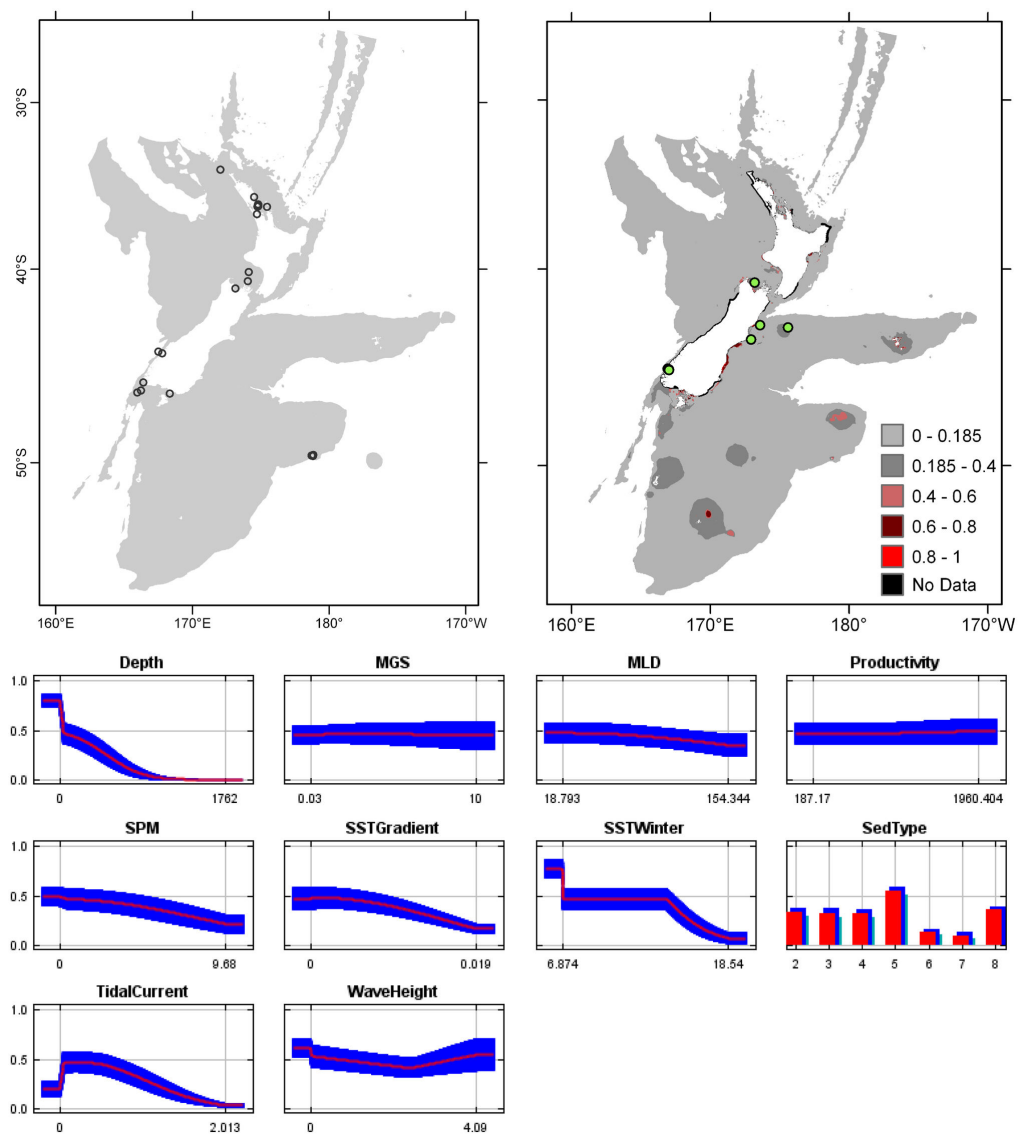
*Smittoideamaunganuiensis* known distribution (left), predicted suitable habitat (right), and fitted responses curves (marginal). For the predicted distribution, logistic probabilities less than the 10^th^ percentile presence value indicated cells were unsuitable habitat. Probabilities of 0.4–0.6 indicated habitat suitability typical of the presence records, values of 0.6–0.8 and 0.8–1 indicated favourable and highly suitable habitat, respectively. Black cells had missing data in one or more environmental layer. Independent records are layed over the predictions. Marginal response curves show how the prediction changes for different values of each variable when all other variables were at their average sample value. Individual response curves are shown in Figure S3. For the categorical variable Sediment type: 1 = deep ocean clays; 2 = calcareous gravel; 3 = volcanic; 4 = calcareous mud; 5 = gravel; 6 = mud; 7 = sand; 8 = calcareous sand.

The TGB data, which included the presence records of the study species, comprised about 650 sites in the depth range 0–2000 m. These sites were distributed mainly at shelf depths (84% <250 m, and 90% <500 m) but extended to ~1800 m (Figure 2). Latitudinally, sites ranged from 28°–53°S (Kermadec Ridge to east of Campbell Rise). Longitudinally, sites ranged from 165° E–177°W (south-western South Island to east of the Chatham Islands). Sites were concentrated mainly on Kermadec Ridge, around the northern Northland Peninsula/Manawatāwhi/Three Kings Islands area (northern North Island), Hauraki Gulf, South Taranaki Bight/Tasman and Golden Bay/Marlborough Sounds/Cook Strait (Greater Cook Strait), in Foveaux Strait, and from the coast of Fiordland to Puysegur ‘Bank’ (see Figure 1 for place names). Areas with good sample coverage included both coasts of the Northland Peninsula, North Island’s east coast, South Island’s west coast, Chatham Rise, and parts of The Snares, Auckland Islands, and Campbell Islands/Motu Ihupuku shelves. The presence records for each study species generally occurred in these same areas, although differences were apparent (Figures 3–13). For example, *Celleporariaagglutinans* was the only species recorded around eastern North Island, and *Celleporinagrandis* was the only species recorded from the Chatham Rise. Presence records of some species occurred New Zealand-wide (e.g. *Arachnopusia* *unicornis* ) and others were confined to certain latitudes (e.g. *Cinctipora* *elegans* ).

Detailed descriptions of model validation and predicted distribution for each species are given in Text S1, and high-resolution predictive maps are given for each species in Figures S4–S14. Habitat suitability models were successfully generated for 8 of the 11 species, based on AUC values (Table 2), and on jackknife charts for training gain, testing gain, and AUC values (Figure S3). These were for *Cellaria* *immersa* , *Celleporariaagglutinans* , *Celleporinagrandis* , *Cinctipora* *elegans* , *Diaperoecia* *purpurascens* , *Galeopsis* *porcellanicus* , *Hippomenella* *vellicata* , and *Smittoideamaunganuiensis* . The models for *Cellaria* *immersa* , *Celleporariaagglutinans* , *Diaperoecia* *purpurascens* , and *Smittoideamaunganuiensis* also had low omission rates, but the omission rates for *Celleporinagrandis* , *Cinctipora* *elegans* , *Galeopsis* *porcellanicus* , and *Hippomenella* *vellicata* were relatively high (>15%). Both model omission and AUC values showed the models for *Arachnopusia* *unicornis* and *Cellaria* *tenuirostris* were insufficiently accurate. The model for *Hornerafoliacea* had high validation statistics but unusual response curves which resulted in no cells being predicted as suitable. The marginal response curves for *Hornerafoliacea* indicated a generalist species but the individual response curves indicated this species had very narrow habitat requirements, visible in the box-and-whisker plots (Figure S2). Presence records of *Hornerafoliacea* had the narrowest range of environmental variable values of all the species modelled. In consequence, the model was excluded from further analysis, as were those of *Arachnopusia* *unicornis* and *Cellaria* *tenuirostris* .

**Table 2:** The number of presence samples (n) for each habitat-forming bryozoan species (duplicates in 1 km^2^ cells removed), and the mean (10-fold cross-validation) model validation statistics.

	***n***	**Test AUC mean (sd**)	**Test gain mean (range**)	**10^th^ percentile training presence**	**Test omission rate as % (Fixed threshold 10**)
*Arachnopusia* *unicornis*	89	0.669 (0.076)	0.064 (-0.352–0.488)	0.350	20.3
*Cellaria* *immersa*	50	0.827 (0.061)	0.733 (-0.430–1.480)	0.305	10.5
*Cellaria* *tenuirostris*	53	0.668 (0.101)	0.065 (-0.452–0.798)	0.365	13.0
*Celleporariaagglutinans* E	57	0.770 (0.072)	0.459 (-0.820–1.037)	0.33	12.7
*Celleporinagrandis* E	22	0.780 (0.082)	0.423 (-0.904–1.392)	0.401	20.0
*Cinctipora* *elegans* E	50	0.825 (0.070)	0.778 (0.042–1.251)	0.391	25.0
*Diaperoecia* *purpurascens* E	51	0.765 (0.071)	0.312 (-0.947–1.041)	0.309	14.0
*Galeopsis* *porcellanicus* E	42	0.767 (0.087)	0.390 (-0.399–1.128)	0.279	17.5
*Hippomenella* *vellicata* E	63	0.707 (0.093)	0.221 (-1.062–0.800)	0.306	18.3
*Hornerafoliacea*	20	0.881 (0.044)	1.239 (-0.970–1.990)	0.372	10.0
*Smittoideamaunganuiensis*	27	0.799 (0.082)	0.804 (-0.955–2.501)	0.185	11.7

E = endemic species.

The highest Pearson correlations between environmental variables occurred between productivity and SPM (0.46) for presence records, and between productivity and sediment type (0.52) for TGB data (worksheet Variable correlations in Table S2). These correlations were sufficiently low that multicollinearity amongst environmental data was not further considered. Response curves showed the fitted responses between the bryozoans and the ten broad-scale environmental variables (Figures 3–13 and Figure S3). All of the variables except for MGS were important (>10%) to the permuted model of one or more species; MGS contributed 8.4% to the permuted *Cellaria* *immersa* model (Table 3). Variables with important roles in multiple models were depth, mixed layer depth, SST in winter and tidal current. Overall, the response curves indicated the habitat-forming bryozoan species were associated with shelf depths, relatively shallow mixed layer depths, and intermediate to high current speeds, although there were exceptions, for example *Celleporinagrandis* was predicted to find suitable habitat at all depths. SST in winter indicated suitable habitat for most species occurred in the temperate waters around North and South Islands, but the effect of the Subtropical Front, which extends east across the Chatham Rise, meant suitable habitat also occurred at the Chatham and Bounty Islands. Shallow areas further south (e.g. at the Antipodes Islands) were 2–4°C colder than mainland areas at the same latitude (SSTWinter in Figure S1), and were predicted as unsuitable for most species (Figures 3–13). Again, there were species-specific responses, for example the response curves for *Cinctipora* *elegans* indicated suitable habitat had a maximum SST in winter of 12°C, meaning this species would be restricted to areas south of the lower North Island.

**Table 3:** Mean (10-fold cross-validation) variable permutation importance to Maxent models.

	**Depth**	**MGS**	**MLD**	**Prod**	**SPM**	**SSTGrad**	**SSTWinter**	**SedType**	**TidalCurr**	**WaveHeight**
*Arachnopusia* *unicornis*	25.3	4.0	14.2	1.0	5.1	2.8	16.2	9.7	14.0	7.6
*Cellaria* *immersa*	10.4	8.4	48.1	1.0	1.7	1.3	6.2	0.5	5.7	16.7
*Cellaria* *tenuirostris*	17.0	0.4	26.1	6.5	0.0	3.8	17.8	6.5	2.8	19.2
*Celleporariaagglutinans*	27.2	2.2	0.9	12.3	17.6	1.4	6.2	2.5	20.6	9.2
*Celleporinagrandis*	1.1	0.2	22.5	3.7	0.4	18.6	35.7	5.0	6.2	6.5
*Cinctipora* *elegans*	3.7	0.5	22.7	2.5	1.4	0.3	32.6	3.8	26.7	6.0
*Diaperoecia* *purpurascens*	8.3	1.4	22.3	6.1	3.3	2.0	14.9	2.1	37.5	2.1
*Galeopsis* *porcellanicus*	30.1	2.9	1.4	0.8	2.1	11.2	18.2	16.5	16.1	0.6
*Hippomenella* *vellicata*	20.7	2.8	16.4	10.0	0.7	2.8	4.0	16.4	23.4	2.8
*Hornerafoliacea*	26.1	2.4	0.3	2.0	17.2	9.9	37.1	1.6	2.8	0.4
*Smittoideamaunganuiensis*	22.8	1.0	0.9	0.5	0.5	1.2	26.3	35.0	7.1	4.9

Depth = water depth; MGS = median grain size; MLD = mixed layer depth; Prod = surface water primary productivity; SPM = total suspended particulate matter concentration; SSTGrad = sea surface temperature gradient; SSTWinter = sea surface temperature in winter; TidalCurr = depth averaged maximum tidal current; WaveHeight = annual average wave height.

Overall, areas known to have bryozoans and for which suitable habitat was predicted were northern North Island, outer Hauraki Gulf, Greater Cook Strait, Fiordland, Puysegur ‘Bank’, Foveaux Strait, and Otago shelf (Figures 3–13). Areas where there were no presence records in the collated data, but where suitable habitat was predicted included the outer-shelf west of Taranaki, the shelf just south of Stewart Island, much of the Canterbury Bight, around Banks Peninsula, Mernoo Bank, and around the Chatham and Bounty Islands. Areas where collated records occurred but where suitable habitat was not predicted were Kermadec Ridge and the Antipodes Islands, i.e. towards the edges of the study area. The east coast of North Island was predicted as largely unsuitable. Similarly, much of the west coast of South Island was predicted as unsuitable for all but *Celleporinagrandis* and *Cellaria* *immersa* .

**Figure 14:**
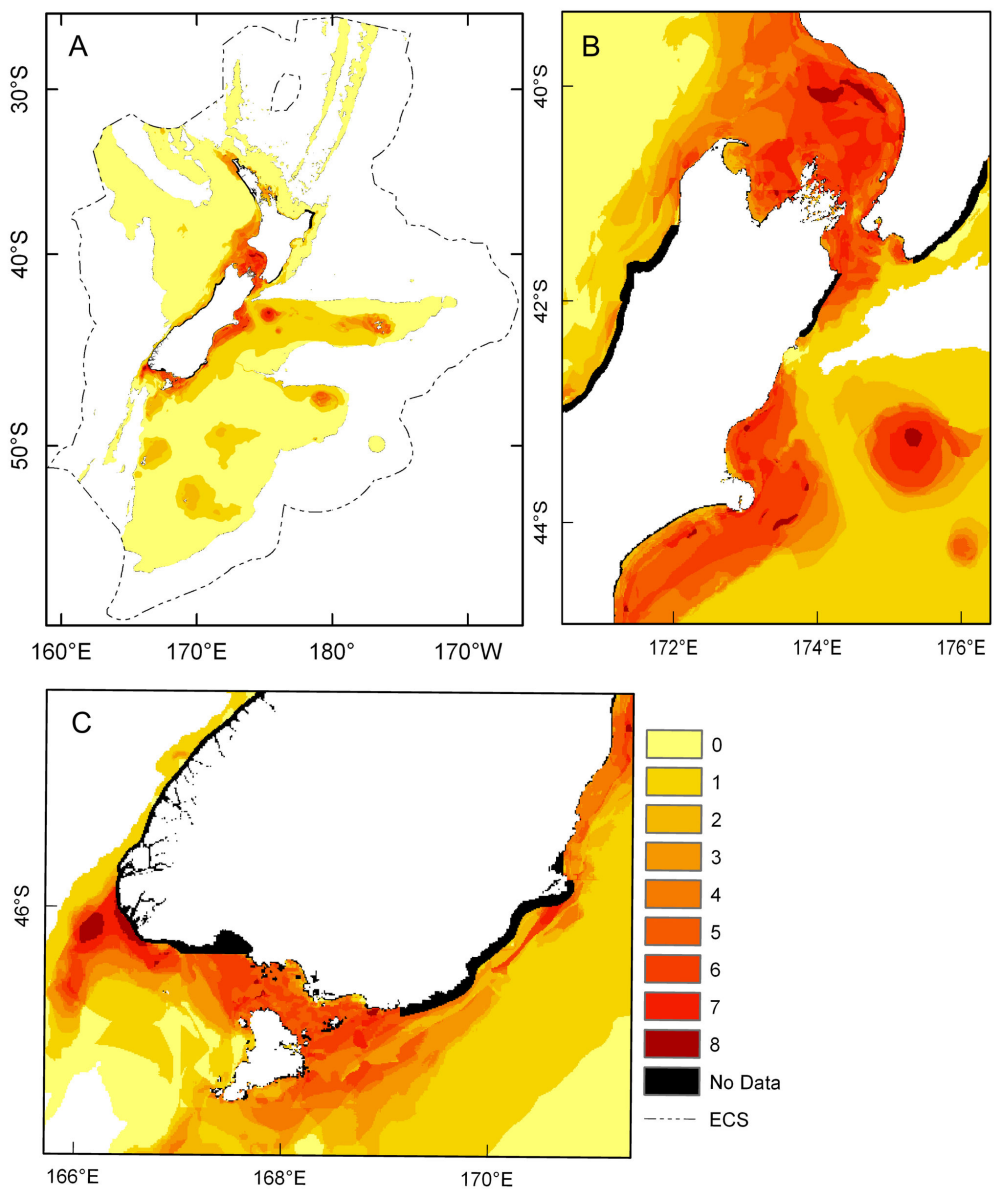
Predicted hotspots of habitat-forming bryozoans. Summed binary predictions of suitable habitat for multiple bryozoan species identified using 10^th^ percentile training presence threshold. Predictions were summed for *Cellaria* *immersa* *,* *Celleporariaagglutinans* *,* *Celleporinagrandis* *,* *Cinctipora* *elegans* *,* *Diaperoecia* *purpurascens* *,* *Galeopsis* *porcellanicus* *,* *Hippomenella* *vellicata* and *Smittoideamaunganuiensis* and are shown for A) the Extended Continental Shelf; B) Greater Cook Strait, Banks Peninsula and Mernoo Bank; and C) around southern South Island, including Puysegur ‘Bank’, Foveaux Strait and Otago shelf. 0-8 = the number of species predicted to find suitable habitat. Cells for which data in one or more environmental layer were missing are black.

Summed binary outputs indicated bryozoan ‘hotspots’ (Figure 14) occurred both where there were many presence records (northern North Island, Hauraki Gulf, Greater Cook Strait, Puysegur ‘Bank’, Foveaux Strait, Otago shelf), where there were few (Canterbury Bight, Chatham Islands), and where there were none (west of Taranaki, Banks Peninsula, Mernoo Bank, Bounty Island).

## Discussion

The presence records collated here for 11 species represent more than a century of sampling of bryozoans in New Zealand waters. The present work mirrors recent studies which have incorporated modelling methods to describe the known and potential distributions of cold-water corals, habitat-forming species from deeper water which have experienced fishing-related damage, with the aim of providing information to support the management of these taxa [33,34,35]. With a view to supporting such management at shelf depths, the distribution of ‘hotspots’ of habitat suitable for these species has been compared (below) with the distribution of closed areas and with available summaries describing broad-scale fishing effort. First, however, we consider the main limitations of Maxent models, particularly those based on historic data and using broad-scale environmental variables.

### Model considerations

Recommended techniques for Maxent modelling were applied to avoid overfitting predictions (i.e. minimum sample number [36]), in addition to the default settings which were developed to maximise model quality [37]. Using TGB instead of random background data addressed sample bias. TGB data can improve model performance [38], but can also decrease AUC by 10–15% [65]. Two models had AUC values <0.7, suggested as the lower limit for ‘useful’ models [62]. Six models had omission rates higher than 10% [60], meaning that during testing, these models did not successfully identify all recorded observations of the study species. Maxent’s failure to generate accurate models for *Arachnopusia* *unicornis* and *Cellaria* *tenuirostris* (low AUC values) may reflect its tendency to perform best when the modelled species has very specific habitat requirements [63,66]. These were the most widely distributed of the study species (both ranging from about 30°10′S to 49°40′S), which may indicate their more general habitat requirements. In contrast, the habitat requirements for *Hornerafoliacea* were so narrow (perhaps resulting from the comparatively small number of presence records on which the models were based) that no cells met the criteria of suitable habitat, despite having relatively high AUC values. Analyses that are restricted by non-ecological boundaries (e.g. political boundaries like the New Zealand ECS) are likely to under-represent the true distribution of the species’ full range [67], whilst at the same time, AUC values may increase when the area of suitable habitat is small in relation to the total study area [64]. Such limitations may have affected the model for *Hornerafoliacea* , which is known from south-eastern Australia at 36° S [68], and other non-endemic species ( *Arachnopusia* *unicornis* , *Cellaria* *tenuirostris* , *Cellaria* *immersa* and *Smittoideamaunganuiensis* ).

All of the models could be improved by further sampling, by adding to the presence points collated here, and by refining some of the more important environmental layers, but comparisons with independent data (below) promote confidence that the majority of the models offer guidance for locating areas where the study species are likely to find suitable habitat, within the study area.

One of the aims of the present study was to find the potential distribution of habitat suitable for the study species in the absence of anthropogenic impacts. The use of historic data was essential then, since anthropogenic impacts such as fishing have increased in areal coverage and intensity over time. However, using historic data brings with it some limitations. About half of the presence records were collected before 1967, and so had a potential for spatial error of up to 7.5 km (Table S1). In addition, 26 presence records were located using Google Maps and these records have an estimated spatial error of 0.3–7 km (Table S1). Spatial error could also be introduced by the use of records obtained from sampling using mobile gear (i.e. non-point data). About 45% of all presence records were collected with mobile gear such as dredges and trawls. The co-ordinates used for locating presence records in the study area were from the start of the tow, but depending on the duration and direction of the tow, the sample itself could have been collected from some distance away, potentially in another cell. Of 240 samples collected with mobile gear, however, 235 were collected with gear usually towed for short distances (e.g dredges towed for <200 m). Due to the short duration of such tows, end co-ordinates were not always recorded, particularly for samples taken before 1960. Five samples were collected by fish trawl (Table S1), which can be towed over many hundreds of metres or kilometres, and there is a reasonable probability that these samples have introduced a degree of uncertainty in the models generated. However, because trawl-based records accounted for such a small proportion of the total data used, the level of uncertainty introduced is considered to be very low. Generating models at coarser resolutions (e.g. 15 km^2^) would account for these spatial errors, but would cause other problems, including the potential for overprediction [69], the loss of data points and consequently the loss of study species following data removal of duplicate records from cells.

Maxent was developed with the aim of enabling the use of collections held by, for example, natural history museums [31], and subsequent research has shown this is achievable, but that there are limitations depending on the quality of the presence records. Testing of model accuracy assessed using AUC [70] found that of a number of methods trialled, Maxent was one of the methods most robust to spatial inaccuracies, and that AUC decreased with higher levels of spatial error. Further research [71] found an average decrease in AUC values of 10% for presence-point shifts of 4–5 grid cells, and showed that gain also decreased, and showed greater sensitivity to spatial error than did AUC. Changes to predictions based on less-accurate data were not always apparent, so that in general, core areas of suitable habitat were recognised by the models with spatial error, although peripheral detail varied. Osborne and Leitão [71] considered that, if only core areas of suitable habitat were used, models based on inaccurate data would be unlikely to result in management errors, but considering peripheral areas would be likely to do so. Similarly, Graham et al. [70] noted, ‘the key here is not to over-interpret the results of these models. They are useful at certain spatial scales, and should not be used at finer scales where they may be less useful.’

In the present study, accepting the limitations imposed by spatial error resulting from the use of historic records requires the predictions to be interpreted broadly, in relation to the 1 km^2^ grid size used. One of the smaller areas interpreted as indicating suitable habitat, the central area of Mernoo Bank (the small, circular red feature at 175°19′E in Figure 14B) has an area of ~300 km^2^, and Foveaux Strait (Figure 14C, the area suitable for 5 or more species to the south of South Island), has an area of >6000 km^2^. The limitations also require interpretation to focus on core habitat areas, achieved here both through interpreting only large areas of suitable habitat for each species, and through the use of ‘hotspots’ which identified core habitat for multiple species (see below). In terms of the application of the models generated here, their comparison to the fishing data (below) is appropriate because the fishing data are even more broad-scale than the potential spatial error in the models.

Another aim of the present study was to identify the broad-scale environmental conditions underlying the predicted distributions of these bryozoans. Osborne and Leitão [71] showed that whilst the predictions generated by less accurate data were largely reliable, the relative importance of the environmental variables could change unpredictably, meaning ecological interpretation of individual models was inappropriate. Despite our initial aim, we have made only broad generalisations from multiple species models with this limitation in mind.

Properties of the environmental layers also limit the quality of the models. The variables dominant sediment class and median grain size only describe soft sediments (Figure S1), but the 11 bryozoan species occur both on rocky substrata and stable soft sediments, and records used in the present study represent samples from both hard and soft substrata. In addition, many of the environmental layers do not extend up to the coastline (black cells in the prediction maps) where rocky substrata are most likely. These limitations mean areas of suitable habitat on rocky substrata and inlet/fiord environments will have been underestimated. This under-estimation of suitable habitat is relevant to areas such as Fiordland and other marine inlets around the country (see, for example, 72,73). A secondary limitation of the layers describing sediment variables are the clear boundaries between areas of differing sediment type, particularly those that result from differences in resolution between contiguous/nested charts that were combined to make the layers. Close inspection of the habitat predictions reveals their patchy nature can result from differences in median grain size and/or dominant sediment type (e.g. in Foveaux Strait and off Banks Peninsula). In reality, boundaries between patches of differing habitat suitability are unlikely to be defined as clearly as predicted here. Moreover, the majority of environmental layers used in the present study are too broad to allow identification of species’ niches, even if the available presence records were very much more accurate. As such, improving these habitat suitability models requires both more accurate presence/TGB records, and finer-scale environmental layers with which to generate models.

Disparities between the scales at which layers are generated and the scale at which they are ecologically relevant are a general limitation of habitat suitability models. Here, for example, SST in winter appears to be useful in describing the distribution of some of the study species at scales of hundreds of kilometres, but at smaller scales this variable is unlikely to be important. Similarly, using a 1 km^2^ grid instead of working at a coarser resolution allowed detail in the finer scale layers (e.g. depth) to be retained. In terms of model interpretation, the native resolution of all of the variables (Table 1) should be borne in mind. Future interpretation of these models must consider all of these limitations in relation to the present study, and also that whilst habitat suitability models of these species have potential for use beyond the present study, the next step in model development should be validation and refinement using independent data, improved environmental variables and species-specific model tuning.

### Known and predicted distributions

The distribution of the records collated here are shown for each species (Figures 3–13), and together with secondary records (superimposed over the prediction maps, also in Figures 3–13, and not included as presence records because they were not identified by D. Gordon), provide a comprehensive account of the known distribution of 11 habitat-forming bryozoan species in the New Zealand ECS. Here, we compare the known and predicted distributions in order to identify areas where suitable habitat is likely to occur, but from which these species have not been recorded. Where secondary data were available, they have been used for informal independent assessment to help identify limitations of the predictions. The predictions for *Arachnopusia* *unicornis* , *Cellaria* *tenuirostris* and *Hornerafoliacea* are not considered (see Results).

Predictions of suitable habitat for *Cellaria* *immersa* were verified by independent records at Mernoo Bank [74] and Separation Point [75], but Maxent failed to predict suitable habitat for *Cellaria* *immersa* on Otago shelf [13] and around Chatham Island [74], from where it has previously been recorded. Suitable areas for the endemic species *Celleporariaagglutinans* were predicted in the absence of presence points and verified by independent records [74] on Mernoo Bank and around Chatham Island, but Maxent failed to predict suitable habitat for this species at Separation Point, from where it has previously been recorded [75]. No independent records were found for *Celleporinagrandis* , also endemic. The northerly limit of the endemic species *Cinctipora* *elegans* (39–40° S) is well-established [39,76], and whilst presence records collated here occurred south only as far as ~47° S, there are observations for this species from 53° S (Campbell Plateau) [39]. Predictions of suitable habitat in the absence of collated records were verified by independent records [39] at Banks Peninsula, Mernoo Bank, the Chatham and Bounty Islands, and The Snares shelf. Maxent failed to predict suitable habitat for this species at the Campbell Islands/Motu Ihupuku, the Antipodes and Auckland Islands, on the Campbell and Pukaki Rises and on the southern Snares shelf, where it has previously been recorded [39]. The 179 records collated by Taylor et al. [39] occurred over 12–914 m water depth, the shallowest of which was from Fiordland. Only 12 of the records were from depths >250 m. The deepest records are considered to represent fragments transported after death from shallower water, rather than colonies living far beyond the shelf break [39]. The lack of suitable habitat predicted at the subantarctic islands probably resulted from the cooler seawater temperatures in these areas, which are outside the range shown as suitable on the response curves (see Figure S3, Text S1 and SSTWinter in Figure S1). The independent records confirm the suitability of these areas however, and indicate a limitation of the model predictions for *Cinctipora* *elegans* . Predictions of suitable habitat in the absence of collated records for *Diaperoecia* *purpurascens* (endemic) were verified by independent records around Banks Peninsula, on Otago shelf, Mernoo Bank, and around the Chatham and Bounty Islands [13,39,77,78,79]. Maxent failed to predict suitable habitat for this species on the southern Snares shelf, around the Campbell Islands/Motu Ihupuku, Auckland and Antipodes Islands, at the Campbell and Pukaki Rises, off East Cape and in the western Bay of Plenty, where it has previously been recorded [39]. Similar to those for *Cinctipora* *elegans* , records collated by Taylor et al. [39] ranged from 33° S to 54° S (outside the ECS but part of New Zealand’s shelf system), and from depths of 0–1156 m. Only 10% of the 137 records were deeper than 250 m, showing this species also occurs mainly at shelf depths. As for *Cinctipora* *elegans* , the lack of suitable habitat predicted for *Diaperoecia* *purpurascens* at the subantarctic islands may have resulted from the cooler seawater temperatures in these areas, which are outside the range shown as suitable on the response curves (Figures 9 and S3, see also SSTWinter in Figure S1). The independent records confirm the suitability of these areas, and again indicate a limitation of the model predictions for this species. Predictions of suitable habitat for *Galeopsis* *porcellanicus* (also endemic) were verified by independent records at Separation Point [75] and north-east of Chatham Island, but Maxent failed to predict suitable habitat for this species at Mernoo Bank from where it has previously been recorded [74]. Predictions of suitable habitat for *Hippomenella* *vellicata* (endemic) were verified by independent records at Separation Point [75], along the east coast of South Island [77,79], and on Mernoo Bank [74], but Maxent failed to predict suitable habitat east of the Chatham Islands [74]. Predictions of suitable habitat for *Smittoideamaunganuiensis* were verified by independent records at Akaroa Harbour (Banks Peninsula) [80], Separation Point [75] and to a lesser extent on Mernoo Bank [74]. Maxent failed to predict suitable habitat for this species north of Banks Peninsula and on Chatham Rise (secondary data not shown), from where it has previously been recorded [79,80]. *Smittoideamaunganuiensis* is known from Fiordland [81] where the predictions were prevented by the areal coverage of the variables (see Model considerations, above) and from southern Australia [80].

Overall, the models for *Cellaria* *immersa* , *Celleporariaagglutinans* , and *Smittoideamaunganuiensis* had relatively low omission rates, high AUC values, and predictions of suitable habitat for these species compared well to independent data. The prediction of suitable habitat for *Hippomenella* *vellicata* also compared well to independent data, but the model for this species had a high rate of omission and a relatively low (although >0.7 cut-off) AUC value. The predictions of suitable habitat for *Cinctipora* *elegans* and *Diaperoecia* *purpurascens* compared very well to independent data within the latitudes for which there were presence records, but failed to identify suitable habitat further south in areas shown as suitable by independent records. Models for these two species in particular may benefit from additional data points throughout their known ranges.

### Environmental suitability

Having noted the limitations on ecological interpretation of Maxent models generated using historical data (Model considerations, above), we briefly consider broad similarities and differences across models. The variables that had important roles in multiple models were depth, mixed layer depth, SST in winter and tidal current. Depth, temperature and current were among those variables identified as important by a global review of bryozoan-generated habitat, which found habitat-forming bryozoans were associated with continental shelf depths and areas of strong currents (e.g. in channels and around headlands) [4]. The suggested association between habitat-forming bryozoans and areas of high productivity [14] is only apparent in the importance of the SST gradient variable for *Celleporinagrandis* , for which habitat was predicted as occurring under the Subtropical Front (compare Figure 7 and SSTGradient in Figure S1), and less so for *Galeopsis* *porcellanicus* , for which the importance of the variable is much less clear (see 82 for discussion of the effects of the Subtropical Front on benthic assemblages).

The bryozoans’ response curves for both depth and mixed layer depth indicate suitable habitat for all species being at shelf depths (<250 m). Deeper than 250 m, very small areas at the edge of large, shallower suitable areas, were predicted as suitable, for example west of Manawatāwhi/Three Kings Islands ( *Cellaria* *immersa* ). Habitat-forming bryozoans are known from beyond the shelf break, but in lower abundance than on the mid- to outer-shelf (e.g. off the Otago shelf [13]). Only for *Celleporinagrandis* did significant areas of suitable habitat occur beyond the shelf break, and in some places (e.g. north of Chatham Rise), suitable habitat for this species was predicted up to the 2000 m depth limit of the model. Mixed layer depth also describes an aspect of food availability. When the mixed layer depth is deep, a smaller proportion of food generated in the surface waters reaches the benthos [52]. All the bryozoans were predicted to find their most suitable habitat in areas where the annual mean mixed layer depth was <50 m, where the supply of labile particulates to the benthos was likely to be proportionally high. Other measures of food availability (surface water productivity and SST gradient) were less important to the models, and it may be that the kilometre scale of these variables was not relevant to the scale at which bryozoans experience food availability. SST in winter described well the latitudinal distribution of the bryozoans. Some species occurred across a wide range of SST in winter values (e.g. *Hippomenella* *vellicata* ), whilst others (e.g. *Cinctipora* *elegans* ) had a clear latitudinal limit, likely related to some aspect of temperature control on their physiology. Depth-averaged maximum tidal current was elevated in all places where habitat was predicted as suitable, in channels such as Foveaux Strait, or where seafloor features (banks and rises) constricted water movement (e.g. Mernoo Bank). Moderately-moving water would benefit habitat-forming bryozoans by increasing the rate of food delivery while not being fast enough to destabilise the substratum or of sufficient velocity to carry large particles that could abrade the bryozoan colonies or interfere with feeding [83].

Stable substrata for attachment and low levels of sedimentation and disturbance so that feeding apparatus are not clogged [7] are properties of habitat associated with coarse, stable sediment. *Smittoideamaunganuiensis* , for example, was predicted to find suitable habitat where sediments were relatively coarse (and by inference more stable). Response curves for *Galeopsis* *porcellanicus* and *Hippomenella* *vellicata* indicated that finer dominant sediment types (calcareous mud) would be suitable, as would gravel. Although this predicted suitability contradicts the hypothesis that bryozoans require coarse, stable substrata, this may indicate that stability is more important than grain size to the study species, meaning the distribution of these taxa may be controlled by environmental variables different from those initially thought to be important. For example, cellariiform bryozoans such as *Cellaria* *immersa* are often associated with fine sediments, and develop rootlets to maintain their position [84], but are not found where sediments are mobile. On Otago shelf, for example, *Cellaria* *immersa* is absent from mobile sands in shallow waters (<60 m [13]) where wave action is high, but occurs on heterogeneous sediments slightly further offshore, where wave action is less. Similarly, *Celleporinagrandis* occurs in deeper water where even muddy sediments are likely to be relatively stable. At shelf depth, sediments described as muddy may actually comprise poorly-sorted sediments in which coarse gravels overlie muddier sediments (e.g. [85]). On the mid-shelf these are likely to be stable in all but the biggest storms [86]. Identifying the environmental characteristics of a location at which a study species occurs provides insight into its ecology, an insight which may allow us to identify with better accuracy the areas in which bryozoans generate habitat, and to understand the functional role of that habitat. Ecological interpretation of Maxent models based on higher quality data may offer such insight, and is a goal worthy of further study.

### Hotspots

Identifying areas where bryozoans may generate habitat is a first step towards their successful management. Within the study area, the predictive models have delimited areas where 8 species of bryozoan are most likely to find suitable habitat. These bryozoans are known to generate habitat for other species, but predicting where the bryozoans may occur is not the same as predicting the occurrence of bryozoan-generated habitat at metre–kilometre scales. Known areas where bryozoans dominate the seafloor in New Zealand, on their own or with other habitat-forming suspension-feeding invertebrates include: Spirits Bay (Piwhane Bay) off the northern North Island [12,87]; Separation Point [15,16,75], Torrent Bay [15,16,75,88], D’Urville Island ( [15,16], C. Duffy, DoC, pers comms 17/12/2007), Patten Passage (Guard’s Pass) and Fisherman’s Pass in French Pass (C. Duffy, DoC, pers comms 17/12/2007), all within Greater Cook Strait, but concentrated along the north coast of South Island; Otago shelf [13,77]; Foveaux Strait [89,90]; and Paterson Inlet at Stewart Island/Rakiura [72]. These areas broadly match the predicted distribution of suitable habitat for the individual habitat-forming species, but specific areas of known habitat-forming bryozoans, including *Celleporariaagglutinans* at Separation Point and *Hippomenella* *vellicata* at Torrent Bay, were not predicted by the models. Further, whether or not species are habitat-forming in areas predicted as suitable, such as Mernoo Bank and off Banks Peninsula, is yet to be determined. Constructing models that distinguish between habitat suitable for bryozoans that can form habitat, and areas where bryozoan-generated habitat occurs (e.g. [91]) was beyond the present study because few data exist for locations where habitat-forming bryozoans are known. In the absence of such data a second option is to identify areas where conditions are suitable for the highest number of species and to infer that in these places there is a higher likelihood of bryozoans generating habitat. This is realistic because habitat-forming bryozoans often dominate the benthos as multi-species assemblages [4,77]. Summed binary predictions of successfully modelled species (Figure 14) showed suitable areas for multiple species could be divided into two groups: areas suitable for 4–5 species, and hotspots suitable for all 8 species. Areas suitable for 4–5 species included those around northern North Island, Hauraki Gulf, west of Taranaki, North Taranaki Bight, and off the Chatham and Bounty Islands. Areas suitable for up to 8 species were Greater Cook Strait, off Banks Peninsula, Mernoo Bank, Puysegur ‘Bank’ and Foveaux Strait. These ‘hotspots’ may be used to guide future sampling to establish the distribution of bryozoan habitat in the ECS, and are used here to assess the potential interaction between bryozoan habitat and fishing.

### Interaction with fishing

**Figure 15:**
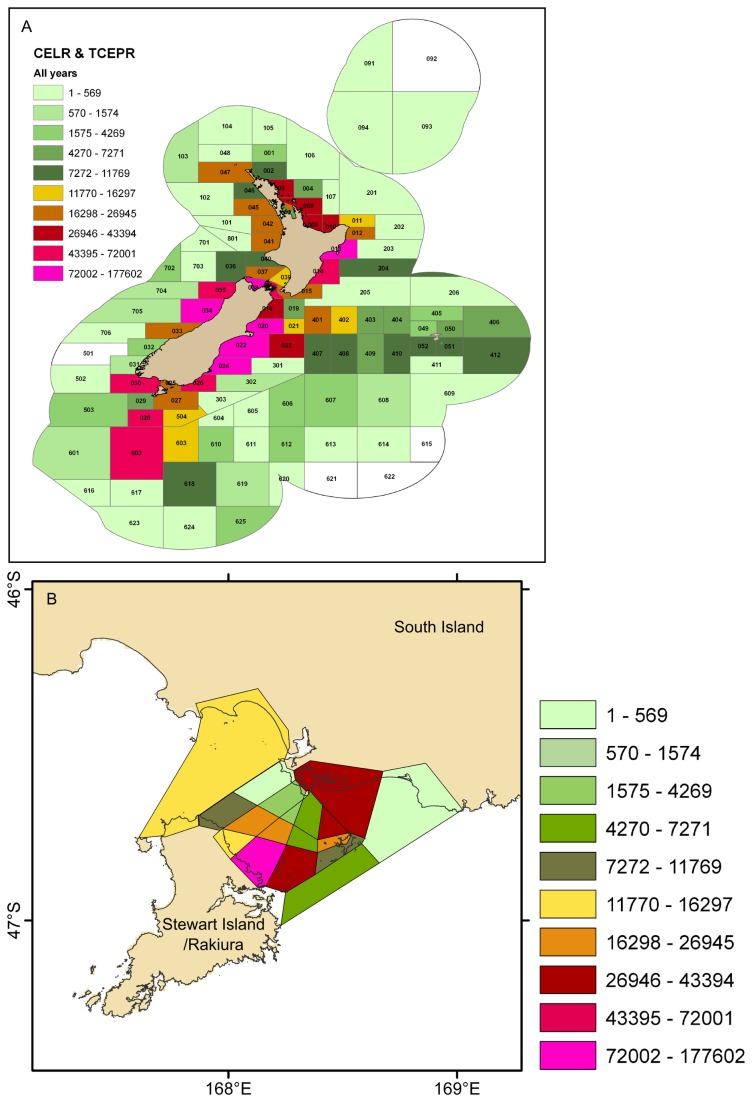
Bottom trawls and dredges for fishing years 1989–90 to 2004–05 inclusive. A) The distribution of bottom trawl effort in the New Zealand Exclusive Economic Zone, reproduced from Baird et al. [92] with permission from the Ministry for Primary Industries. B) The number of oyster dredge tows in Foveaux Strait (additional to trawls shown in A), by oyster statistical area, using data from Baird et al. [92]. An additional +2000 oyster dredge tows were reported from Foveaux Strait over the same period [92], but not by area, and are therefore not included here.

Raw data for fishing effort in the ECS were not made available for the present study. However, regional summaries of these data were available, allowing an assessment of the overlap between fishing effort and hotspots of bryozoan habitat. Recent mapping of trawling effort for the 15 years 1989–90 to 2004–05 [92] shows trawling in the New Zealand Exclusive Economic Zone (EEZ, which lies within the ECS) has been concentrated in certain areas (Figure 15A), some of which coincide with the bryozoan hotspots identified here. For example, the South Taranaki Bight (northern Greater Cook Strait) was predicted as suitable habitat for 4–8 species of habitat-forming bryozoan (Figure 14B). The Fisheries Statistical Areas (FSAs) that cover the South Taranaki Bight received between (in the east) 11,770–16,297 trawl events, and (in the west) 16,298–26,945 trawl events in the 15-year period (Figure 15A). These levels of fishing effort are relatively low compared to other shelf areas of the New Zealand EEZ where bryozoan-generated habitat is also likely, and pockets of habitat-forming bryozoans do exist in South Taranaki Bight (D. Gordon unpublished data), an area not only fished, but subject to oil/gas extraction (www.tagoil.com/taranaki-basin.asp), and the proposed extraction of ironsands (www.ttrl.co.nz). The second most extensive area of suitable habitat was predicted off Banks Peninsula, with much of the shelf suitable for 6 habitat-forming bryozoan species, and smaller areas there suitable for 8, meaning areas of habitat-forming bryozoans are likely to occur at this location (Figure 14B). Fishing effort here for the 15-year period has been high, at 72,002–177,602 trawl events for each relevant FSA (Figure 15A). The occasional collections of habitat-forming bryozoan species from this area [78,79] may therefore represent remnants of bryozoan-generated habitat that have not been recorded in the literature.

In addition to trawling, certain coastal areas are dredged for scallops and oysters. EEZ-wide, dredging comprises about 50% of fishing events. In Foveaux Strait, for example, suitable habitat was predicted for 5–8 species, and the relevant FSA received 16,298–26,945 trawl events in the 15-year period (Figure 15A) and 326,797 dredge events. Dredging was focussed on the central strait directly north and north-east of Stewart Island/Rakiura [92], where small patches are targeted until fishing becomes unprofitable [93], and dredge events are recorded in smaller statistical areas (Figure 15B). Parts of Foveaux Strait have been fished for more than 140 years [93], so whilst the predictive models show there is a high probability of the environment being suitable for multiple species of habitat-forming bryozoans, fishing effort there has been such that bryozoan-generated habitats are unlikely to persist to any great extent [22]. Dredging in the EEZ in the 15-year period totalled more than 2 million dredge events, focussed on the northern North Island shelf (465,052 events), around Coromandel Peninsula (271,060 events), the northern South Island shelf (836,697 events), Chatham Islands (10,965 events), and Foveaux Strait (326,797 events) [92]. The level of fishing in these areas, and on the shelf as a whole, indicates a conflict is likely between the conservation of habitat-forming bryozoans and their associated fauna, and the continuation of fishing without suitable spatial management. The models produced by the present study highlight the nature and extent of this conflict, but also provide information to support management measures designed to mitigate the impact of seafloor disturbance on bryozoan-generated habitat in the New Zealand ECS. For example, after further testing and validation, the predictive models could be used in spatial management tools such as Marxan (www.uq.edu.au/marxan), to help provide management solutions for multiple species [94].

**Figure 16:**
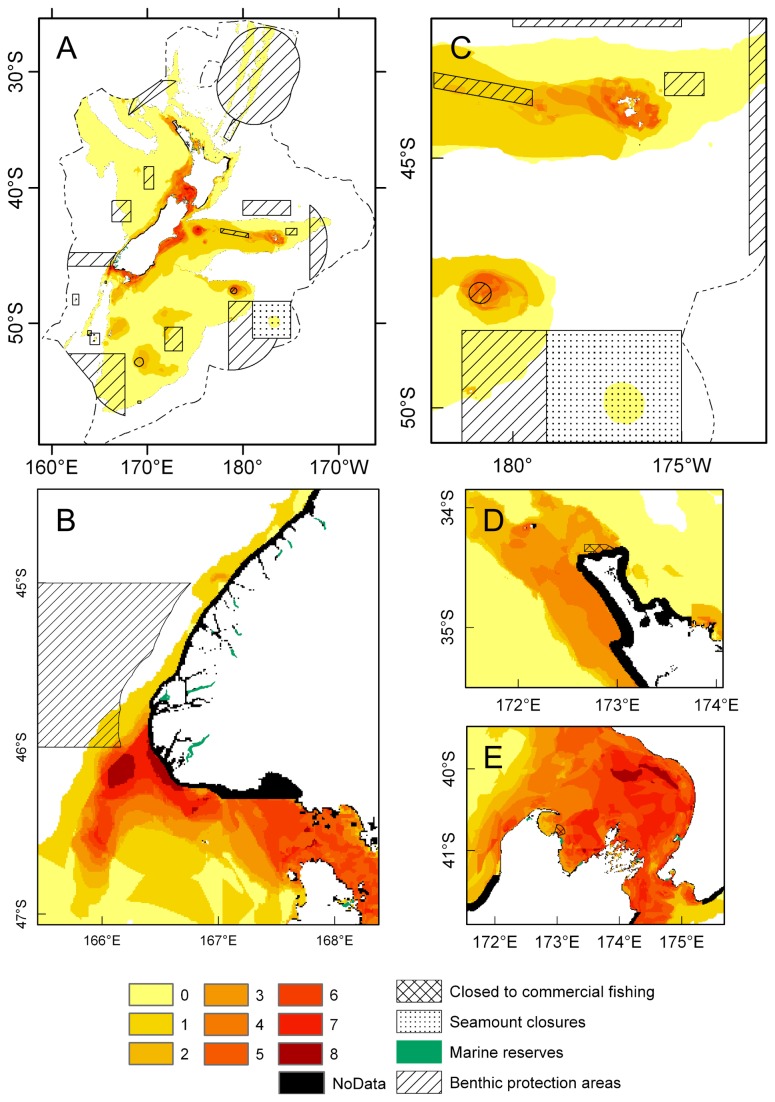
Protected seafloor in New Zealand. The spatial relationship between predicted bryozoan hotspots and areas closed to commercial fishing (no trawl, Danish seine or commercial dredge (amateur dredge allowed)), seamount closures, marine reserves/marine protected areas, and benthic protection areas in the New Zealand: A) across the Extended Continental Shelf; B) west of Fiordland (south-west South Island); C) on the eastern Chatham Rise, and around Chatham, Bounty and Antipodes Islands; D) off northern North Island; and E off northern South Island. Cells for which data in one or more environmental layer were missing are black.

Some management measures to protect benthic habitats of the New Zealand ECS are already in place (Figure 16). Areas closed to bottom trawling comprise 17% of the Territorial Sea and areas closed to all bottom fishing methods comprise 31% of the EEZ [95]. However, EEZ-wide closures are not in areas where bryozoan-generated habitat is either known or where suitable conditions for these species are predicted, with minor exceptions north-west of Puysegur ‘Bank’ (Figure 16B), at the eastern end of Chatham Rise, and around Bounty and Antipodes Islands (Figure 16C). Within the Territorial Sea, numerous, small, inshore areas are closed to various fishing methods [96,97], and the expansion of a network of marine protected areas is due (www.fish.govt.nz, accessed 24/07/12). At present, two areas closed to all commercial bottom-fishing methods purposefully coincide with areas of habitat-forming bryozoans: off northern North Island (Spirits Bay (Piwhane Bay)-Tom Bowling Bay, Figure 16D), and in Greater Cook Strait (Separation Point, Figure 16E) [96,97]. These areas are 224 km^2^ and 146 km^2^ respectively, though bryozoans (mainly *Celleporariaagglutinans* ) covered only 55 km^2^ at Separation Point in 2002, having covered 118 km^2^ in 1980, and about 213 km^2^ in 1945 [75]. An additional voluntary protected area intended to protect habitat-forming bryozoans exists on Otago shelf, but is neither listed [96,97] nor monitored.

Policies to mitigate the effects of fishing on bycatch species, to avoid or minimise adverse effects on biological diversity, and to avoid or minimise adverse effects of fishing activity on the benthic habitat (see the New Zealand Fisheries Act 1996) have not yet been implemented for shelf-depth habitats as they have for deeper areas (e.g. [98]). Given the patchiness of baseline data describing biogenic habitats in New Zealand waters, the present study highlights the need for reliable information on the historic and present-day distributions and ecological requirements of habitat-forming bryozoans, to support their future conservation as areas of significant biodiversity.

## Supporting Information

Figure S1 **Images of the environmental layers used to model habitat suitability for habitat-forming bryozoans: Depth = water depth; MGS = median grain size; MLD = mixed layer depth; Productivity = surface water primary productivity; SPM = total suspended particulate matter concentration; SSTGradient = sea surface temperature gradient; SSTWinter = sea surface temperature in winter; TidalCurrent = depth averaged maximum tidal current; WaveHeight = annual average wave height; Mask, to prevent predictions to water depths >2000 m; and SedType = dominant sediment class.**
(PDF)

Figure S2 **Box-and-whisker plots for each presence point of habitat-forming bryozoan species and for target-group background data, showing the distribution of values for each environmental layer.**
(TIF)

Figure S3 **Fitted response curves (individual) for each habitat-forming bryozoan species, showing how predicted habitat suitability changed with different values of each variable.** Together with the marginal response curves (Figures 3–13), these plots reflect the dependence of predicted suitability both on the selected variable and on dependencies induced by correlations between the selected variable and other variables. For the categorical variable sediment type: 1 = deep ocean clays; 2 = calcareous gravel; 3 = volcanic; 4 = calcareous mud; 5 = gravel; 6 = mud; 7 = sand; 8 = calcareous sand. Jackknife tests show the importance of each variable to the training and test gain, and to AUC.
(PDF)

Figure S4 **High resolution map of the predicted distribution of suitable habitat for *Arachnopusia* *unicornis* .**
(TIF)

Figure S5 **High resolution map of the predicted distribution of suitable habitat for *Cellaria* *immersa* .**
(TIF)

Figure S6 **High resolution map of the predicted distribution of suitable habitat for *Cellaria* *tenuirostris* .**
(TIF)

Figure S7 **High resolution map of the predicted distribution of suitable habitat for *Celleporariaagglutinans* .**
(TIF)

Figure S8 **High resolution map of the predicted distribution of suitable habitat for *Celleporinagrandis* .**
(TIF)

Figure S9 **High resolution map of the predicted distribution of suitable habitat for *Cinctipora* *elegans* .**
(TIF)

Figure S10 **High resolution map of the predicted distribution of suitable habitat for** Diaperoecia ***purpurascens*.**
(TIF)

Figure S11 **High resolution map of the predicted distribution of suitable habitat for *Galeopsis* *porcellanicus* .**
(TIF)

Figure S12 **High resolution map of the predicted distribution of suitable habitat for *Hippomenella* *vellicata* .**
(TIF)

Figure S13 **High resolution map of the predicted distribution of suitable habitat for *Hornerafoliacea* .**
(TIF)

Figure S14 **High resolution map of the predicted distribution of suitable habitat for *Smittoideamaunganuiensis* .**
(TIF)

Table S1 **Presence record collection details, including depths of habitat-forming bryozoans in New Zealand.** For mobile gear, co-ordinates were assigned at the beginning of the tow.
(XLS)

Table S2 **Excel file showing (1) presence record layer values (2), target-group background record layer values, and (3) Pearson correlations.**
(XLS)

Text S1 **Detailed descriptions of model validation, and the distributions of suitable habitat, for each species.**
(PDF)
