# Supplementary figures and images for: Habitat-Forming Bryozoans in New Zealand: Their Known and Predicted Distribution in Relation to Broad-Scale Environmental Variables and Fishing Effort
Source: PLoS One. 2013 Sep 23;8(9):e75160. doi: 10.1371/journal.pone.0075160 (PMC3781067; doi:10.1371/journal.pone.0075160)

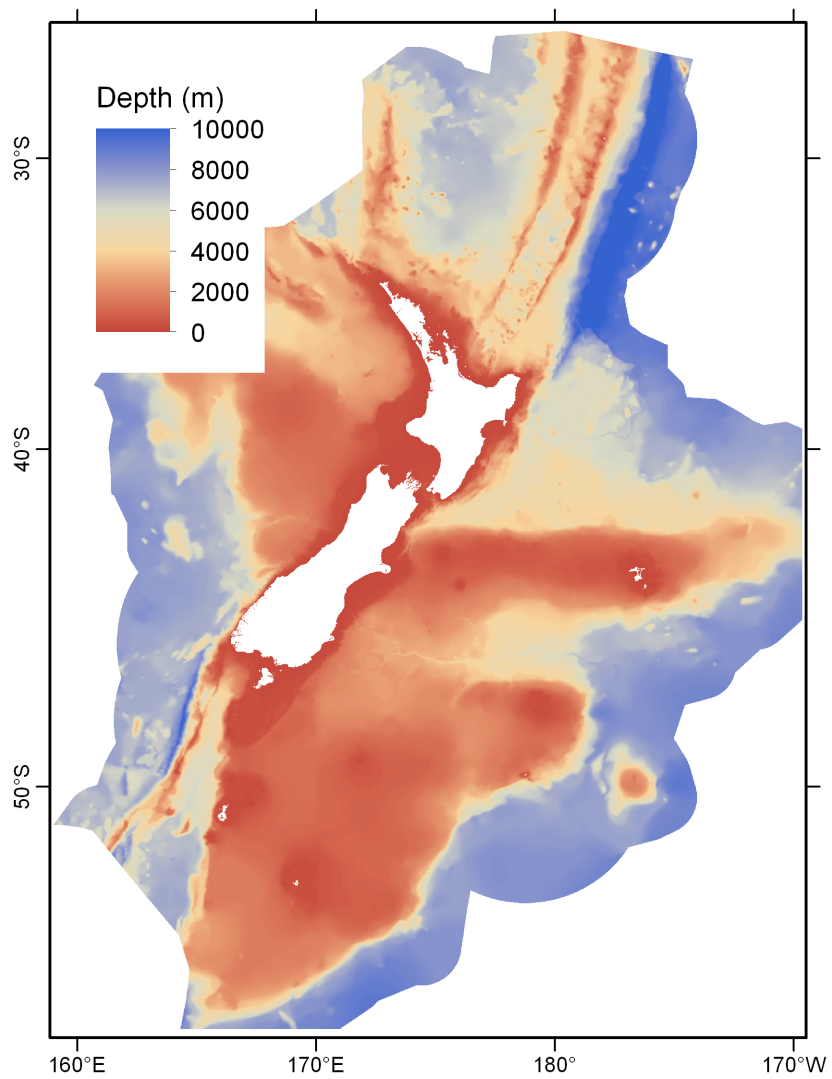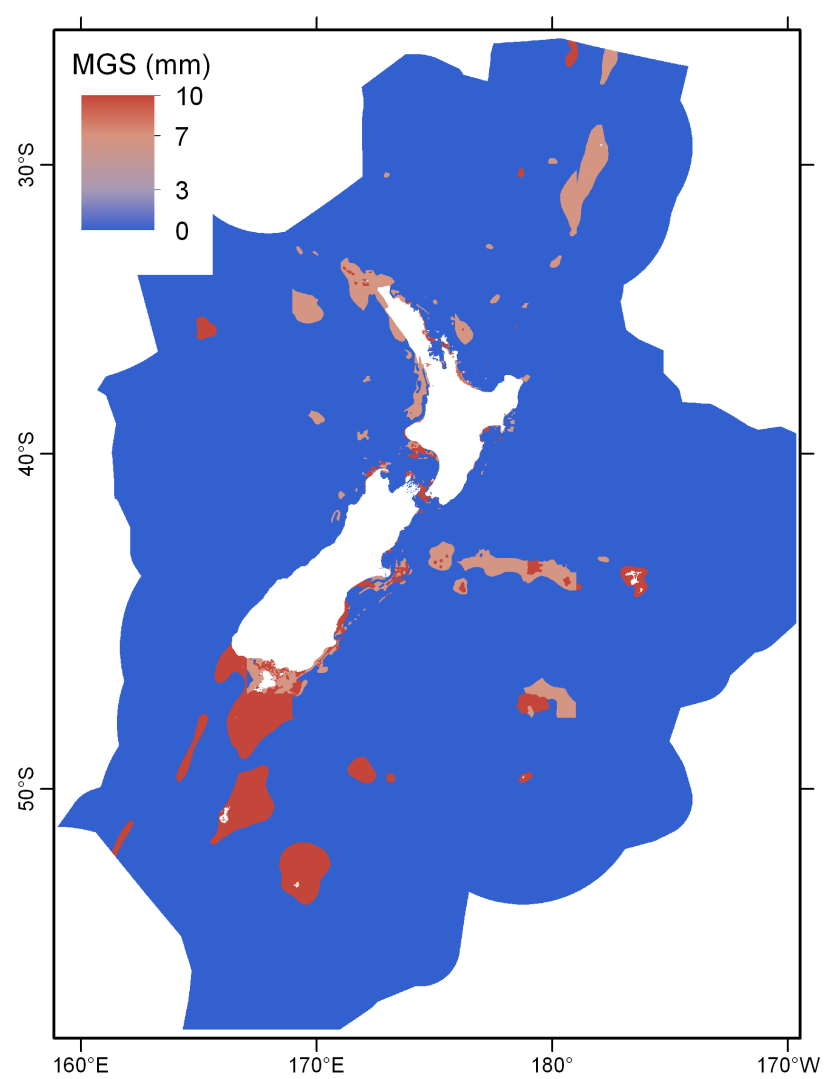

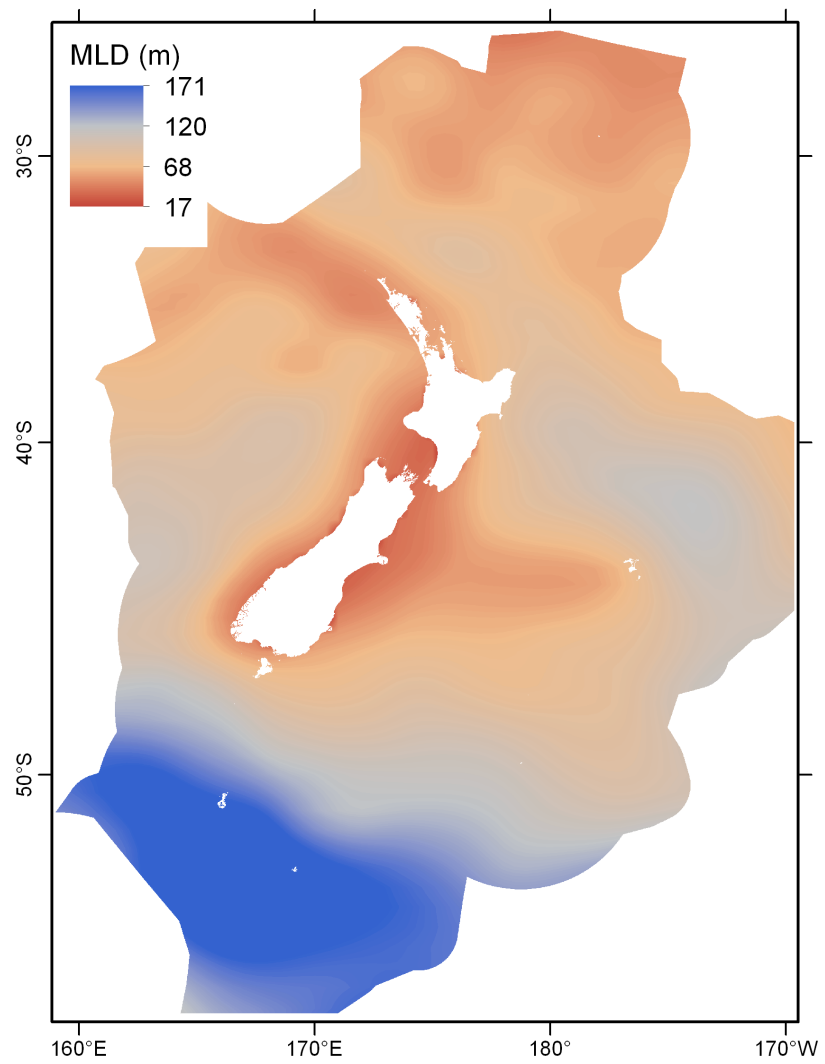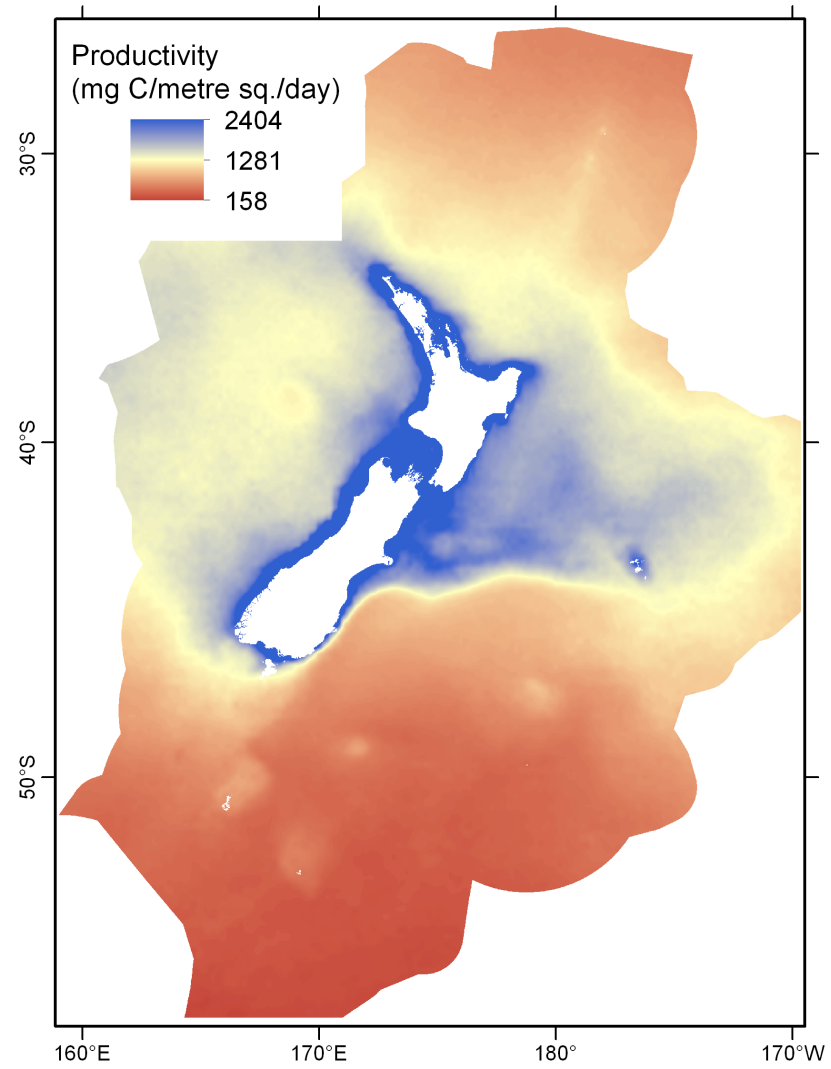

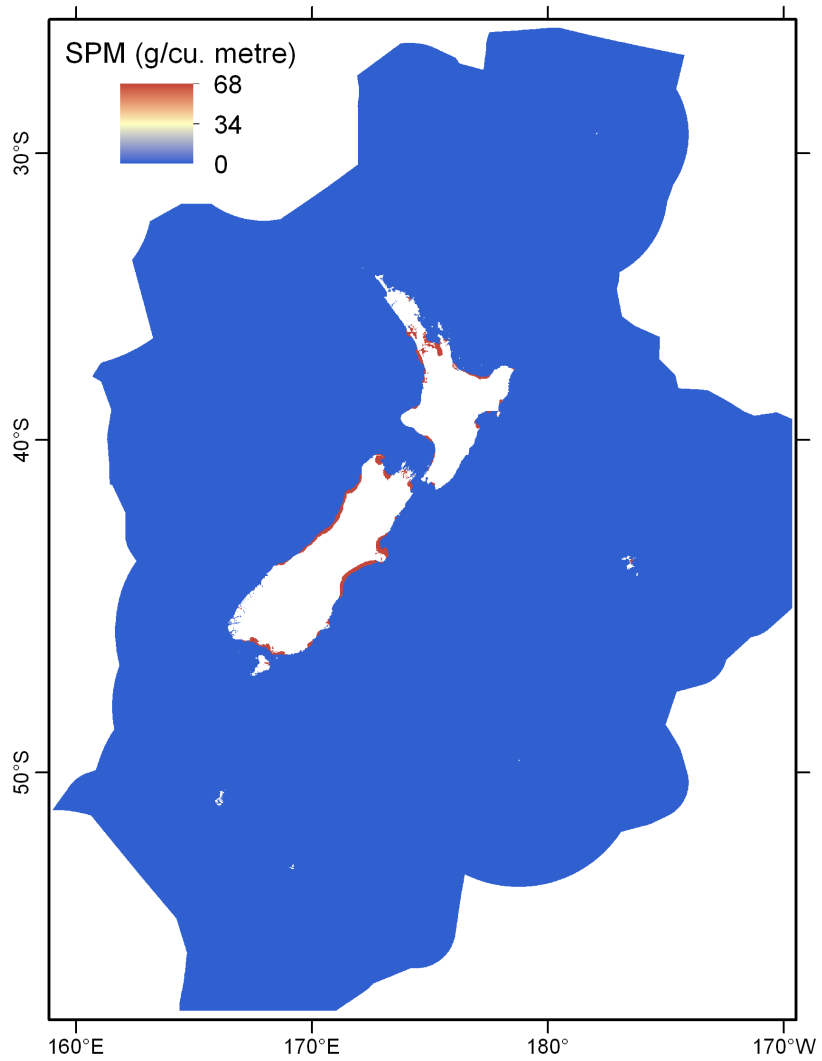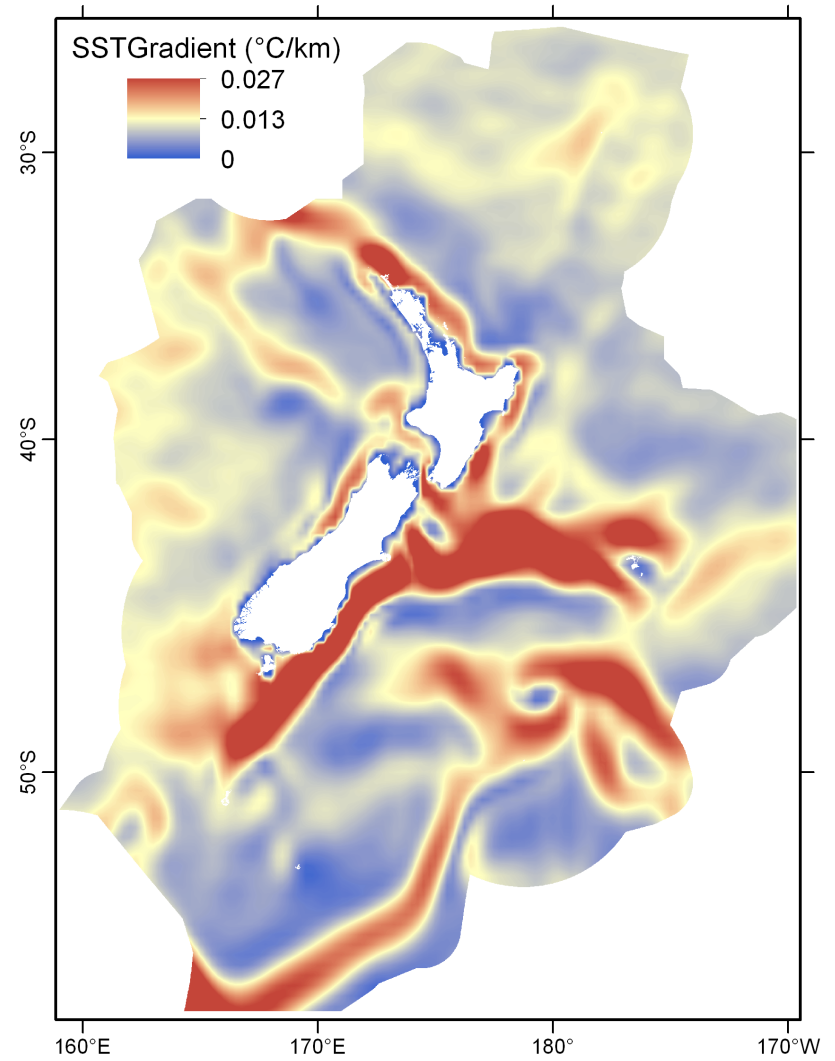

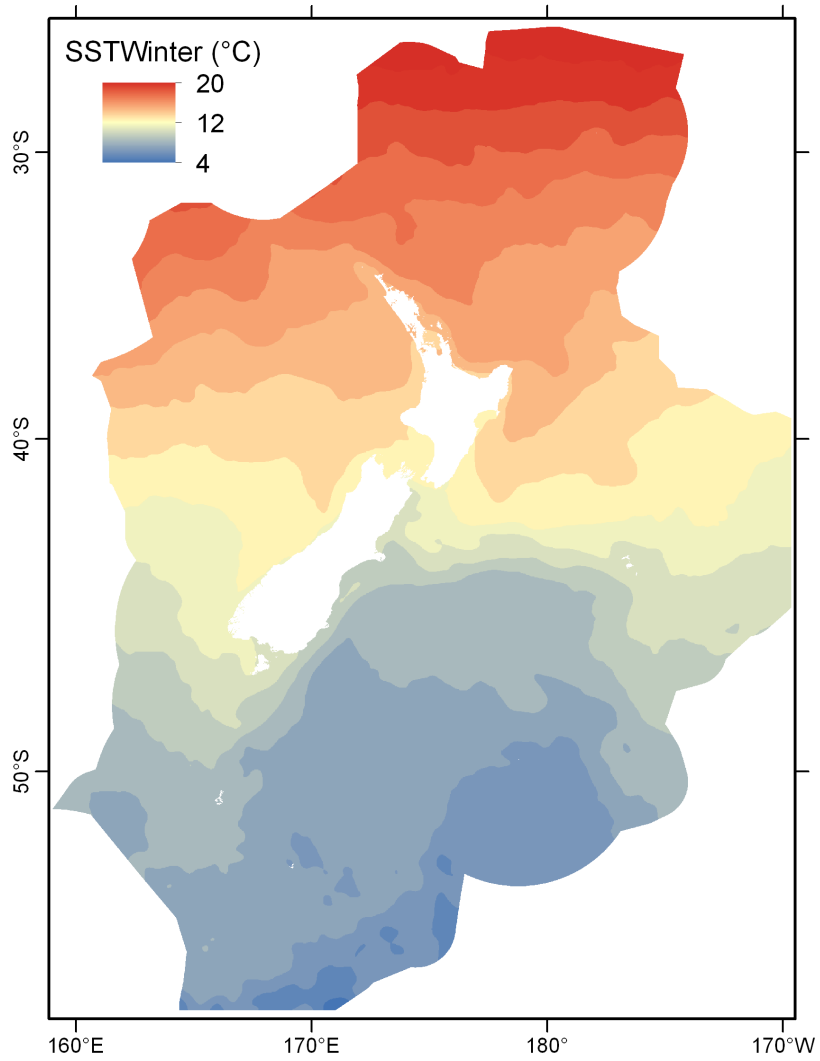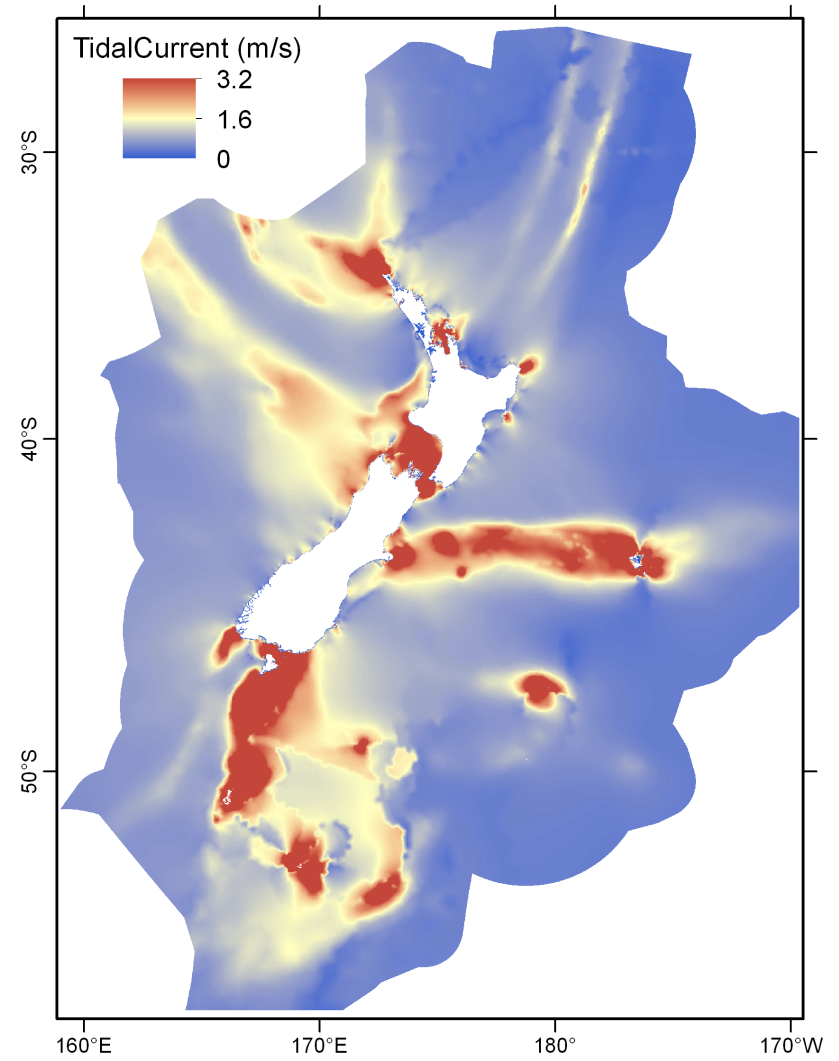

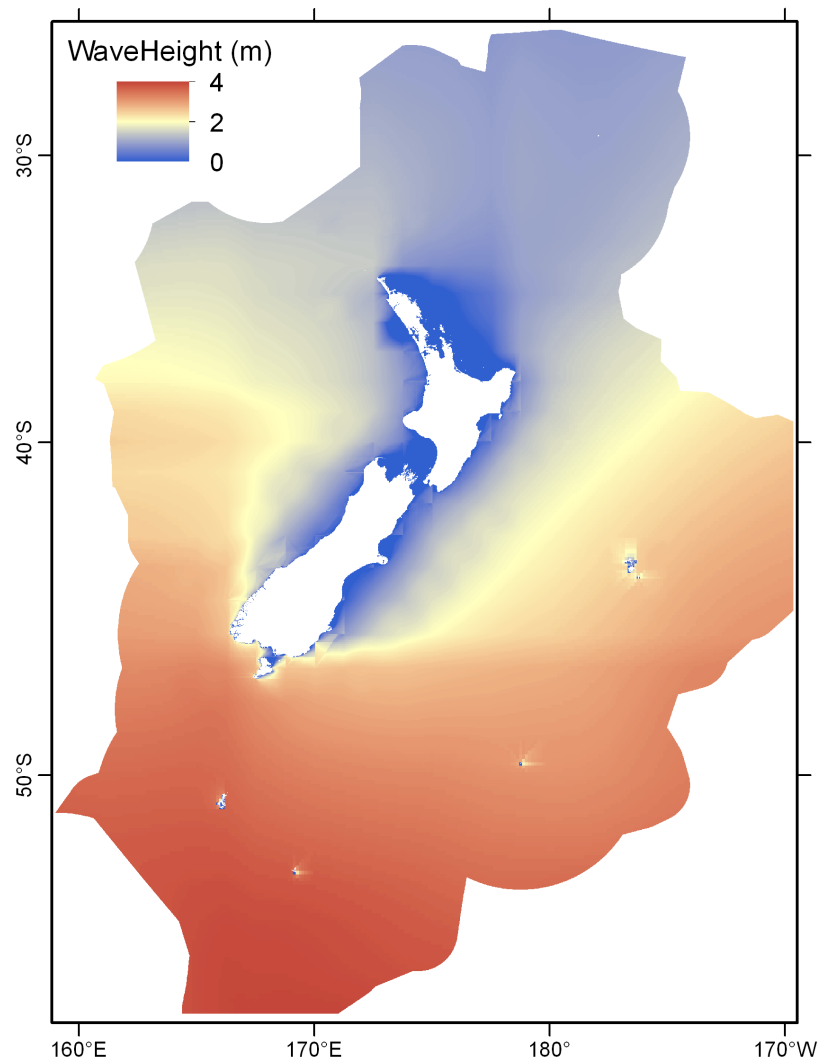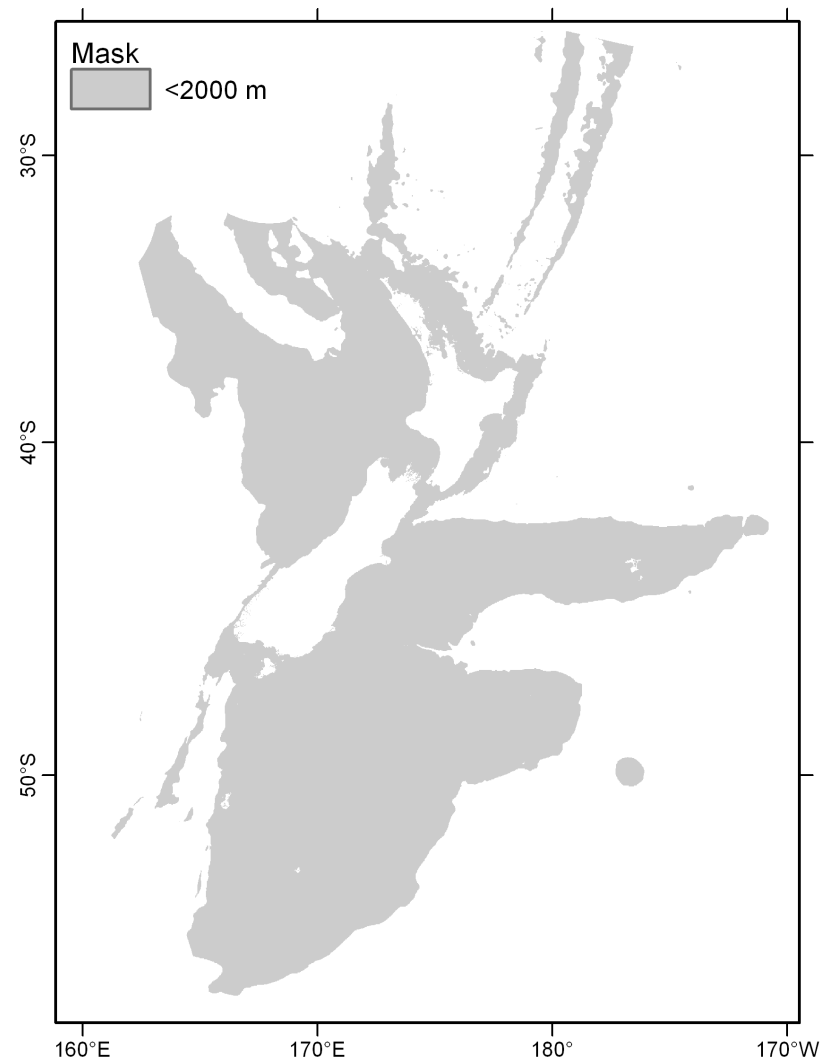

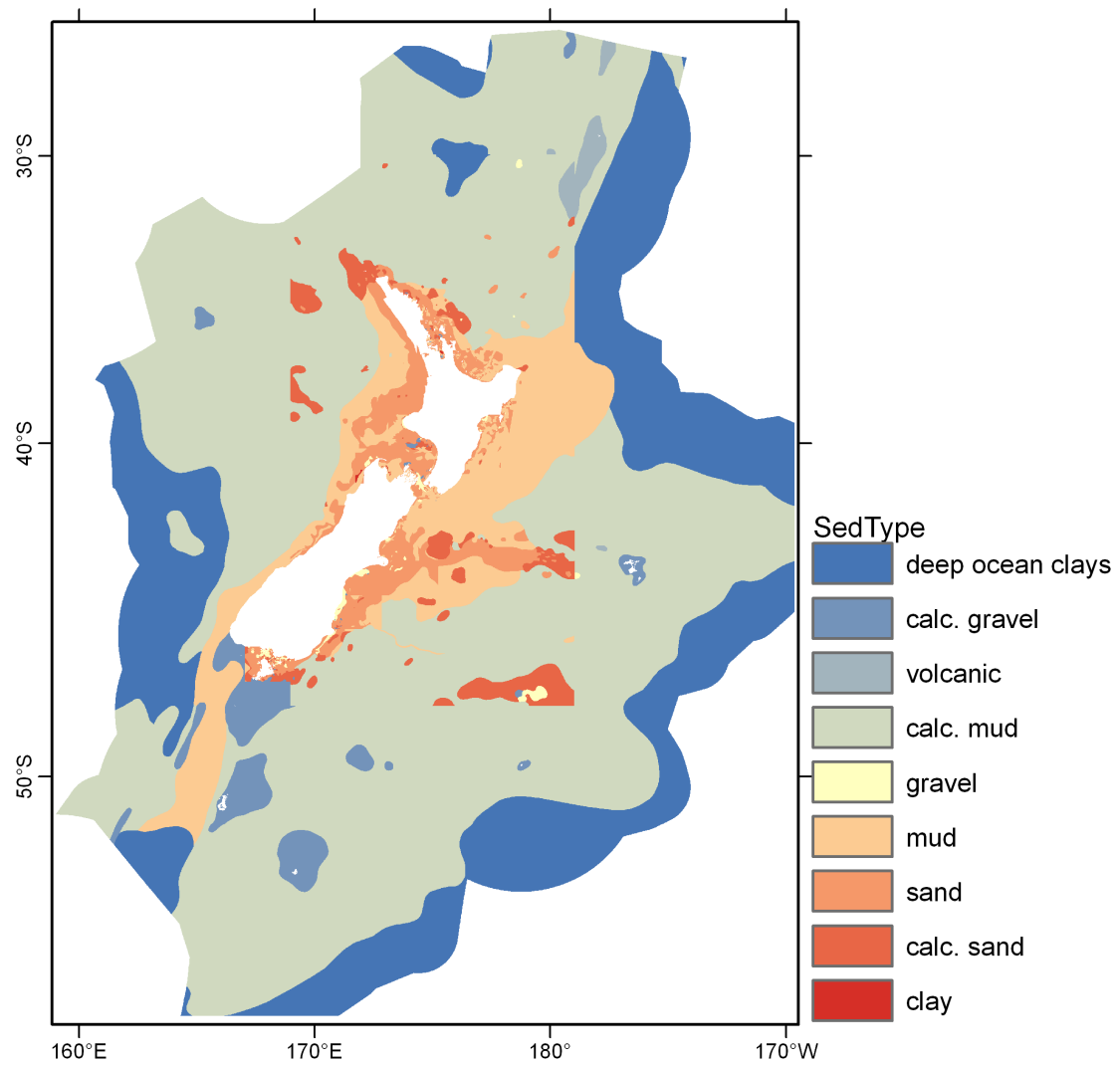

Supplement: Figure S1 — Images of the environmental layers used to model habitat suitability for habitat-forming bryozoans: Depth = water depth; MGS = median grain size; MLD = mixed layer depth; Productivity = surface water primary productivity; SPM = total suspended particulate matter concentration; SSTGradient = sea surface temperature gradient; SSTWinter = sea surface temperature in winter; TidalCurrent = depth averaged maximum tidal current; WaveHeight = annual average wave height; Mask, to prevent predictions to water depths >2000 m; and SedType = dominant sediment class. (PDF) [file pone.0075160.s001.pdf]

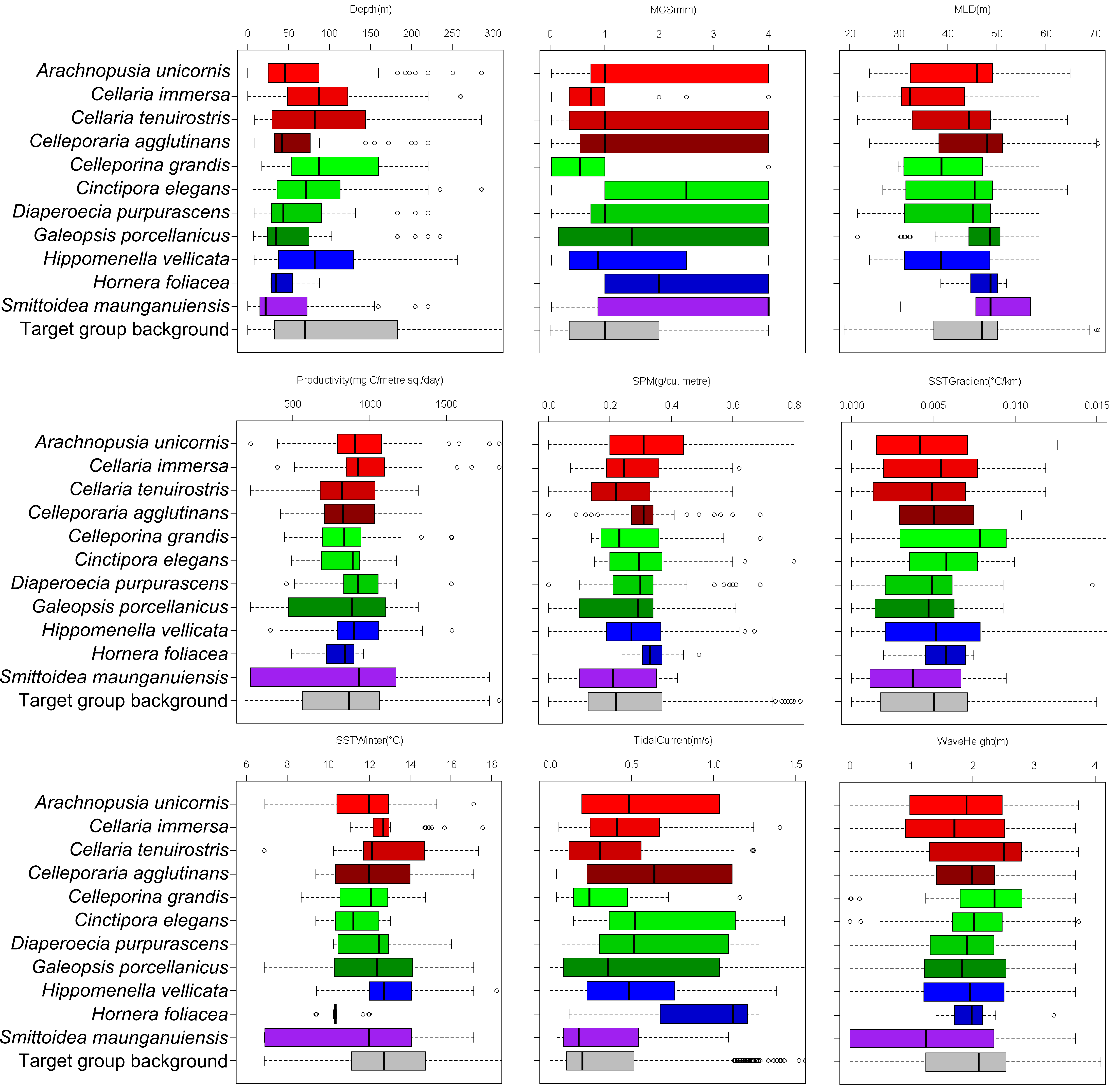

Supplement: Figure S2 — Box-and-whisker plots for each presence point of habitat-forming bryozoan species and for target-group background data, showing the distribution of values for each environmental layer. (TIF) [file pone.0075160.s002.tif]
