# Supplementary material for: Habitat-Forming Bryozoans in New Zealand: Their Known and Predicted Distribution in Relation to Broad-Scale Environmental Variables and Fishing Effort
Source: PLoS One. 2013 Sep 23;8(9):e75160. doi: 10.1371/journal.pone.0075160 (PMC3781067; doi:10.1371/journal.pone.0075160)

Arachnopusia unicornis

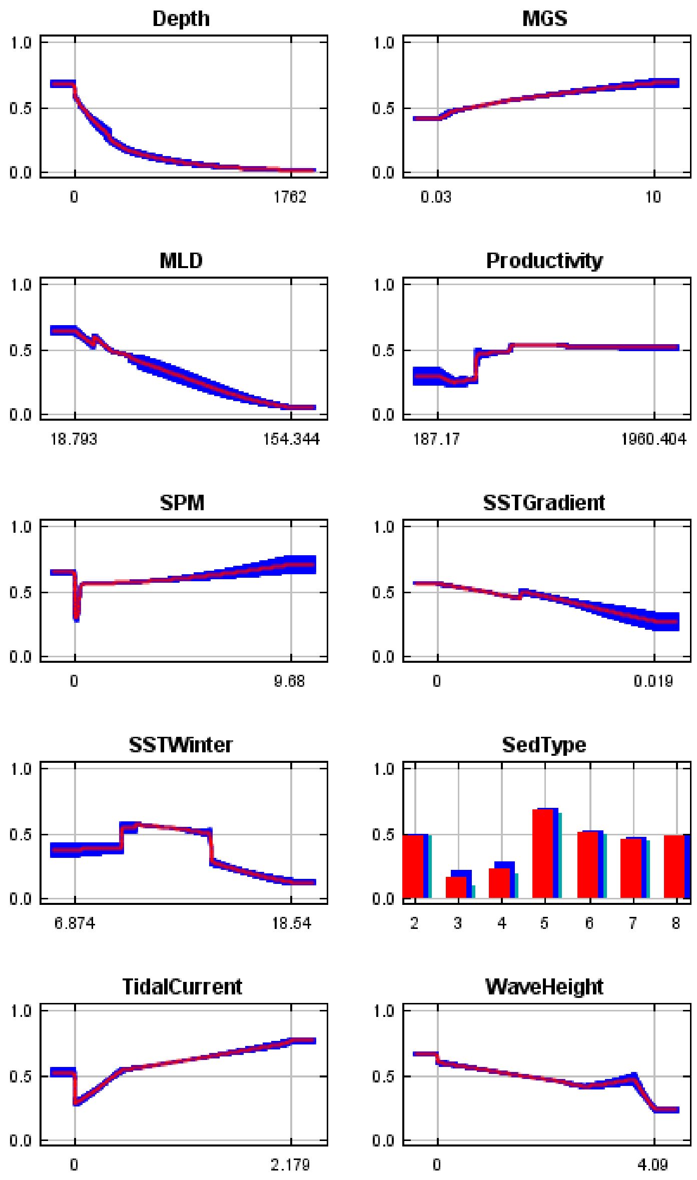

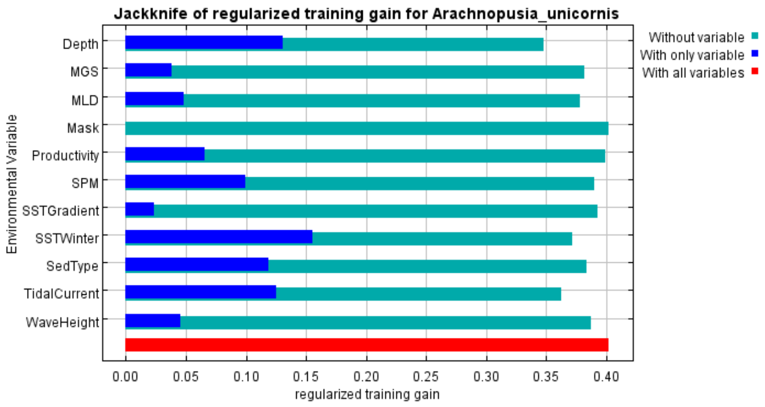

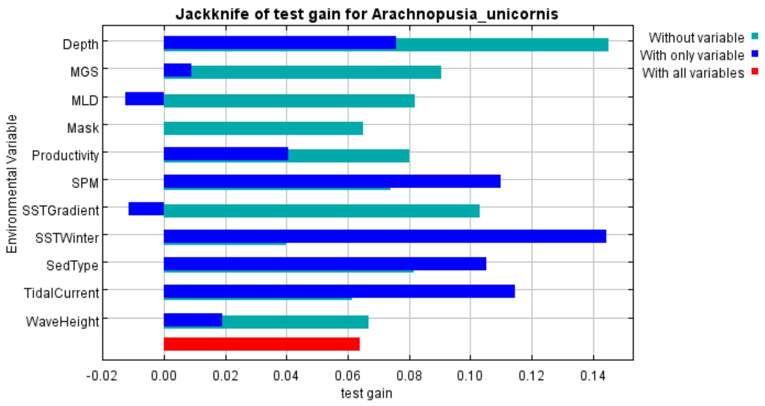

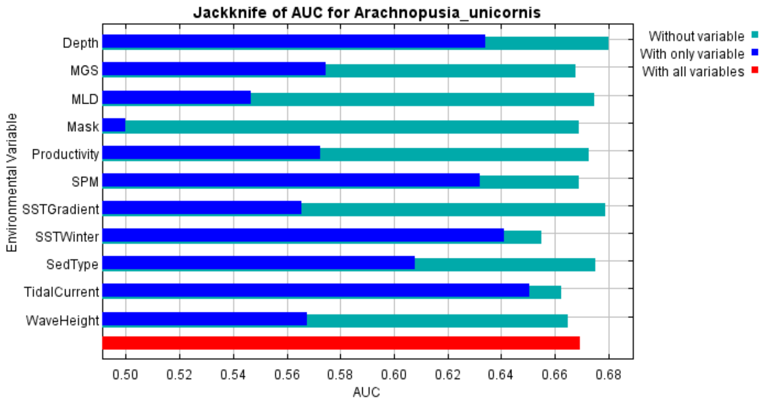

Cellaria immersa

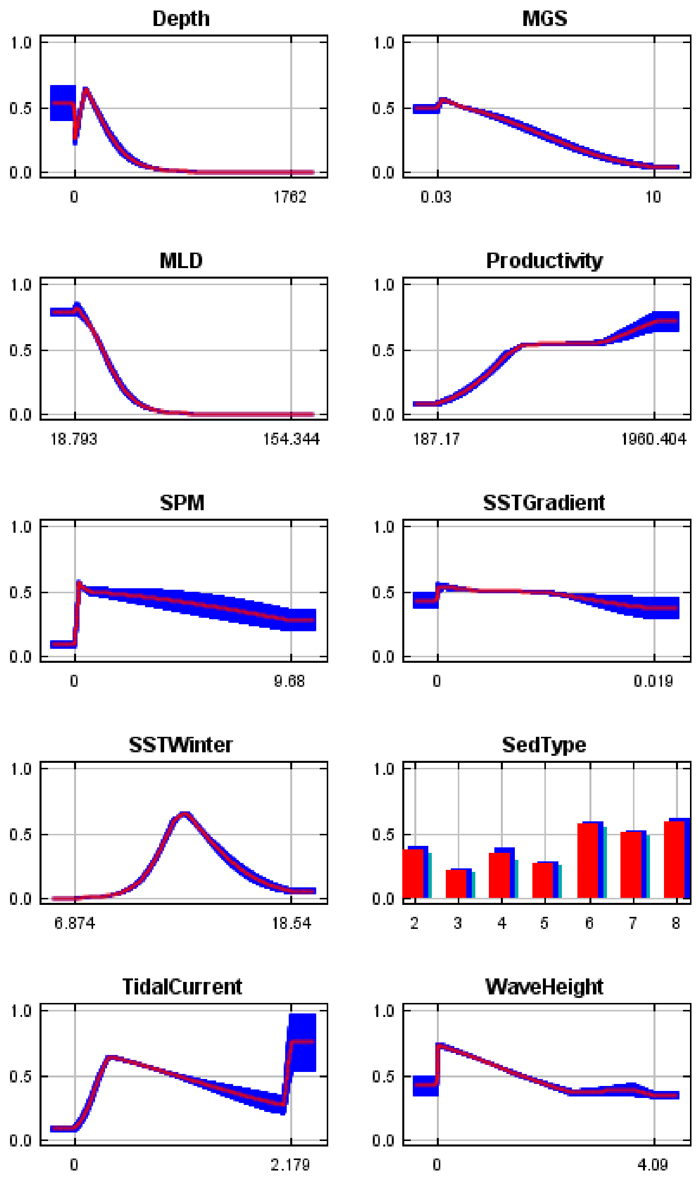

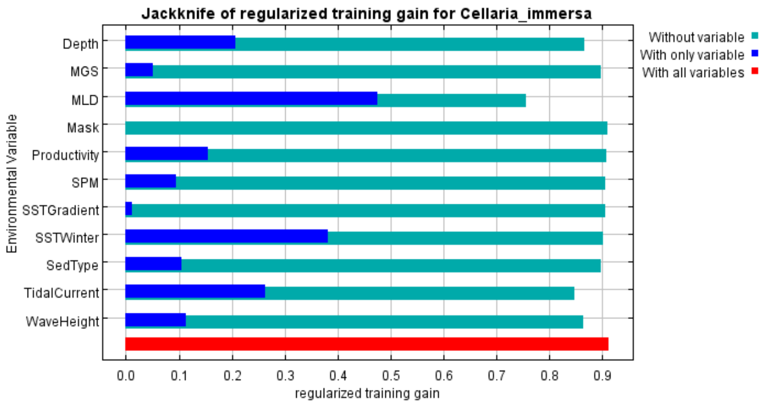

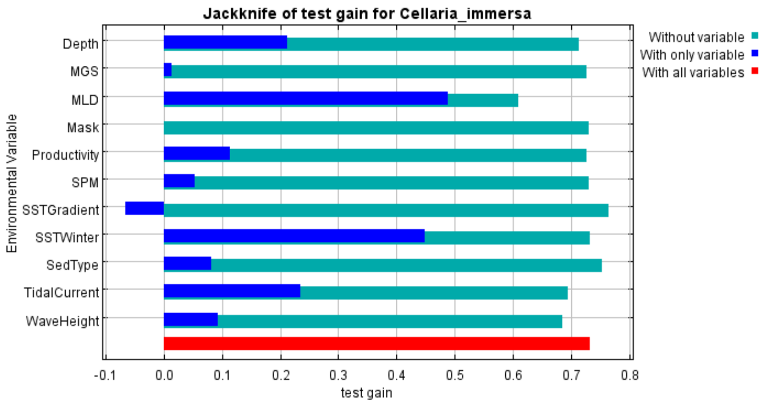

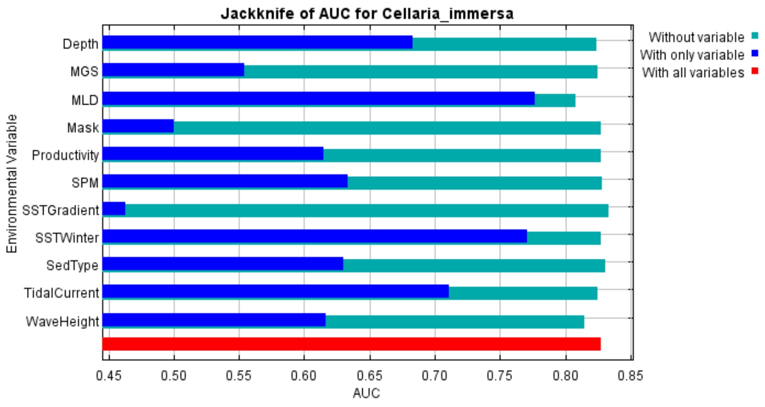

Cellaria tenuirostris

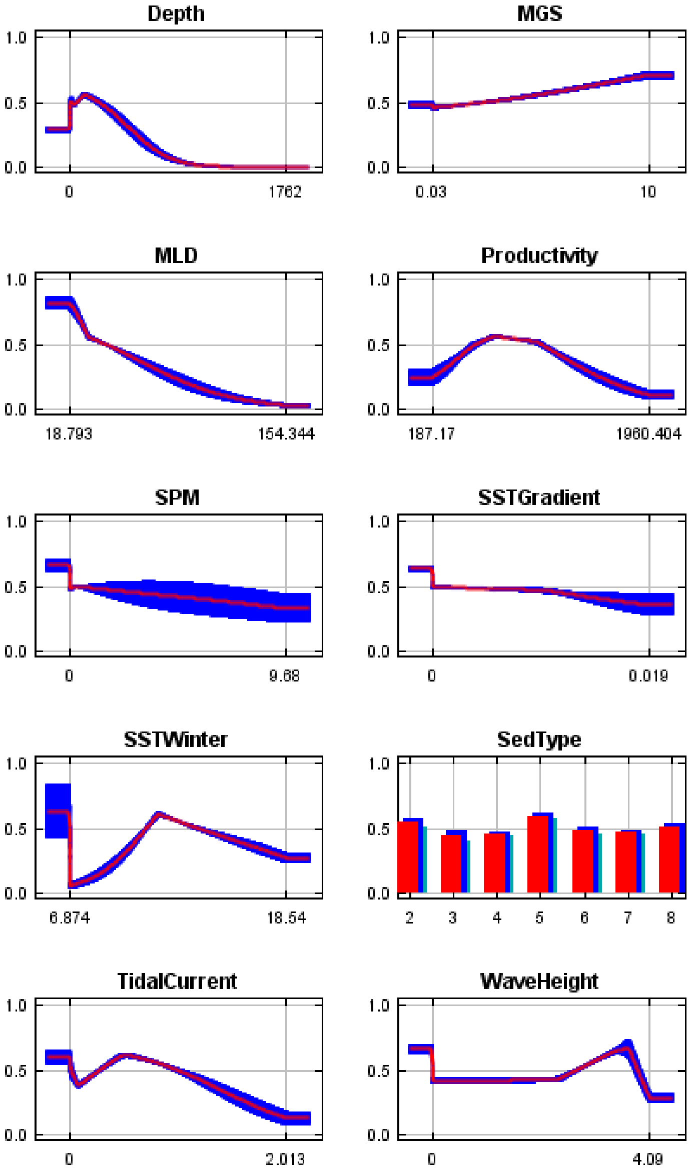

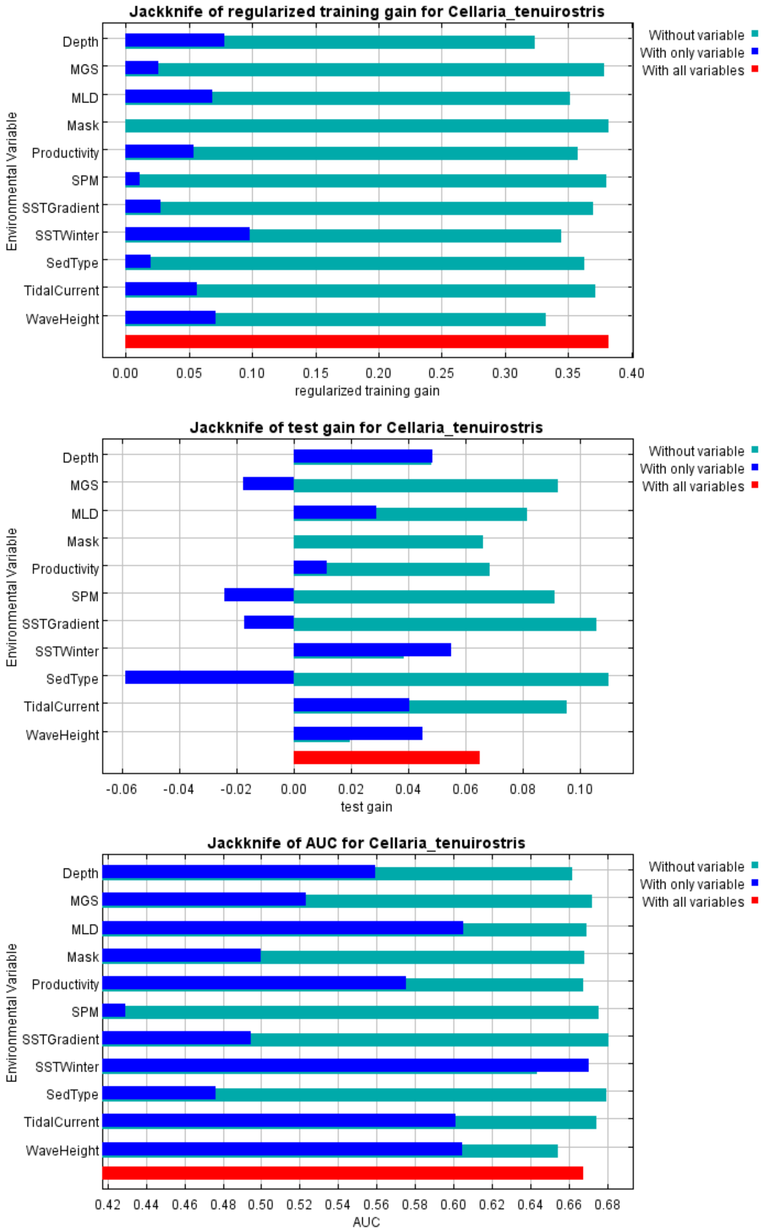

Celleporaria agglutinans

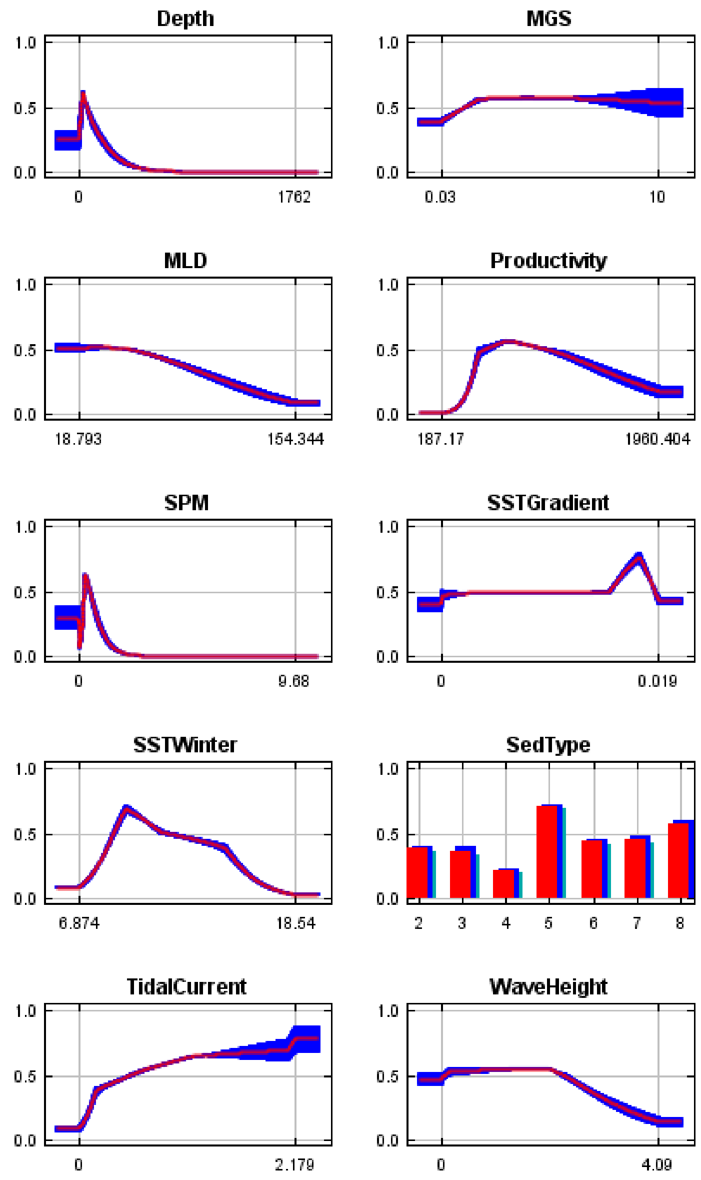

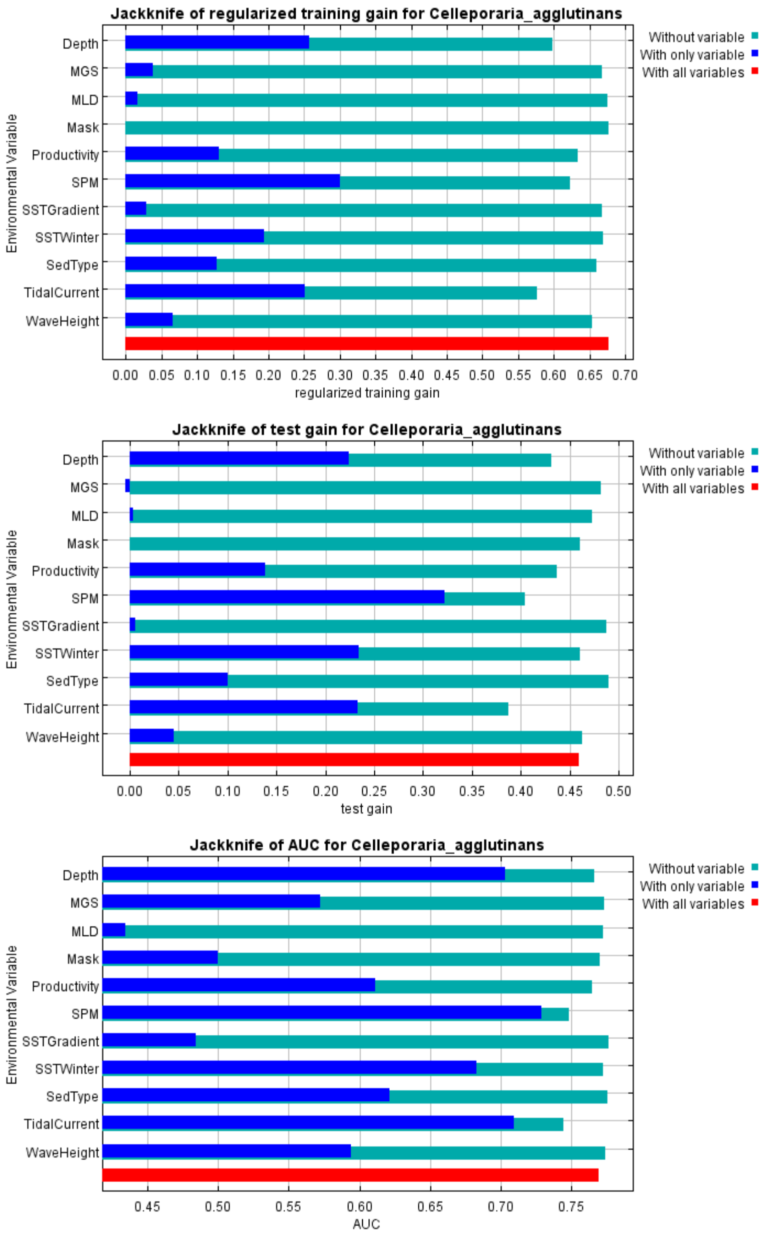

Celleporina grandis

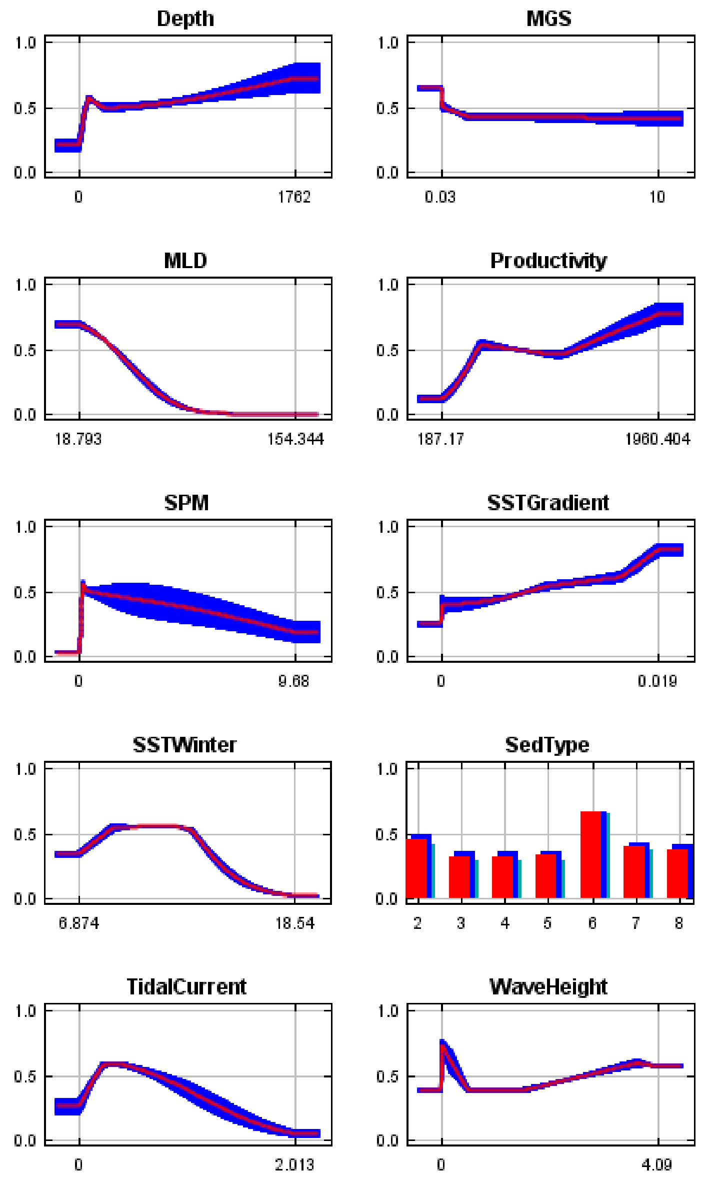

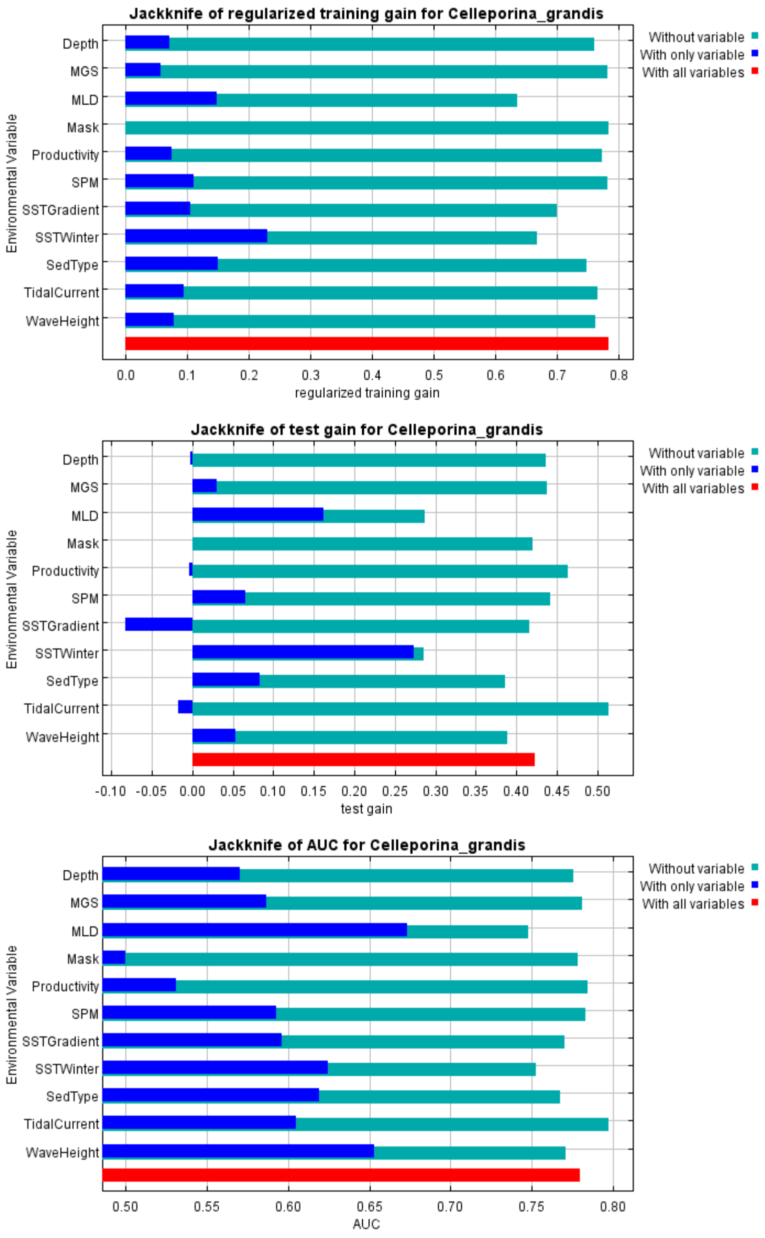

Cinctipora elegans

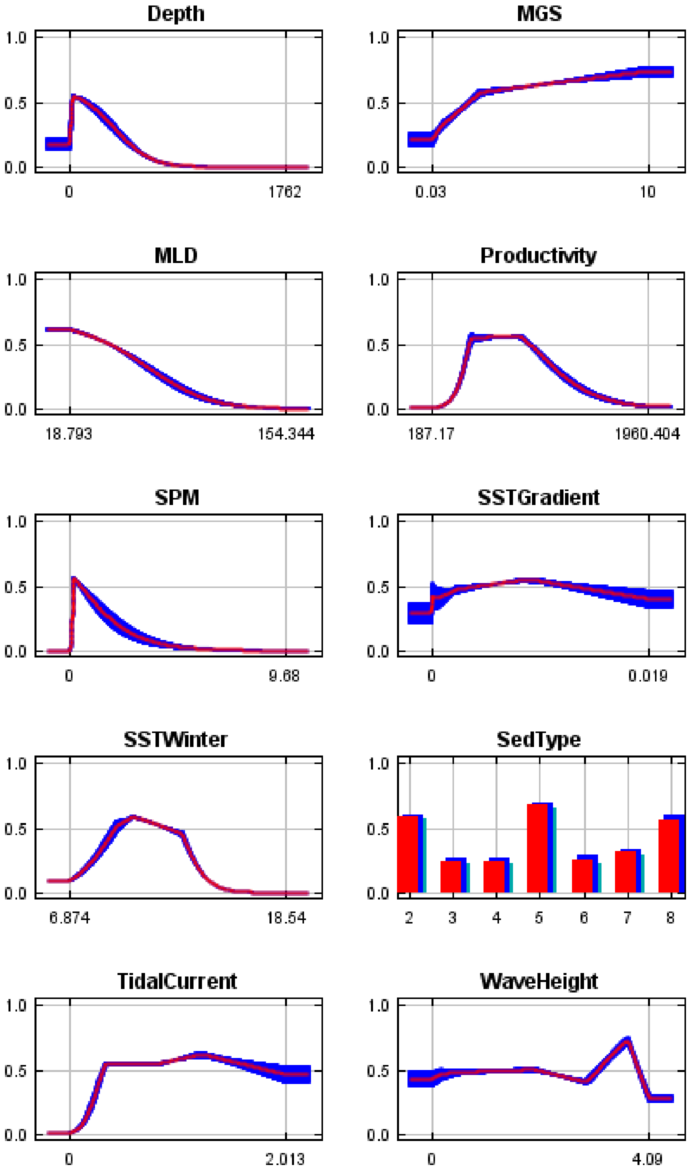

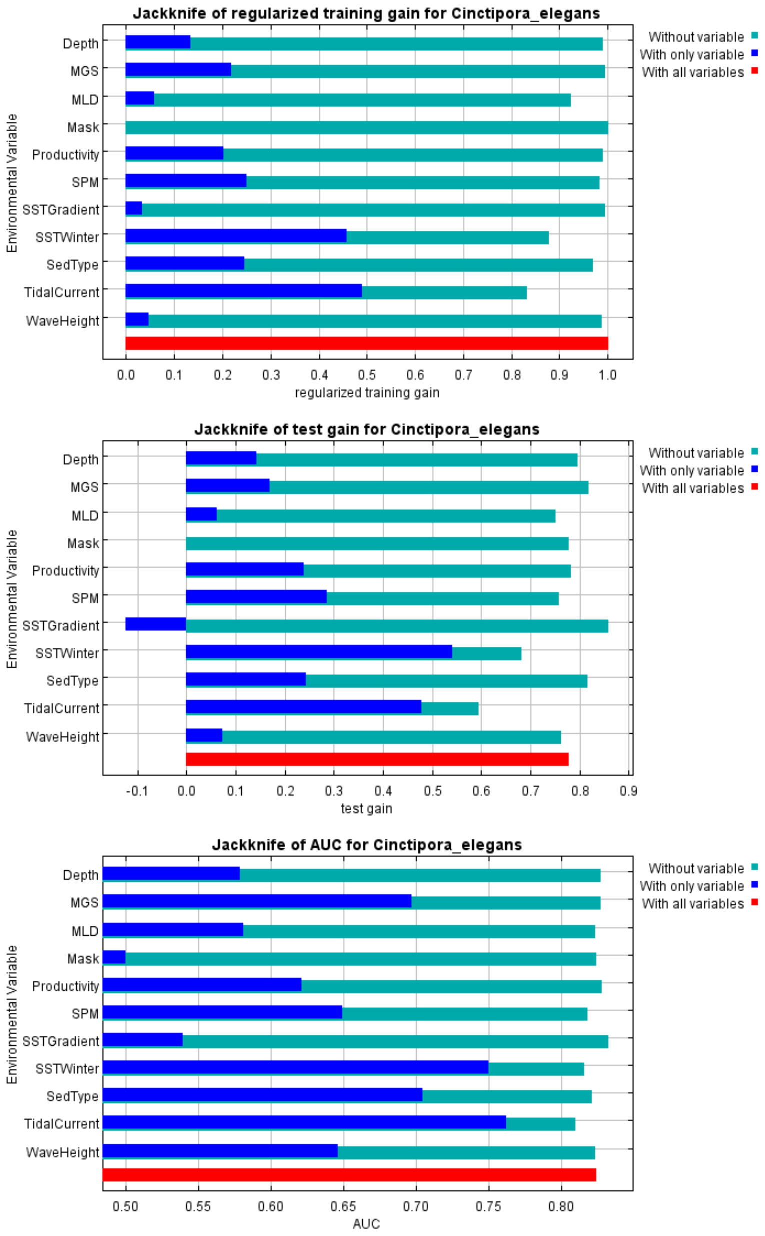

Diaperoecia purpurascens

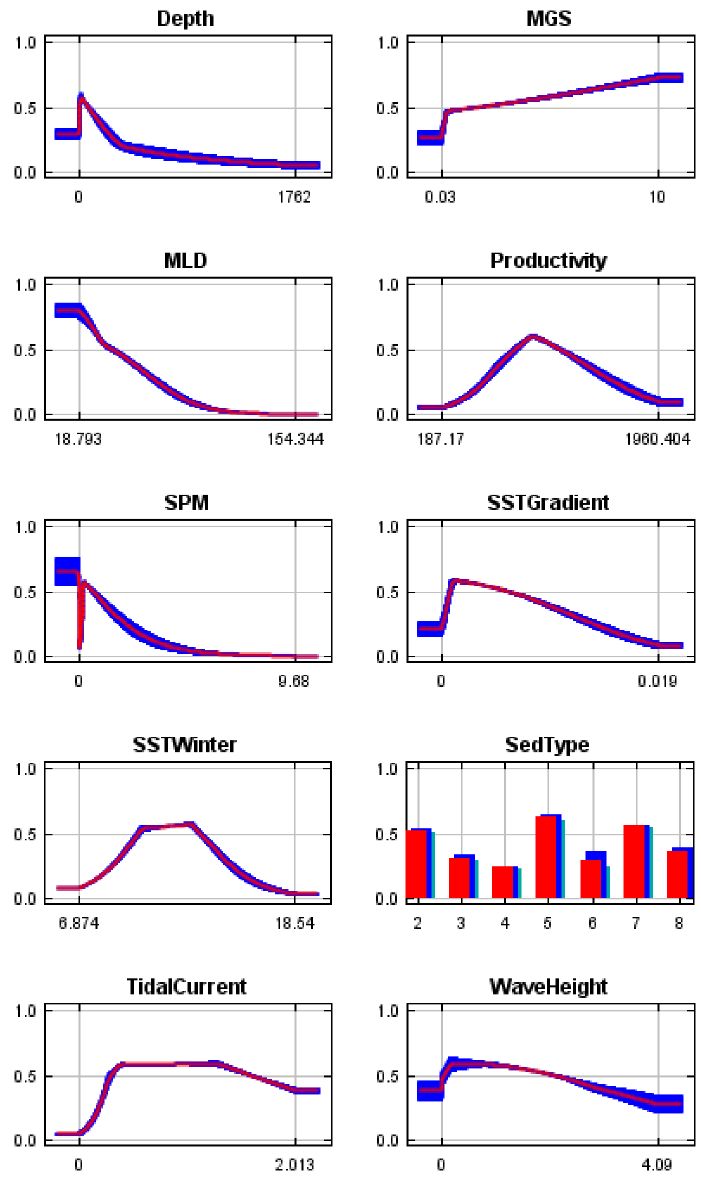

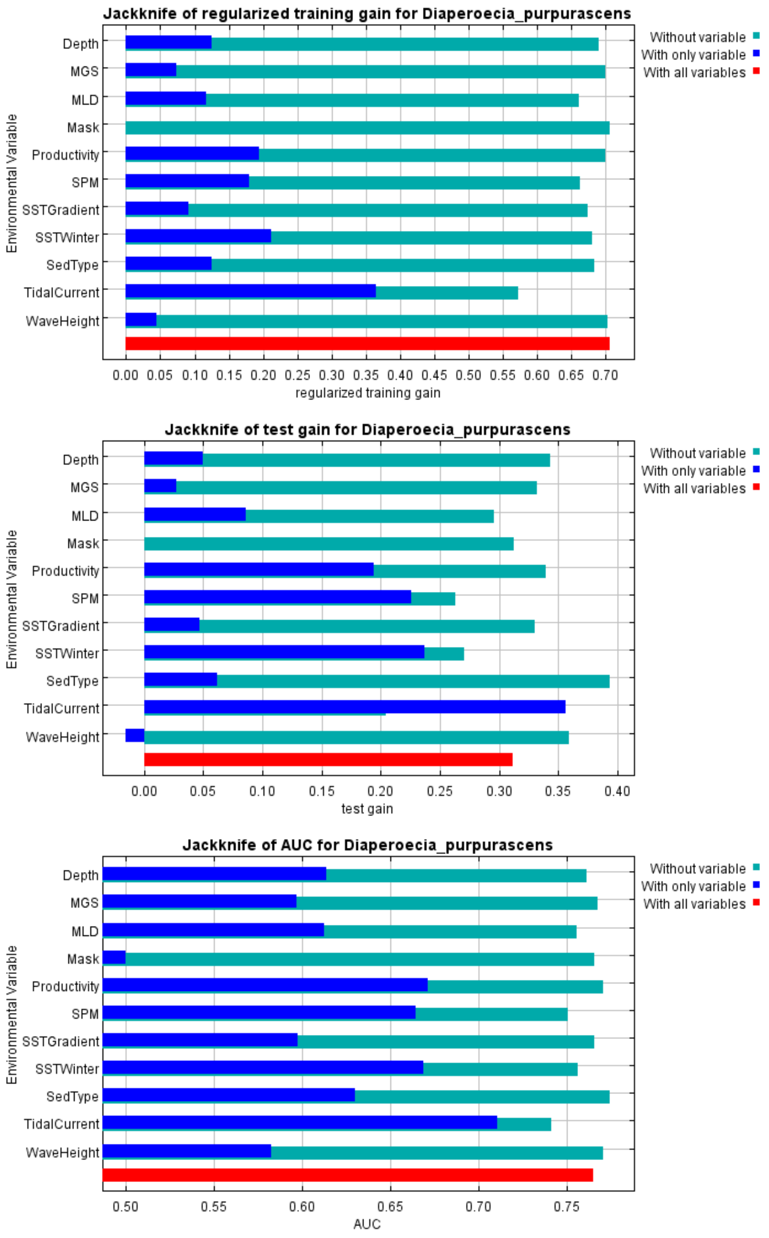

Galeopsis porcellanicus

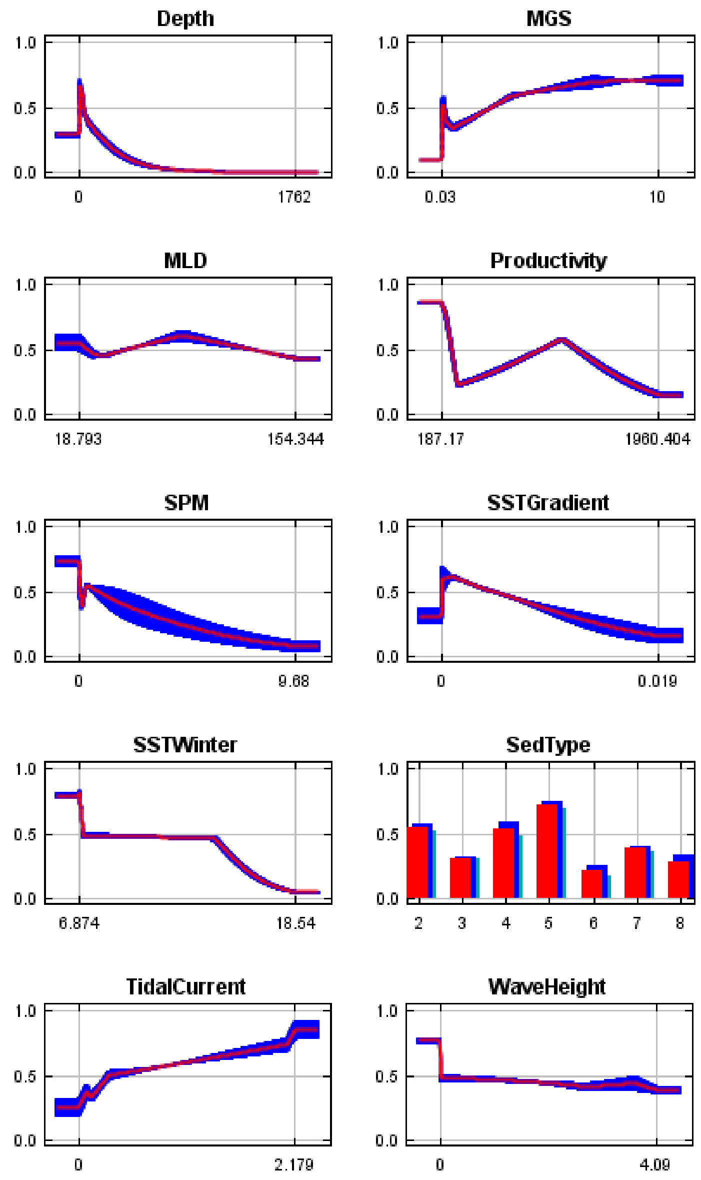

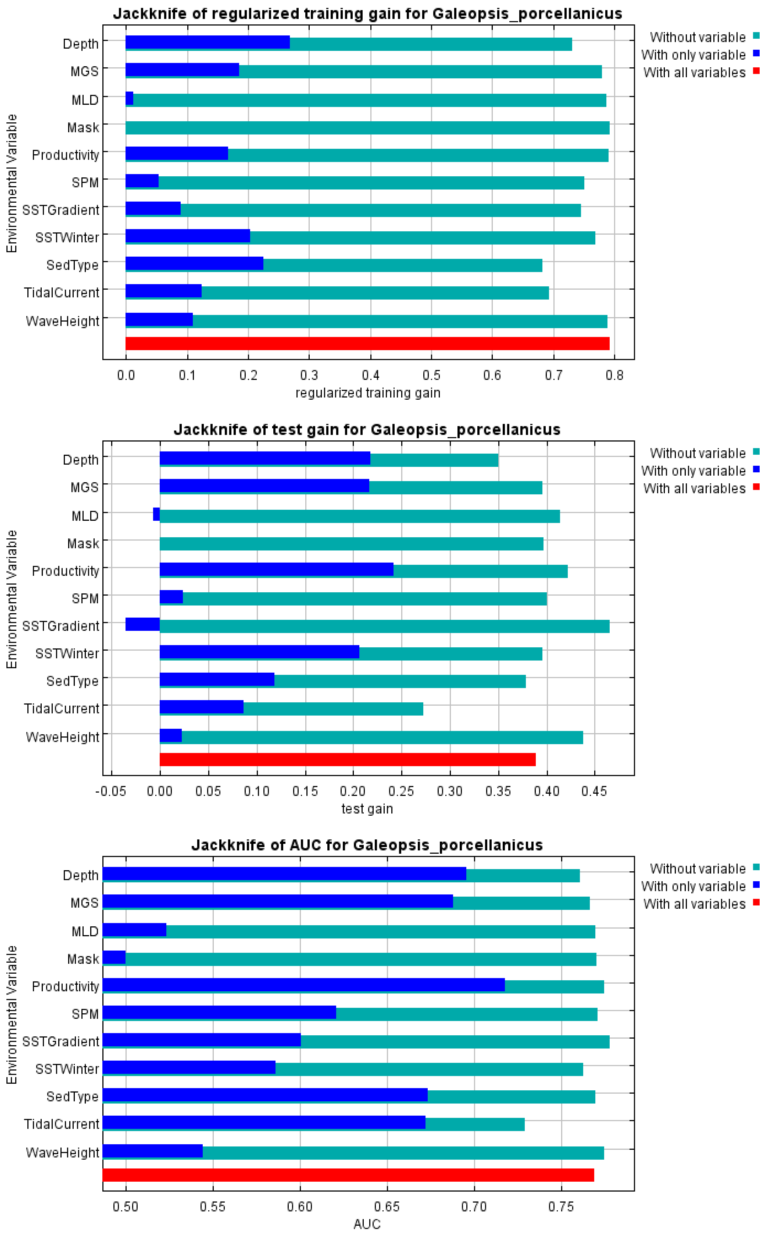

Hippomenella vellicata

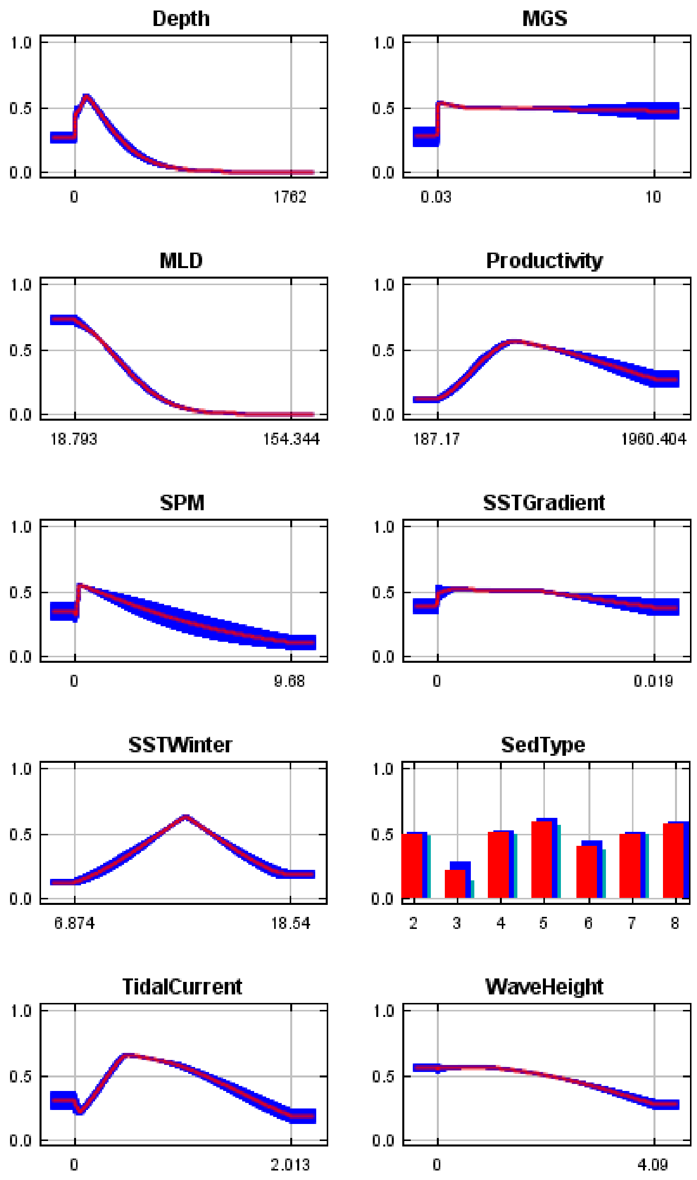

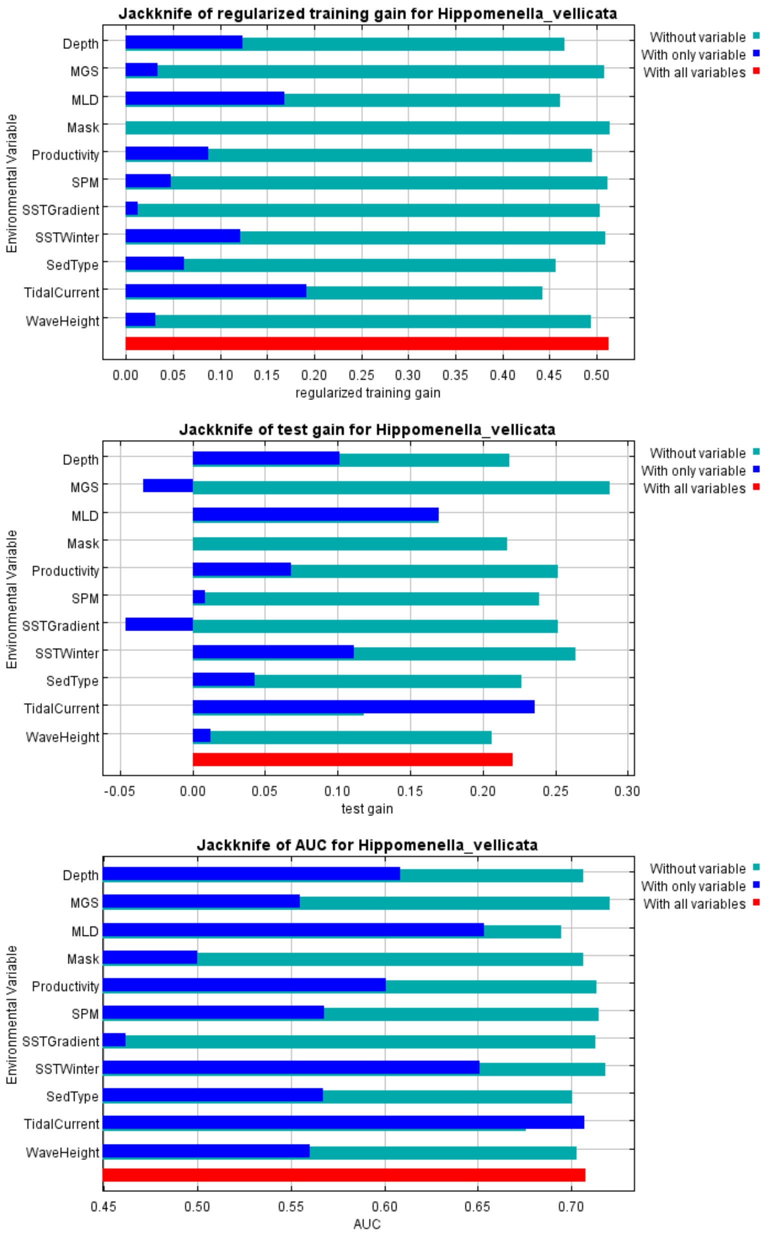

Hornera foliacea

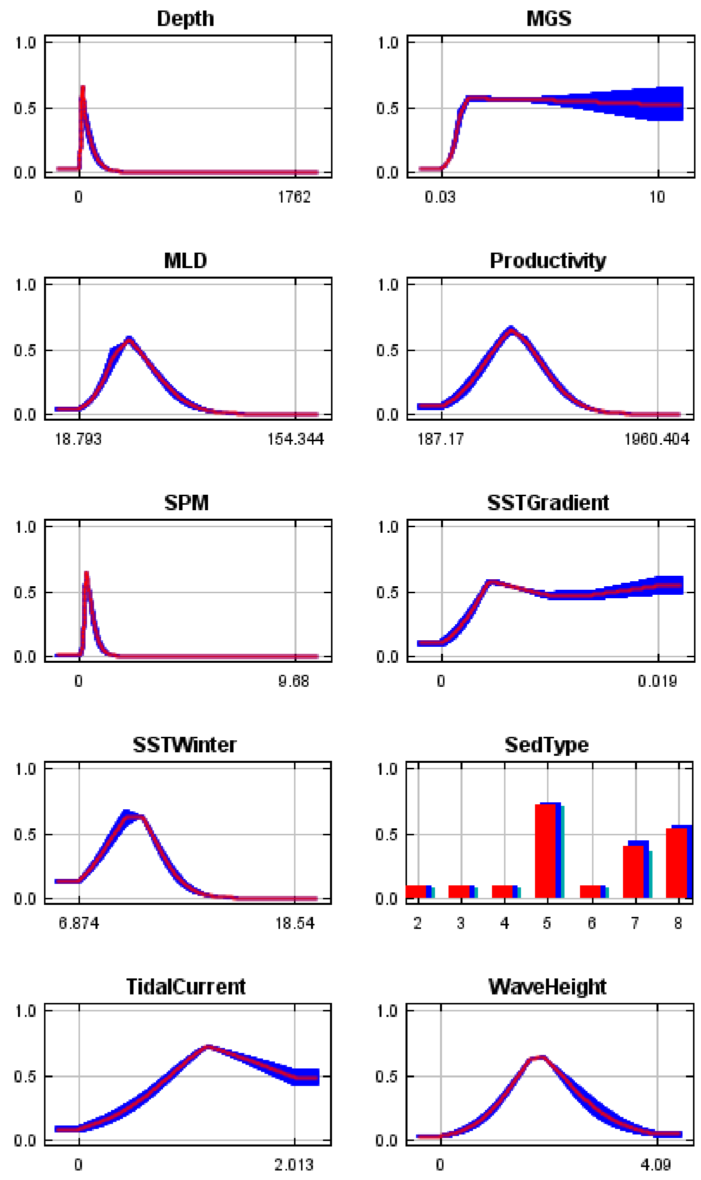

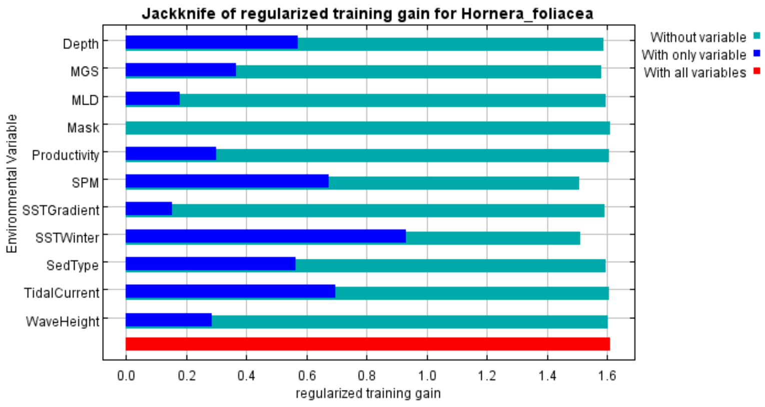

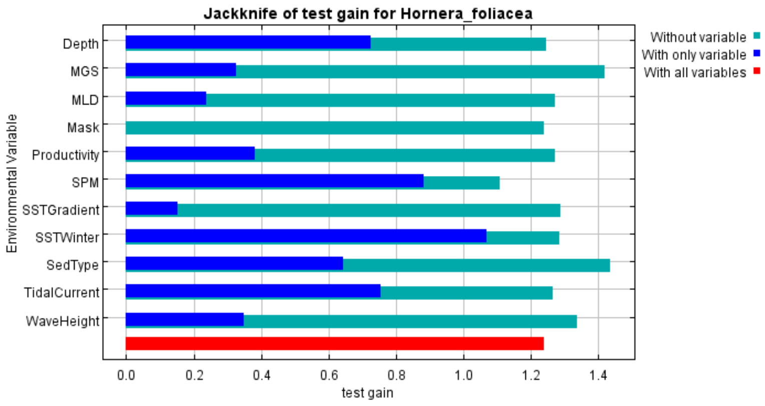

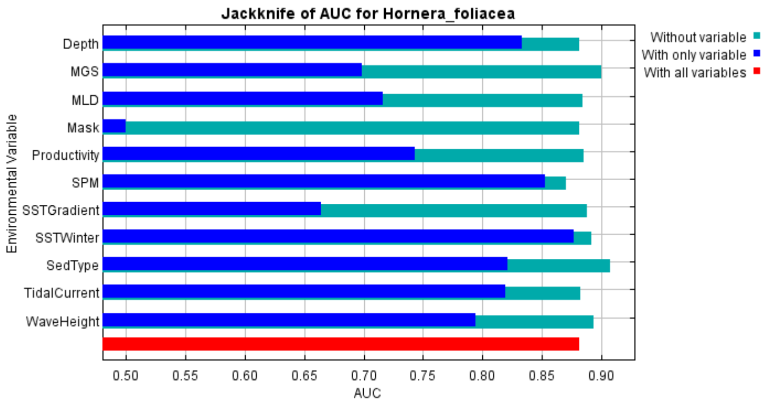

Smittoidea maunganuiensis

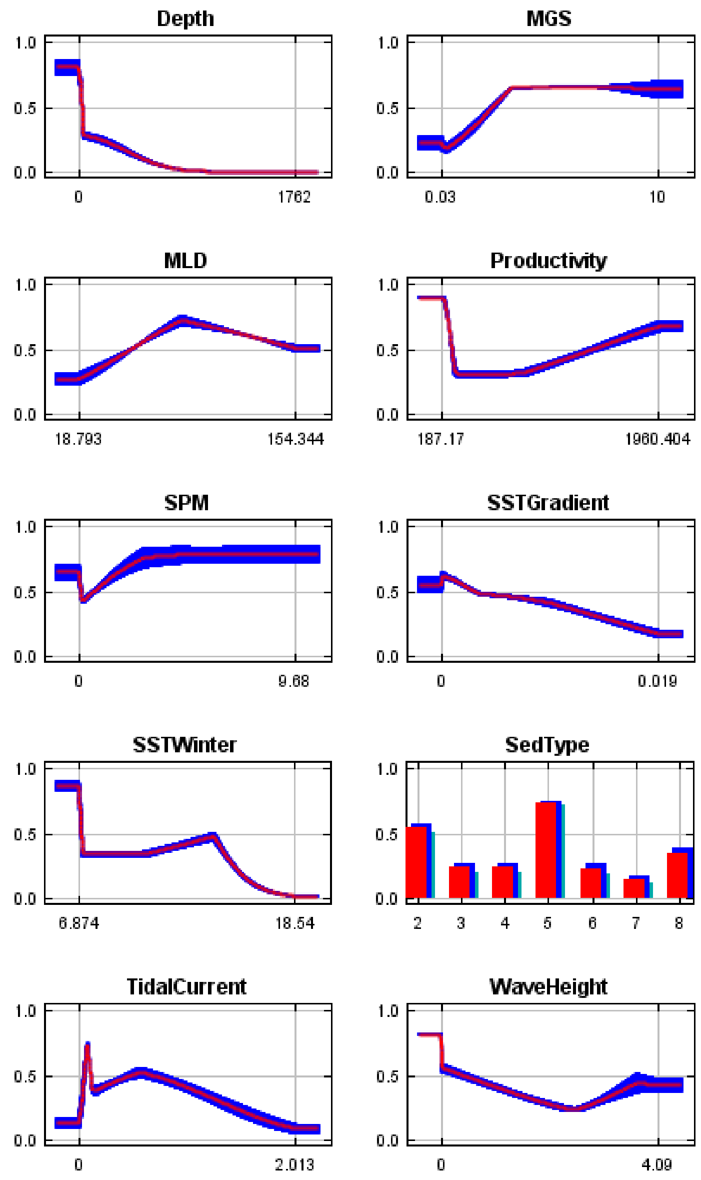

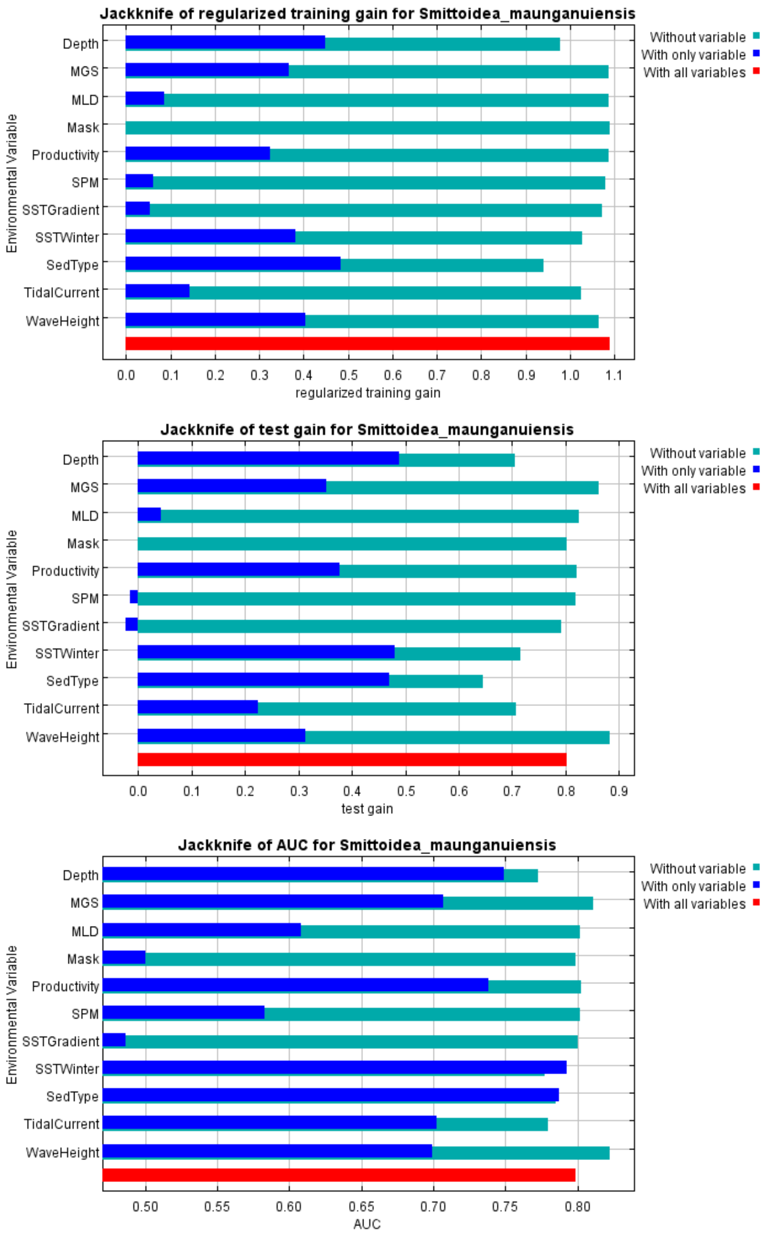

Supplement: Figure S3 — Fitted response curves (individual) for each habitat-forming bryozoan species, showing how predicted habitat suitability changed with different values of each variable. Together with the marginal response curves (Figures 3–13), these plots reflect the dependence of predicted suitability both on the selected variable and on dependencies induced by correlations between the selected variable and other variables. For the categorical variable sediment type: 1 = deep ocean clays; 2 = calcareous gravel; 3 = volcanic; 4 = calcareous mud; 5 = gravel; 6 = mud; 7 = sand; 8 = calcareous sand. Jackknife tests show the importance of each variable to the training and test gain, and to AUC. (PDF) [file pone.0075160.s003.pdf]
