# Supplementary material for: Habitat-Forming Bryozoans in New Zealand: Their Known and Predicted Distribution in Relation to Broad-Scale Environmental Variables and Fishing Effort
Source: PLoS One. 2013 Sep 23;8(9):e75160. doi: 10.1371/journal.pone.0075160 (PMC3781067; doi:10.1371/journal.pone.0075160)
Supplement: Text S1 — Detailed descriptions of model validation, and the distributions of suitable habitat, for each species. (PDF) [file pone.0075160.s017.pdf]

S5. Detailed descriptions of model validation, and the distributions of suitable habitat of for each species.

Arachnopusia unicornis

Model validation

The mean AUC of the predictive model was 0.669, meaning the model was just below the 'useful' threshold. Test gain was also low (0.064), and test omisson rate was high (>20%). SST in winter, tidal current, and to a lesser extent wave height, were the most effective variables for predicting the distribution of the test data when predictive performance was measured using AUC. SST in winter, and tidal current were the most effective variables for predicting the distribution of the test data when predictive performance was measured using test gain. Jackknifing showed AUC would increase if depth, mixed layer depth, surface water productivity, SST gradient, and dominant sediment type were removed from the model but removing any one would not increase the AUC above 0.7. Test gain would increase significantly if depth, median grain size, surface water productivity, suspended particulate matter, dominant sediment type and particularly SST gradient and mixed layer depth were removed from the model.

Known and predicted distribution

The majority of records for *Arachnopusia unicornis* were concentrated around northern North Island, Hauraki Gulf, Greater Cook Strait, Foveaux Strait, Puysegur 'Bank', and Fiordland. Occasional records for this species were also obtained from Kermadec Ridge, Otago shelf, and near the Chatham Islands and Antipodes Island Group. The predicted distribution partially reflected the distribution of presence records. The most extensive areas of suitable-to-favourable habitat were predicted around the Bounty Islands, Greater Cook Strait, Foveaux Strait, Puysegur 'Bank', off Banks Peninsula, and Mernoo Bank. An area around the Bounty Islands was notable for its size of highly suitable habitat. Smaller, favourable habitat areas were predicted along the east coast of much of South Island, off Kahurangi Point, and around the Chatham Islands. There were no *Arachnopusia unicornis* presence records near an extensive area of habitat predicted to occur around Banks Peninsula, whilst the species was recorded off the northern North Island, in the Hauraki Gulf and along the Fiordland coast, where very little suitable habitat was predicted.

Cellaria immersa

Model validation

The mean AUC value was 0.827, and test omisson rate was low (10.5%) meaning the model is useful for predicting the distribution of *Cellaria immersa*. Mixed layer depth, and to a lesser extent wave height were the most effective variables for predicting the distribution of the test data when predictive performance was measured using AUC. Mixed layer depth, and to a lesser extent tidal current and wave height were most effective variables for predicting the distribution of the test data when predictive performance was measured using test gain (value 0.733). Jackknifing showed both AUC and test gain would increase slightly if SST gradient and dominant sediment type were removed, and SST gradient on its own had a negative affect on test gain.

Known and potential distribution

Presence records for *Cellaria immersa* were concentrated around northern North Island, Greater Cook Strait, and on Puysegur 'Bank'. Occasional records for this species were obtained from Kermadec Ridge, the Hauraki Gulf and Coromandel Peninsula, and the Fiordland coast. The modelled distribution partially reflected the distribution of presence records. A single extensive favourable-to-

highly suitable habitat area in Greater Cook Strait was predicted, and smaller areas of average suitability were predicted off the northern North Island, parts of South Island's east coast (including around Banks Peninsula, Canterbury Bight and Mernoo Bank), small areas off Puysegur 'Bank' and in western Foveaux Strait. The predicted areas off the east coast of South Island, and in western Foveaux Strait had no associated presence records, whilst a presence record on the outer Coromandel Peninsula was not associated with any prediction of suitable habitat.

Cellaria tenuirostris

Model validation

The mean AUC (0.668) was too low for the predictive model to be used. Test gain was also low at 0.065, however, test omisson rate was reasonably low (13%). Depth, SST in winter and wave height were the most effective variables for predicting the distribution of the test data when predictive performance was measured using AUC, although SST in winter on its own achieved an AUC value equal to that of the full model. These variables were also the most effective for predicting the distribution of the test data when predictive performance was measured using test gain. Jackknifing showed AUC could be slightly improved by removing the variables median grain size, suspended particulate matter, SST gradient, dominant sediment type, and tidal current, but removing any single variable did not increase the AUC value beyond 0.7. Jackknifing of test gain showed median grain size, suspended particulate matter, SST gradient, and dominant sediment type all had a negative effect on test gain, but gain could also have been increased by the removal of mixed layer depth, surface water productivity, and tidal current.

Known and potential distribution

Presence records for *Cellaria tenuirostris* were concentrated around the northern North Island, Greater Cook Strait, on Puysegur 'Bank' and through Fiordland, with minor concentrations on Kermadec Ridge, the Hauraki Gulf/outer Coromandel Peninsula, and Foveaux Strait. One record was obtained from near the Antipodes Island Group. The recorded distribution only partially described the predicted distribution. The largest area predicted as being of average suitability was in Greater Cook Strait, but a large, favourable habitat area was predicted along the south coast of South Island, and along the western side of Stewart Island/Rakiura. Smaller areas of average habitat suitability include the west side of the northern North Island, Mernoo Bank, the Chatham and Bounty Islands, with small areas being predicted as more favourable at the Bounty Islands and Puysegur 'Bank'. The areas of suitable habitat predicted west of Taranaki, at Mernoo Bank, and at the Chatham and Bounty Islands had no presence records. Presence records in areas where habitat was predicted as unsuitable occurred at the Hauraki Gulf and Coromandel Peninsula, on the south coast of North Island, and at the Antipodes Island Group.

Celleporaria agglutinans

Model validation

The mean AUC for *Celleporaria agglutinans* was 0.770, meaning the model is useful for predicting suitable habitat for this species. Test omission rate was reasonably low (12.7%), but test gain was also quite low, 0.459. Suspended particulate matter and tidal current were the most effective variables for predicting the distribution of the test data when predictive performance was measured using AUC, whilst surface water productivity, suspended particulate matter, and particularly tidal current were the most effective variables for predicting the distribution of the test data when predictive performance was measured using test gain. Removing SST gradient, dominant sediment type and wave height would

increase AUC very slightly, whilst removing median grain size, mixed layer depth, SST gradient and dominant sediment type would increase test gain.

Known and potential distribution

The majority of presence records for *Celleporaria agglutinans* occurred in Greater Cook Strait and Foveaux Strait, with smaller concentrations around the northern North Island, Hauraki Gulf, around the eastern North Island, Otago shelf and Puysegur 'Bank'. The distribution of presence records was much more extensive than the predicted distribution. Small areas of suitable habitat were predicted on the east coast of North Island, in Greater Cook Strait, in patches along South Island's east coast (including small, favourable patches in the southern Canterbury Bight and on Otago shelf). Much of the east coast of Stewart Island/Rakiura was of average-to-favourable habitat suitability. Areas around the Chatham Islands were predicted to be of average habitat suitability, with small areas along the coast predicted as favourable habitat. Predictions where there were no presence records occurred on the east coast of North Island, in the Canterbury Bight, Banks Peninsula, and Chatham Islands. Presence records occurred where habitat was not predicted around the eastern and northern North Island, off Kahurangi Point, and on Puysegur 'Bank'.

Celleporina grandis

Model validation

The mean AUC was 0.780, however test omission rate was high (20%) and test gain was quite low, (0.423), meaning the model should be used with some caution to predict the distribution of suitable habitat for *Celleporina grandis*. Mixed layer depth and SST in winter, and to a lesser extent, SST gradient, dominant sediment type and wave height were the most effective variables for predicting the distribution of the test data when predictive performance was measured using AUC. Mixed layer depth, SST in winter, dominant sediment type, and wave height were the most effective variables for predicting the distribution of the test data, when predictive performance was measured using test gain. Removing the variables surface water productivity, suspended particulate matter and particularly tidal current would increase AUC, whilst removing depth, median grain size, suspended particulate matter, and particularly surface water productivity and tidal current would increase test gain.

Known and potential distribution

Celleporina grandis presence records were mainly clustered in Greater Cook Strait, with smaller concentrations on Puysegur 'Bank', the western Chatham Rise, occasional points along the east and west coast of South Island, and one point near Manawatāwhi/Three Kings Islands. The presence records described well the predicted distribution. The most extensive predicted area of suitable habitat extended along the outer shelf/continental slope from Cook Strait in the north, to The Snares shelf in the south, and east along the Chatham Rise, almost to the Chatham Islands. This predicted area of suitable habitat was not contiguous with the shore (i.e. shallow habitat was unsuitable in most places), and increased in suitability in the middle. The other areas predicted as suitable habitat were off Puysegur 'Bank' (small favourable areas nested within a larger area of average habitat suitability), along the west coast of South Island (patchily average-to-high suitability), and Greater Cook Strait (patchily average-to-favourable habitat). Predictions of suitable habitat where there were no presence records occurred on the east coast of North Island, and in a band both on and off the shelf break from The Snares shelf towards the north-east, and along the coast north of Banks Peninsula. Presence records where there were no predictions of suitable habitat occurred at Manawatāwhi/Three Kings Islands and at the northern tip of Stewart Island/Rakiura.

Cinctipora elegans

Model validation

The mean AUC (0.825) and test gain (0.778) for *Cinctipora elegans* were quite high, however test omission rate was also high (25%), so that not all suitable areas of habitat for this species were predicted by the model. Suspended particulate matter, SST in winter, and tidal current were the most effective variables for predicting the distribution of the test data when predictive performance was measured using AUC, and mixed layer depth, suspended particulate matter, wave height, and particularly SST in winter and tidal current were the most effective variables for predicting the distribution of the test data, when predictive performance was measured using test gain. AUC would have increased slightly with the removal of SST gradient, and test gain would have increased with the removal of depth, median grain size, dominant sediment type, and particularly SST gradient.

Known and potential distribution

Cinctipora elegans presence records were concentrated in Greater Cook Strait, Puysegur 'Bank', and Foveaux Strait. Two records occurred on Otago shelf. The predictions of habitat suitability partially reflected the presence data. The most extensive area of highly suitable habitat was predicted around the northern Canterbury Bight, and around, and north of, Banks Peninsula. Large areas at Mernoo Bank and around the Bounty Islands were favourable. Smaller favourable areas were predicted in Greater Cook Strait, on Puysegur 'Bank', in Foveaux Strait and on the Chatham Rise. Notable predicted areas of suitable habitat where there were no presence records occurred in the Canterbury Bight–Banks Peninsula area, on Mernoo Bank and the Chatham Rise, at the Bounty Islands, and in eastern Cook Strait. Presence records where there were no predictions for suitable habitat occurred on the south coast of North Island.

Diaperoecia purpurascens

Model validation

The mean model AUC for *Diaperoecia purpurascens* was 0.765, and test omission rate was 14%, meaning the model can be used to predict the distribution of suitable habitat for this species. Test gain was 0.312. Depth, mixed layer depth, suspended particulate matter, SST in winter, and particularly tidal current were the most effective variables for predicting the distribution of the test data when predictive performance was measured using AUC. Tidal current was the most effective variable for predicting the distribution of the test data, when predictive performance was measured using test gain, but mixed layer depth, suspended particulate matter, SST in winter were also effective. The model AUC would increase slightly if surface water productivity, dominant sediment type and wave height were removed from the model, whilst removing depth, median grain size, surface water productivity, SST gradient, dominant sediment type and wave height would increase test gain.

Known and potential distribution

Presence records for *Diaperoecia purpurascens* were concentrated in Greater Cook Strait and Foveaux Strait, with a small cluster in the Hauraki Gulf, and occasional observations near Kermadec Ridge, around northern North Island and on Puysegur 'Bank'. The distribution of presence records only partially reflected the predicted distribution of suitable habitat. Highly suitable habitat was predicted in Greater Cook Strait, off Kahurangi Point, around and north of Banks Peninsula, and in Foveaux Strait. Favourable habitat was predicted on the west side of the northern North Island, on Mernoo Bank, around the Chatham and Bounty Islands and on Puysegur 'Bank'. The extensive areas of suitable habitat predicted at the Bounty and Chatham Islands, at Mernoo Bank, around Banks Peninsula, and off

the north-west coast of South Island had no associated presence records, whilst presence records with no predictions occurred on Kermadec Ridge.

Galeopsis porcellanicus

Model validation

The mean AUC of 0.767 for *Galeopsis porcellanicus* meant the model could be used to further understand the habitat distribution of this species, although test gain was low at 0.390, and test omission rate was high at 17.5%, so that some caution is necessary. Depth and SST in winter, but mainly tidal current were the most effective variables for predicting the distribution of the test data when predictive performance was measured using AUC. Depth, dominant sediment type and mainly tidal current were the most effective variables for predicting the distribution of the test data, when predictive performance was measured using test gain. Jackknifing showed AUC would increase with the removal from the model of surface water productivity, SST gradient, and wave height, whilst test gain would have increased with the removal of median grain size, mixed layer depth, surface water productivity, suspended particulate matter, SST in winter and particularly SST gradient and wave height.

Known and potential distribution

Presence records for *Galeopsis porcellanicus* were concentrated in the Hauraki Gulf, Greater Cook Strait, at the Antipodes Island Group and in Foveaux Strait, with less dense concentrations around the northern North Island, and on Puysegur 'Bank'. Predicted suitable habitat was sparse, and only partially reflected the distribution of presence records. The largest uninterrupted areas of suitable habitat were predicted offshore, with highly suitable areas at Campbell Island/Motu Ihupuku, and favourable areas around the Bounty and Chatham Islands. Nearshore, parts of Foveaux Strait, Greater Cook Strait, and off Kahurangi Point were predicted as highly suitable habitat, interspersed with favourable habitat, and areas of average suitability. Smaller areas were predicted as favourable in the Hauraki Gulf, Hawke Bay, and along South Island's east coast. Suitable habitat was predicted where there were no presence records in Hawke Bay, through the northern and eastern parts of Greater Cook Strait, the east coast of South Island, around the Chatham and Bounty Islands, and north-east of Campbell Island/Motu Ihupuku. Presence records occurred where no suitable habitat was predicted on Puysegur 'Bank', and around the northern North Island.

Hippomenella vellicata

Model validation

For *Hippomenella vellicata* the mean AUC was 0.707, meaning the models can be used to further consider the potential distribution of suitable habitat for this species, however test gain was low (0.221) and test omission rate was high (18.3%), meaning caution is necessary. Mixed layer depth, dominant sediment type, and tidal current were the most effective variables for predicting the distribution of the test data when predictive performance was measured using AUC, whilst mixed layer depth, wave height, and particularly tidal current were the most effective variables for predicting the distribution of the test data when predictive performance was measured using test gain. AUC would increase if median grain size, surface water productivity, suspended particulate matter, SST gradient, and SST in winter were removed from the model, and test gain would increase if median grain size, surface water productivity, suspended particulate matter, SST gradient, SST in winter and dominant sediment type were removed from the model. On its own, tidal current generated more test gain than, and as much AUC as, the full model.

Known and potential distribution

Presence records for *Hippomenella vellicata* were concentrated in Greater Cook Strait, with smaller concentrations around northern North Island, in Foveaux Strait, along Puysegur 'Bank' and the Fiordland coast, with occasional records on Kermadec Ridge, west of North Island, along the west coast of South Island, and on Otago shelf. The predicted distribution of suitable habitat partially reflected the distribution of presence records. A small patch of Greater Cook Strait was predicted as highly suitable habitat, and a much larger area around the north-eastern coast of South Island, to Kahurangi Point, was predicted as favourable habitat for this species. Patches off Banks Peninsula and Mernoo Bank were also predicted as favourable habitat, with much smaller areas predicted as favourable in the Canterbury Bight and Foveaux Strait. Small areas of average habitat suitability were predicted west of the Manawatāwhi/Three Kings Islands, around the Chatham Islands, and off Puysegur 'Bank'. Suitable habitat for *Hippomenella vellicata* was predicted where there were no presence records off Kahurangi Point, the northern east coast of South Island, on Mernoo Bank, and at the Chatham Islands. Presence records occurred where no habitat was predicted as suitable on the Kermadec Ridge, through much of the Hauraki Gulf, and along the west coast of South Island, including Fiordland and much of Puysegur 'Bank'.

Hornera foliacea

Model validation

The mean AUC was 0.881 and test omission rate was 10%, meaning that on this basis alone the model could be used to predict distributions of suitable habitat for this species with confidence. Test gain was also high (1.239). Despite the high AUC and test gain, the model did not predict any cells as suitable for this species, and so was excluded from further analyses. Suspended particulate matter was the most effective variable for predicting the distribution of the test data when predictive performance was measured using both AUC and test gain. AUC would increase if median grain size, SST gradient, SST in winter, dominant sediment type and wave height were removed, and on its own, SST in winter achieved an AUC value almost as high as that of the full model. Test gain would be increased by the removal of median grain size, mixed layer depth, surface water productivity, SST gradient, SST in winter, dominant sediment type, tidal current and wave height.

Known and potential distribution

Presence records for *Hornera foliacea* were concentrated in Foveaux Strait, with occasional presence records on the south coast of North Island, on Otago shelf and Puysegur 'Bank'. No areas were predicted as suitable habitat for this species.

Smittoidea maunganuiensis

Model validation

The mean AUC was 0.799 for *Smittoidea maunganuiensis*, and test omission rate was 11.7%, so the model can be used to further consider the distribution of predicted suitable habitat and environmental controls. The test gain was 0.803. Depth, SST in winter, dominant sediment type, and tidal current were the most effective variables for predicting the distribution of the test data when predictive performance was measured using AUC. Depth, SST gradient, SST in winter, dominant sediment type and tidal current were the most effective variables for predicting the distribution of the test data, when predictive performance was measured using test gain. AUC would increase with the removal of

median grain size and wave height, whilst test gain would increase if median grain size, mixed layer depth, surface water productivity, suspended particulate matter and wave height were removed.

Known and potential distribution

Presence records of *Smittoidea maunganuiensis* were scattered around coastal parts of the ECS, with a concentration of points around the Antipodes Island Group and in the Hauraki Gulf, and occasional records on the Fiordland coast, Puysegur 'Bank', Foveaux Strait, in Greater Cook Strait, and around northern North Island. The predicted distribution of habitat suitability only partially reflected the distribution of presence records. Highly suitable habitat was predicted around the Chatham Islands and along parts of the South Island's east coast. Favourable habitat was predicted in patches along coastal parts of the Northland Peninsula, in Hawke Bay, in various small patches in Greater Cook Strait, off Kahurangi Point, through the central and southern Canterbury Bight, in small patches through Foveaux Strait and north-east of Campbell Island/Motu Ihupuku. Average suitable habitat surrounded much of the favourable habitat, notably in Greater Cook Strait, on Otago shelf, through Foveaux Strait and around the Bounty Islands.

Suitable habitat for *Smittoidea maunganuiensis* was predicted where there were no presence records in sheltered bays along the east coast of the Northland Peninsula, in Hawke Bay, west of Taranaki, at Kahurangi Point, along much of the east coast of South Island and around the Chatham Islands. Presence records occurred but suitable habitat was not predicted around the northern North Island, along the Fiordland coast, at Puysegur 'Bank', and at the Antipodes Island Group.
